# E3 ligase LMO7 promotes ALKBH2 ubiquitination to sensitize MGMT-deficient glioblastoma to temozolomide

**DOI:** 10.1038/s41419-026-09073-6

**Published:** 2026-06-30

**Authors:** Zhiming Sun, Yunfang Deng, Yue Liu, Liyuan Zeng, Chujing He, Ye Liu, Zhaohui Liu, Jiabing Li, Ying Sheng, Yuqiao Cai, Yuyao Xue, Yu Zhao

**Affiliations:** 1 The National & Local Joint Engineering Laboratory of Animal Peptide Drug Development, College of Life Sciences, Hunan Normal University, Changsha, Hunan 410081 China; 2 Peptide and Small Molecule Drug R&D Platform, Furong Laboratory, Hunan Normal University, Changsha, Hunan 410081 China

**Keywords:** DNA adducts, CNS cancer, Ubiquitylated proteins

## Abstract

Temozolomide (TMZ) resistance in glioblastoma (GBM) is often linked to high MGMT protein levels, but MGMT-deficient tumors can still acquire resistance, implicating MGMT-independent repair pathways. Here, we identify an oxidative stress-responsive LMO7–ALKBH2 ubiquitination mechanism that regulates ALKBH2 stability and modulates alkylation damage tolerance in GBM. The E3 ligase LMO7 directly binds ALKBH2 and promotes K48-linked polyubiquitination and proteasomal degradation of ALKBH2, with Lys7 serving as a major ubiquitin acceptor site. Oxidative stress enhances LMO7-dependent ALKBH2 ubiquitination through NEDD8-dependent cullin neddylation, reducing TMZ-induced ALKBH2 accumulation and increasing TMZ sensitivity. The K7R substitution attenuates LMO7- and oxidative stress-induced ALKBH2 polyubiquitination and degradation and weakens LMO7-dependent TMZ sensitization. Functionally, ALKBH2 depletion sensitizes MGMT-deficient GBM cells to TMZ and methyl methanesulfonate, whereas ALKBH2 re-expression restores alkylator tolerance. In patient datasets, ALKBH2 protein levels are elevated in GBM and inversely associated with MGMT protein levels, an association not detected at the mRNA level. High *ALKBH2* expression in chemotherapy-treated IDH-wild-type glioma and low *LMO7* expression in the CGGA astrocytoma cohort are each associated with shorter overall survival. Together, these results indicate that oxidative stress-coupled LMO7-dependent polyubiquitination of ALKBH2 modulates TMZ sensitivity in GBM, particularly in MGMT-deficient tumors.

**Figure:**
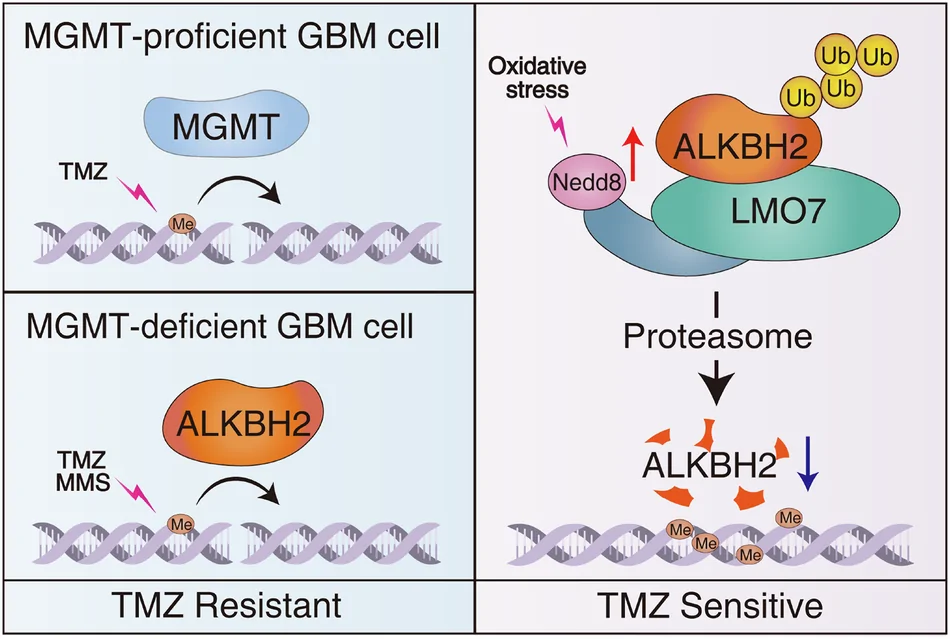

## Introduction

Glioblastoma (GBM) is among the most aggressive primary malignant brain tumors, and standard-of-care therapy includes radiotherapy plus temozolomide (TMZ) [1]. Although TMZ is widely used, therapeutic responses are heterogeneous, and resistance frequently emerges during treatment [2]. TMZ generates several DNA alkylation lesions, including *O*^6^-methylguanine (*O*^6^-meG), a low-abundance but highly cytotoxic adduct that is directly repaired by *O*^6^-methylguanine-DNA methyltransferase (MGMT) [3, 4]. Accordingly, MGMT promoter methylation status and MGMT protein level are widely used clinically to stratify TMZ response [5, 6]. However, many GBM tumors contain low or undetectable MGMT protein levels, often due to MGMT promoter methylation-mediated silencing. Even in this setting, resistance to TMZ can develop, and its clinical benefit remains limited [7, 8]. This clinical pattern indicates that MGMT-independent mechanisms can reduce TMZ cytotoxicity and raises the question of which TMZ-induced lesions and repair pathways sustain alkylation damage tolerance in MGMT-deficient GBM.

In addition to *O*^6^-meG, TMZ generates a spectrum of MGMT-independent N-alkyl DNA adducts that can induce replication stress and impair cell viability [9]. In short-term MGMT-deficient models, the cellular response to TMZ also reflects contributions from concomitant N-alkyl lesions (principally N3-methyladenine and N7-methylguanine) that are repaired via MGMT-independent pathways, including base excision repair (BER) and AlkB-family Fe(II)/2-oxoglutarate–dependent oxidative demethylation [10–12]. Among AlkB-family enzymes, ALKBH2 preferentially repairs duplex DNA and removes replication-fork–blocking N-alkyl lesions, including N1-methyladenine (1-meA) and 3-methylcytosine (3-meC), thereby limiting replication-fork stalling under alkylation stress [13, 14]. *ALKBH2* mRNA levels have been reported to be higher in GBM than in lower-grade gliomas and normal brain tissue [15]. Multiple upstream factors regulate *ALKBH2* at the mRNA level in glioma models, and independent studies have linked higher ALKBH2 activity to reduced TMZ cytotoxicity in glioma cells [15–19]. Pharmacologic inhibition of ALKBH2 has also been examined as a strategy to increase susceptibility to alkylation stress in glioma models [20–22]. These reports indicate a role for ALKBH2 in MGMT-independent alkylation damage tolerance, whereas the mechanisms that maintain ALKBH2 protein levels in tumor cells remain incompletely understood.

Ubiquitin-dependent proteasomal degradation controls protein stability, with substrate recognition mediated by E3 ubiquitin ligases [23]. Among these ligases, cullin-RING ligases (CRLs), including Skp1-Cullin-F-box (SCF) complexes that use distinct F-box substrate adaptors to recruit substrates, mediate proteasome-dependent turnover during therapy-induced stress [24–27]. Multiple DNA repair enzymes, including AlkB-family dioxygenases, undergo ubiquitination-dependent turnover, linking regulated protein stability to cellular tolerance to alkylation damage [28–30]. However, the mechanisms that control ALKBH2 protein stability in GBM remain incompletely characterized. LMO7 (LIM domain only 7, also annotated as FBXO20) has been reported to function as an SCF-type E3 ligase adaptor involved in substrate recognition, with context-dependent functions across tumor types [31–33]. In glioma, reduced *LMO7* expression has been reported in GBM, and higher *LMO7* expression has been associated with less aggressive disease features [34, 35]. A prior proteomic analysis detected ALKBH2 together with LMO7 [36], suggesting LMO7 may act as an ALKBH2-associated regulatory factor. Whether LMO7 regulates ALKBH2 protein stability and whether this relationship contributes to alkylation damage tolerance in glioma remain unclear.

In this study, we identify an oxidative stress-responsive LMO7–ALKBH2 ubiquitination pathway that regulates ALKBH2 protein stability and modulates TMZ sensitivity in GBM. LMO7 directly binds ALKBH2 and promotes K48-linked polyubiquitination of ALKBH2 at Lys7, leading to proteasomal degradation. Oxidative stress activates NEDD8-dependent cullin neddylation and augments this LMO7-dependent pathway, thereby limiting ALKBH2-mediated alkylation damage tolerance and increasing TMZ sensitivity. The Lys7-to-Arg substitution attenuates LMO7- and oxidative stress-induced ALKBH2 degradation and reduces the TMZ-sensitizing effect of this pathway. These data indicate that modulation of the LMO7–ALKBH2 axis changes cellular tolerance to alkylation-induced DNA lesions and provides a basis for evaluating TMZ-based combination strategies, particularly in MGMT-deficient GBM.

## Results

### ALKBH2 protein levels are increased in glioblastoma and inversely correlated with MGMT protein levels

**Fig. 1:**
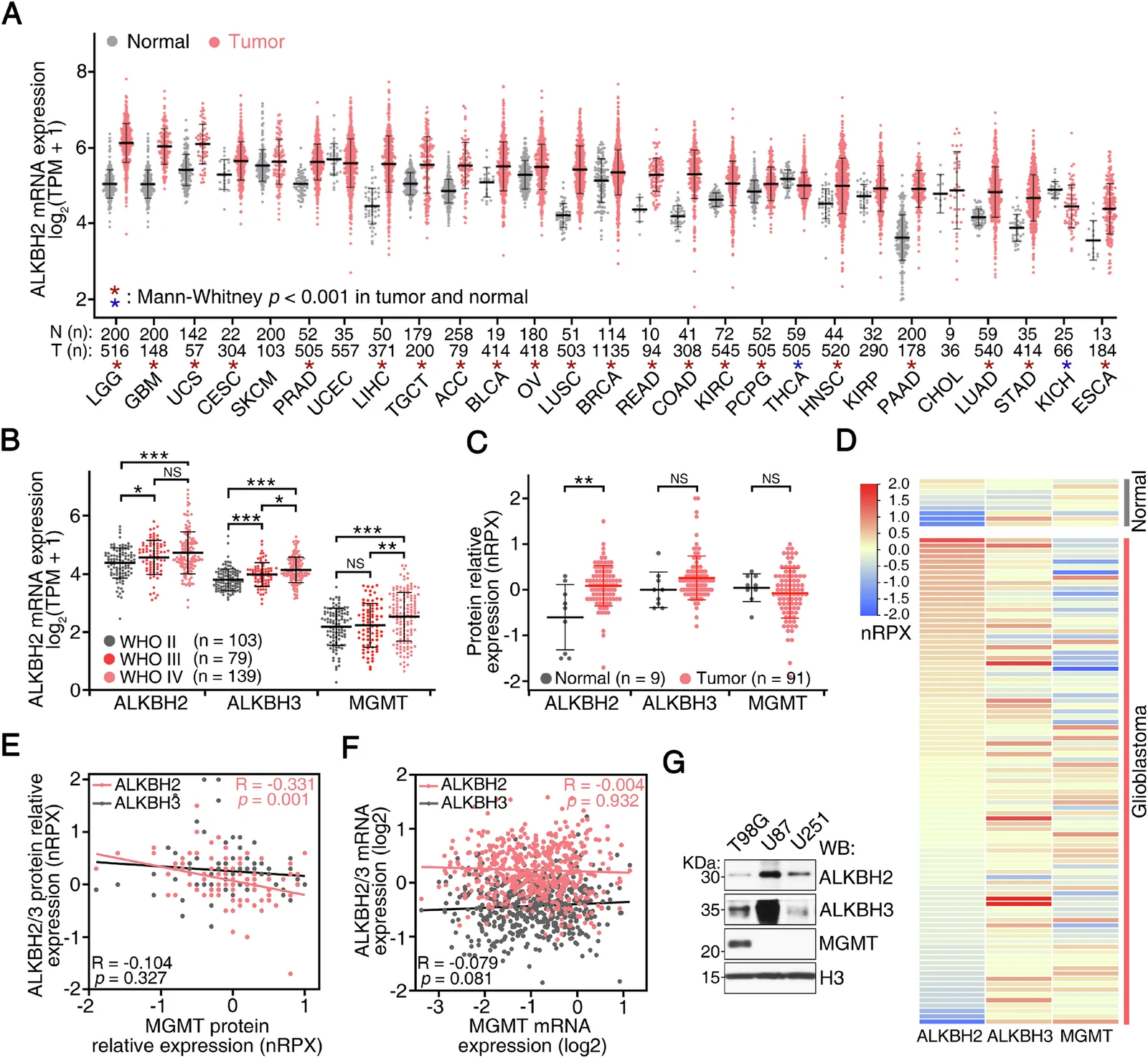
ALKBH2 protein levels are increased in glioblastoma and inversely correlated with MGMT protein levels. **A** Pan-cancer analysis of *ALKBH2* mRNA expression across The Cancer Genome Atlas (TCGA) cancer types, comparing tumor tissues with normal tissues from TCGA and Genotype-Tissue Expression (GTEx); expression values are shown as log2(TPM + 1). **B** mRNA expression of *ALKBH2*, *ALKBH3*, and *MGMT* in glioma samples from the Chinese Glioma Genome Atlas (CGGA), stratified by WHO grade II, III, or IV; each dot represents one patient sample. **C** Relative protein levels of ALKBH2, ALKBH3, and MGMT in normal brain tissues (*n* = 9) and glioblastoma (GBM) samples (*n* = 91) from the CPTAC proteomic dataset, shown as normalized relative protein expression (nRPX) values; this cohort was not stratified by MGMT promoter methylation status. **D** Heatmap of ALKBH2, ALKBH3, and MGMT protein levels across individual normal brain and GBM samples from the CPTAC cohort. **E** Pearson correlation analysis of MGMT protein levels with ALKBH2 or ALKBH3 protein levels in GBM samples based on CPTAC nRPX data; Pearson correlation coefficients (*R*) and *p* values are indicated. **F** Pearson correlation analysis of *MGMT* mRNA expression with *ALKBH2* or *ALKBH3* mRNA expression in glioma samples from TCGA; expression values are shown on a log2 scale. **G** ALKBH2, ALKBH3, and MGMT protein levels in the indicated GBM cell lines were analyzed by western blotting (WB); histone H3 was used as a loading control. Data are presented as mean ± SD where applicable. Statistical significance was determined using Mann–Whitney *U* tests in (**A**), unpaired two-tailed Student’s *t*-tests in (**B**, **C**), and Pearson correlation tests in (**E**, **F**). *p < 0.05, **p < 0.01, ***p < 0.001; NS not significant.

To profile *ALKBH2* expression across human cancers, we analyzed The Cancer Genome Atlas (TCGA) pan-cancer transcriptomic data. *ALKBH2* mRNA was detected across all samples, with higher levels in low-grade glioma (LGG), glioblastoma (GBM), and uterine carcinosarcoma. Among these three tumor types, LGG and GBM showed the largest tumor–normal differences (Fig. 1A). In Chinese Glioma Genome Atlas (CGGA) glioma cohorts, *ALKBH2* mRNA levels increased with World Health Organization (WHO) grade, similar to the related alkylation repair factors *ALKBH3* and *MGMT* (Fig. 1B). At the protein level, proteomic analysis of the Clinical Proteomic Tumor Analysis Consortium (CPTAC) cohort showed higher ALKBH2 protein levels in GBM than in normal brain, whereas neither ALKBH3 nor MGMT showed a comparable increase in this unstratified proteomic cohort (Fig. 1C). Matched MGMT promoter methylation annotations were not available for this cohort, precluding stratified assessment of MGMT protein levels in promoter-unmethylated GBM. A heatmap of CPTAC samples ranked by ALKBH2 protein levels showed uniformly low ALKBH2 protein levels in normal samples and heterogeneous ALKBH2 protein levels in tumors, spanning ALKBH2-low and ALKBH2-high groups. MGMT-low tumors were enriched within the ALKBH2-high portion of the cohort (Fig. 1D).

Consistent with this pattern, CPTAC proteomic data showed a significant inverse correlation between ALKBH2 and MGMT protein levels, whereas ALKBH3 did not correlate with MGMT (Fig. 1E). In contrast, no inverse correlation between *ALKBH2* and *MGMT* was detected at the mRNA level in TCGA glioma transcriptomic data (Fig. 1F), consistent with a post-transcriptional basis for this protein-level relationship. In glioma cell models, ALKBH2 protein levels were higher in MGMT-deficient U87 and U251 cells than in MGMT-proficient T98G cells, whereas ALKBH3 did not follow this pattern (Fig. 1G). Collectively, these results indicate that ALKBH2 protein levels are elevated in GBM and inversely associated with MGMT at the protein level.

### ALKBH2 is associated with survival in chemotherapy-treated glioma and promotes alkylating-agent resistance in MGMT-deficient glioma

**Fig. 2:**
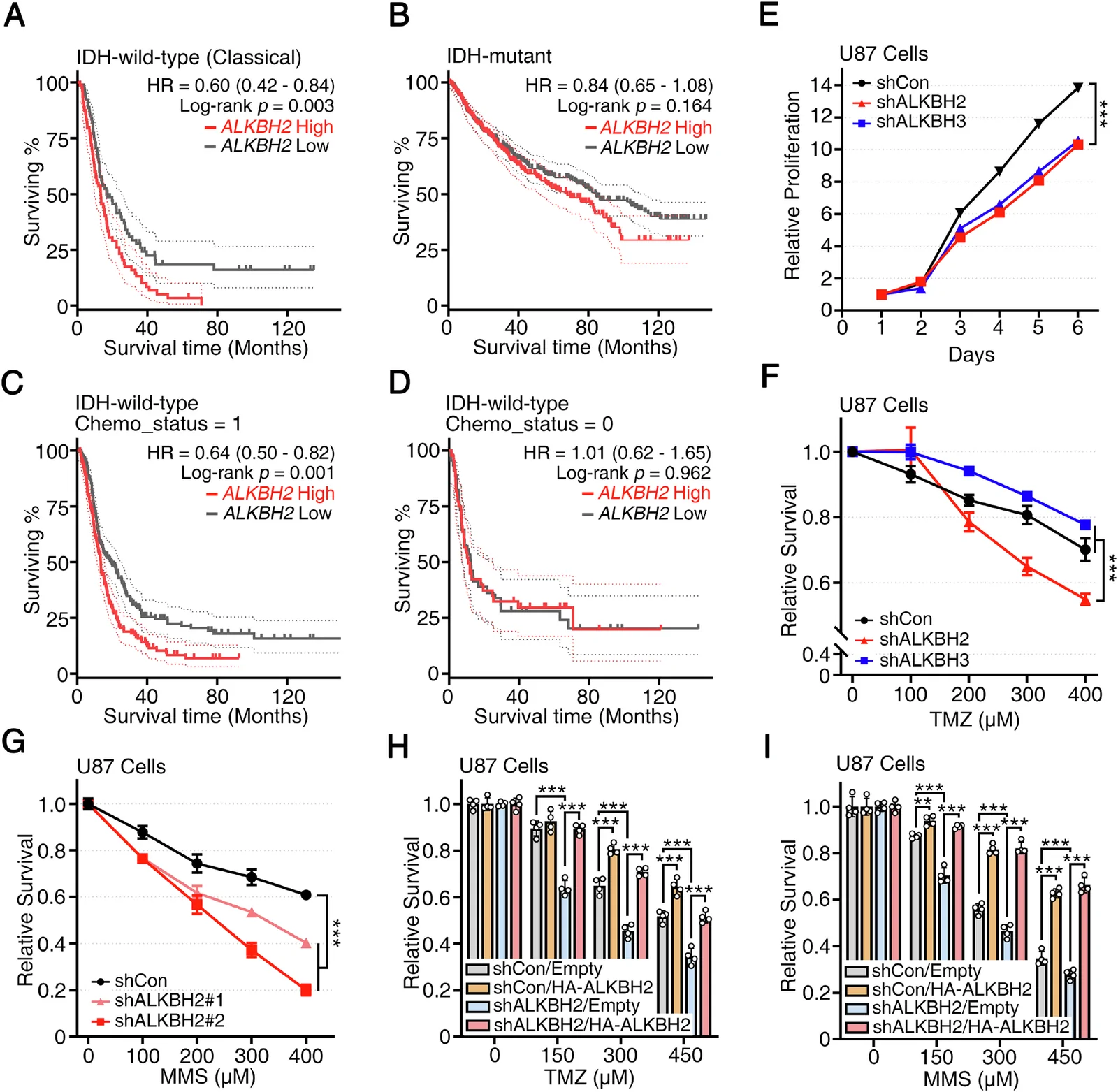
ALKBH2 is associated with survival in chemotherapy-treated glioma and promotes alkylating-agent resistance in MGMT-deficient glioma. **A** Kaplan–Meier overall survival curves for patients with classical IDH-wild-type glioma stratified by *ALKBH2* mRNA expression in the Chinese Glioma Genome Atlas (CGGA) database (*ALKBH2* high, *n* = 78; *ALKBH2* low, *n* = 79), with hazard ratio (HR) and log-rank *p* value indicated. **B** Kaplan–Meier overall survival curves for patients with IDH-mutant glioma stratified by *ALKBH2* expression in CGGA (*ALKBH2* high, *n* = 256; *ALKBH2* low, *n* = 257). **C** Kaplan–Meier overall survival curves for patients with IDH-wild-type glioma annotated as chemotherapy-treated subgroup (Chemo_status = 1) and stratified by *ALKBH2* expression in CGGA/GlioVis (*ALKBH2* high, *n* = 149; *ALKBH2* low, *n* = 149). **D** Kaplan–Meier overall survival curves for patients with IDH-wild-type glioma annotated as chemotherapy-untreated subgroup (Chemo_status = 0) and stratified by *ALKBH2* expression in CGGA/GlioVis (*ALKBH2* high, *n* = 44; *ALKBH2* low, *n* = 45). Analyses in **C**, **D** were not stratified by MGMT promoter methylation status, and patients with unavailable chemotherapy status were excluded. **E** CCK-8 analysis of proliferation in MGMT-deficient U87 glioma cells expressing control shRNA (shCon), shALKBH2, or shALKBH3 at the indicated time points. **F** CCK-8 analysis of relative survival in MGMT-deficient U87 cells expressing shCon, shALKBH2, or shALKBH3 after TMZ treatment for 48 h. **G** CCK-8 analysis of relative survival in MGMT-deficient U87 cells expressing shCon or two independent shRNAs targeting *ALKBH2* after MMS treatment for 48 h. H, I CCK-8 analysis of relative survival in MGMT-deficient U87 cells expressing shCon or shALKBH2 and reconstituted with empty vector or HA-ALKBH2 after treatment with TMZ (**H**) or MMS (**I**) for 48 h. Statistical significance in **A**–**D** was assessed using log-rank tests. Data in **E**–**I** are presented as mean ± SD. Statistical significance in **E**–**I** was determined using unpaired two-tailed Student’s *t*-tests. *p < 0.05, **p < 0.01, ***p < 0.001.

To assess the clinical relevance of ALKBH2 in glioma, we analyzed overall survival in the CGGA cohorts. Higher *ALKBH2* expression was associated with shorter overall survival in patients with isocitrate dehydrogenase (IDH)-wild-type glioma of the classical molecular subtype (Fig. 2A), whereas this association was not detected in patients with IDH-mutant glioma (Fig. 2B). This contrast is consistent with prior evidence that IDH mutations can alter responses to alkylating agents through inhibition of ALKBH-family enzymes [19], potentially attenuating the prognostic impact of ALKBH2 in this subgroup. We therefore focused on IDH-wild-type glioma and stratified patients by the recorded chemotherapy status in the CGGA/GlioVis dataset, without stratifying by MGMT promoter methylation status. In patients annotated as chemotherapy-treated (Chemo_status = 1), high *ALKBH2* expression was associated with shorter overall survival (Fig. 2C), whereas no such association was detected in patients annotated as chemotherapy-untreated (Chemo_status = 0) (Fig. 2D). Patients with unknown chemotherapy status were excluded. This retrospective analysis was based on the recorded Chemo_status field and did not involve constructing a matched cohort. Accordingly, the Chemo_status = 0 subgroup was interpreted as a database-defined comparator in the survival analysis.

We next examined the function of ALKBH2 in glioma cell models. In MGMT-deficient U87 cells, depletion of ALKBH2 or ALKBH3 reduced basal proliferation, and knockdown efficiency was verified by western blotting (WB) (Fig. 2E and Supplementary Fig. 1A). Under TMZ treatment, ALKBH2 depletion increased TMZ sensitivity, whereas ALKBH3 depletion did not produce a comparable effect (Fig. 2F). ALKBH2 depletion also reduced survival after exposure to methyl methanesulfonate (MMS), an alkylating agent that predominantly induces N-alkyl DNA adducts [11, 29], with knockdown confirmed by WB (Fig. 2G and Supplementary Fig. 1B). Re-expression of ALKBH2 in ALKBH2-depleted U87 cells restored viability after TMZ or MMS treatment, whereas empty vector did not, with depletion and reconstitution confirmed by WB (Fig. 2H, I and Supplementary Fig. 1C). In MGMT-proficient T98G cells, depletion of ALKBH2 or ALKBH3 did not alter TMZ sensitivity despite verified knockdown, whereas MGMT depletion increased TMZ sensitivity as a positive control (Supplementary Fig. 1D, E). Collectively, these data indicate that ALKBH2 promotes alkylating-agent resistance in MGMT-deficient glioma cells, and that higher ALKBH2 expression is associated with shorter overall survival in chemotherapy-treated, IDH-wild-type gliomas.

### LMO7 directly binds ALKBH2

To identify candidate E3 ubiquitin ligases involved in ALKBH2 regulation, we re-analyzed published proteomic datasets for proteins co-identified with ALKBH2 [36]. Based on total and unique peptide counts, this analysis prioritized several candidate ALKBH2-associated E3 ligases, including LMO7, UBR5, and RANBP2 (Supplementary Fig. 2A). TRIM28 also ranked among the top candidates in this re-analysis, as described in related preprint work [37]. In proteomic data from the CPTAC GBM cohort, LMO7 protein levels were inversely correlated with ALKBH2, whereas UBR5 and RANBP2 showed positive correlations with ALKBH2 (Supplementary Fig. 2B). In the same dataset, LMO7 showed no correlation with ALKBH3 or MGMT (Supplementary Fig. 2C), indicating that this inverse association was selective for ALKBH2 among the tested repair factors.

**Fig. 3:**
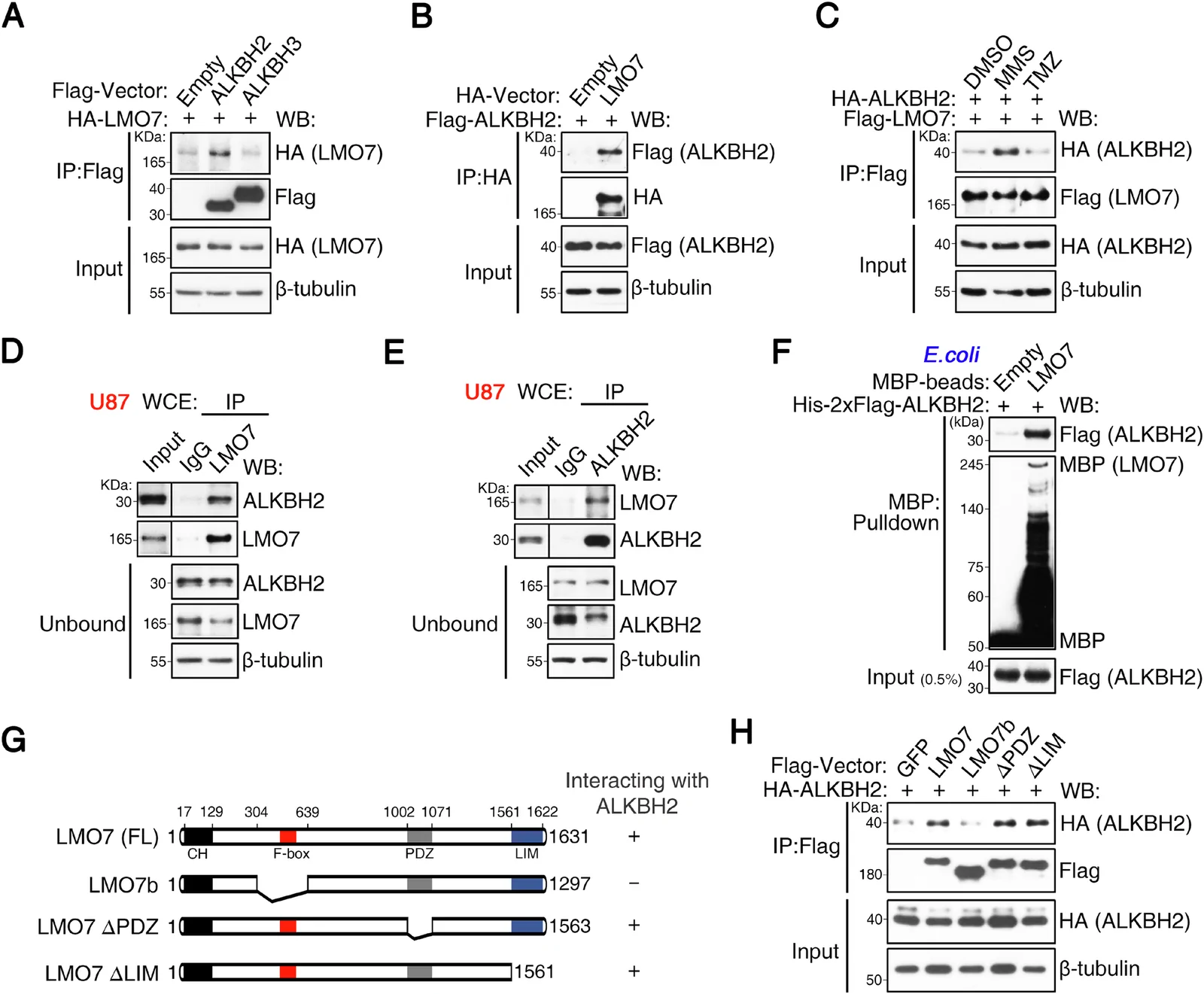
LMO7 directly binds ALKBH2. **A** Co-immunoprecipitation (co-IP) followed by western blotting (WB) in HEK293T cells expressing HA-LMO7 together with empty Flag vector, Flag-ALKBH2, or Flag-ALKBH3. Flag IP was followed by WB. **B** Reciprocal co-IP followed by WB in HEK293T cells expressing Flag-ALKBH2 together with empty HA vector or HA-LMO7. HA IP was followed by WB. **C** Co-IP followed by WB in HEK293T cells expressing HA-ALKBH2 and Flag-LMO7 after treatment with DMSO, MMS, or TMZ. Flag IP was followed by WB. **D**, **E** Reciprocal endogenous co-IP analysis of LMO7 and ALKBH2 in U87 cells. Endogenous LMO7 (**D**) or ALKBH2 (**E**) was immunoprecipitated with the indicated antibodies, with IgG used as a negative control. Input, IP, and unbound fractions were analyzed by WB to detect endogenous LMO7 and ALKBH2. Vertical lines between the input and IP lanes indicate different exposures of the same blot. **F** In vitro pull-down assay using recombinant His-2×Flag-ALKBH2 and MBP or MBP-LMO7 purified from *E. coli*. Bound proteins were analyzed by WB after MBP pull-down. **G** Schematic representation of full-length LMO7 and the indicated deletion mutants. Binding to ALKBH2 is summarized on the right. **H** Domain-mapping co-IP followed by WB in HEK293T cells expressing HA-ALKBH2 together with Flag-tagged GFP, full-length LMO7, LMO7b, LMO7 ΔPDZ, or LMO7 ΔLIM. Flag IP was followed by WB.

We next tested whether LMO7 associates with ALKBH2 by co-immunoprecipitation (co-IP) in HEK293T cells. Immunoprecipitation (IP) of Flag-ALKBH2 recovered HA-LMO7, whereas IP of Flag-ALKBH3 did not recover detectable HA-LMO7 (Fig. 3A). Reciprocal IP of HA-LMO7 recovered Flag-ALKBH2 (Fig. 3B). MMS increased the LMO7–ALKBH2 association, whereas TMZ produced no detectable change (Fig. 3C). Endogenous reciprocal co-IP in U87 cells supported the same association (Fig. 3D, E). Input and unbound fractions verified the presence of LMO7 and ALKBH2 in the lysates used for IP. An MBP pull-down assay using purified recombinant proteins further indicated a direct interaction between LMO7 and ALKBH2 (Fig. 3F).

To map the LMO7 regions required for ALKBH2 association, we generated Flag-tagged LMO7 constructs, including full-length LMO7, LMO7b, which lacks the F-box region, and ΔPDZ and ΔLIM deletion mutants (Fig. 3G). Co-IP assays in HEK293T cells showed that full-length LMO7 and the ΔPDZ or ΔLIM mutants associated with HA-ALKBH2, whereas LMO7b showed no detectable association (Fig. 3H). Collectively, these data indicate that LMO7 directly binds ALKBH2 and that MMS increases the LMO7–ALKBH2 association.

### LMO7 promotes Lys7-dependent K48-linked polyubiquitination of ALKBH2

**Fig. 4:**
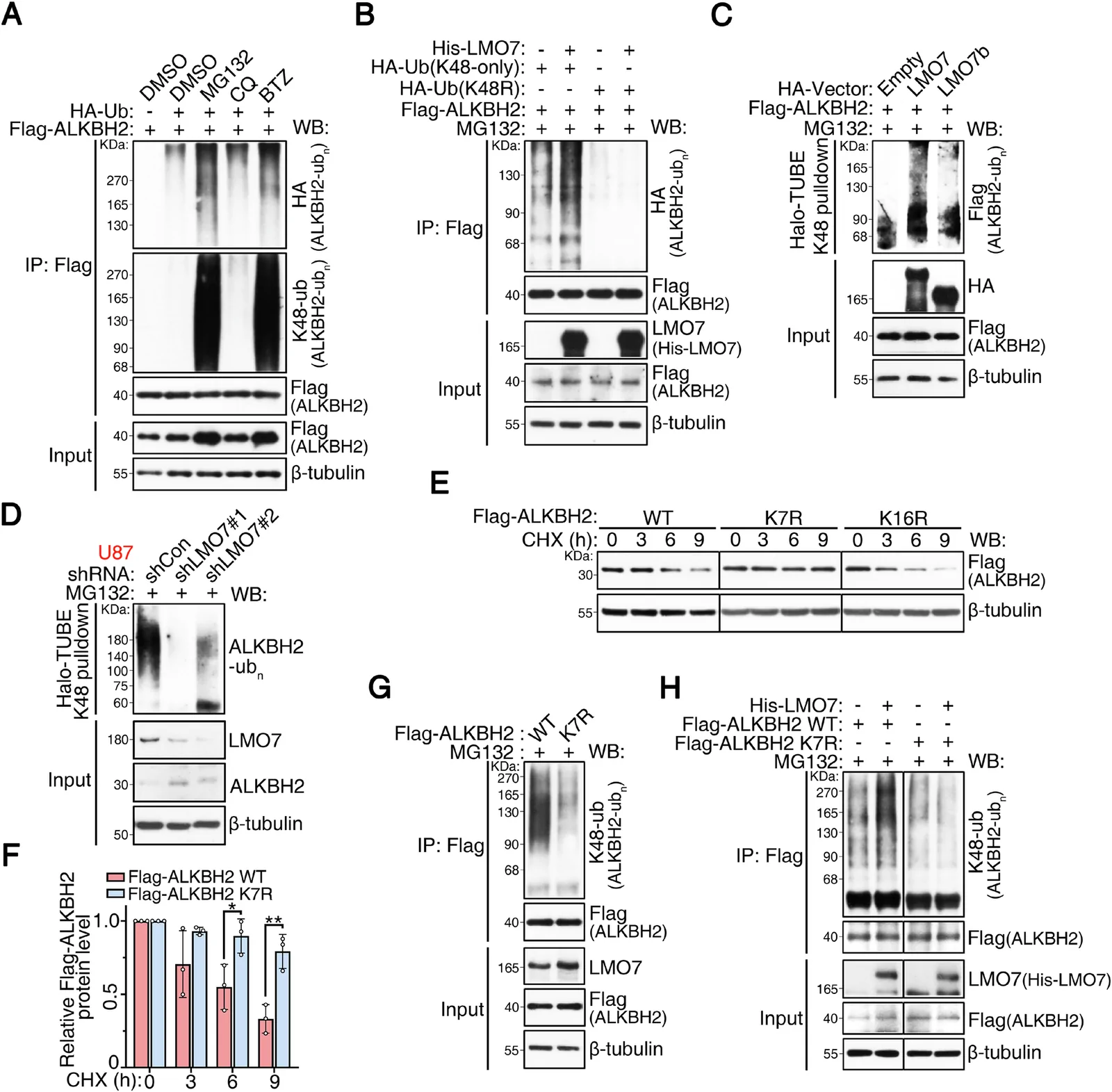
LMO7 promotes Lys7-dependent K48-linked polyubiquitination and proteasomal degradation of ALKBH2. **A** HEK293T cells expressing Flag-ALKBH2 and HA-tagged ubiquitin (HA-Ub) were treated overnight with DMSO, MG132 (10 μM), chloroquine (CQ, 20 μM), or bortezomib (BTZ, 100 nM). Flag IP was followed by WB. **B** HEK293T cells expressing Flag-ALKBH2, His-LMO7, and HA-Ub(K48-only) or HA-Ub(K48R) were treated with MG132. Flag IP was followed by WB. **C** HEK293T cells stably expressing Flag-ALKBH2 were transfected with empty vector, HA-LMO7, or HA-LMO7b and treated with MG132. K48-linked polyubiquitinated proteins were enriched using K48-specific Halo-TUBE beads and analyzed by WB. **D** U87 cells transduced with shCon or two independent shRNAs targeting LMO7 were treated with MG132. K48-linked polyubiquitinated proteins were enriched using K48-specific Halo-TUBE beads and analyzed by WB. **E** HEK293T cells expressing Flag-ALKBH2 wild-type (WT), K7R, or K16R were treated with cycloheximide (CHX, 150 μg/mL) for the indicated times, and Flag-ALKBH2 protein levels were analyzed by WB. **F** Quantification of Flag-ALKBH2 WT and K7R protein levels from the CHX chase experiments shown in (E). **G** HEK293T cells expressing Flag-ALKBH2 WT or K7R were treated with MG132. Flag IP was followed by WB to analyze K48-linked polyubiquitination of ALKBH2. **H** HEK293T cells expressing Flag-ALKBH2 WT or K7R together with empty vector or His-LMO7 were treated with MG132. Flag IP was followed by WB to analyze K48-linked polyubiquitination of ALKBH2. Data are presented as mean ± SD. Statistical significance was determined using unpaired two-tailed Student’s *t*-tests. *p < 0.05, **p < 0.01.

To examine the functional consequences of the LMO7–ALKBH2 interaction, we first tested whether ALKBH2 undergoes ubiquitination. In HEK293T cells stably expressing Flag-ALKBH2, the proteasome inhibitors MG132 and bortezomib increased polyubiquitinated ALKBH2, whereas the lysosomal inhibitor chloroquine (CQ) had no comparable effect. Using a K48-linkage-specific antibody, we detected increased K48-linked polyubiquitin chains on ALKBH2 after proteasome inhibition (Fig. 4A). LMO7 co-expression increased K48-linked polyubiquitination of ALKBH2 in assays using HA-Ub(K48-only), whereas no increase was detected with HA-Ub(K48R) (Fig. 4B). Halo-TUBE enrichment of K48-linked polyubiquitinated proteins further showed that full-length LMO7 increased K48-linked polyubiquitinated ALKBH2, whereas LMO7b had a weaker effect under the same conditions (Fig. 4C). Conversely, depletion of LMO7 in U87 cells reduced K48-linked polyubiquitinated ALKBH2 (Fig. 4D).

To identify the ubiquitin acceptor site contributing to ALKBH2 ubiquitination, we surveyed ubiquitination annotations in PhosphoSitePlus [38] and prioritized recurrently reported lysines. Lys7 emerged as a top candidate and was selected for functional testing, with Lys16 included as a control residue based on its annotation as a putative small ubiquitin-like modifier (SUMO) acceptor site. In cycloheximide (CHX) chase assays, the K7R substitution prolonged the half-life of Flag-ALKBH2 relative to wild-type (WT) ALKBH2, whereas K16R showed a turnover profile similar to WT (Fig. 4E, F). Consistent with increased stability, K7R reduced K48-linked polyubiquitination relative to WT ALKBH2 in a Flag IP followed by WB using a K48-linkage-specific antibody (Fig. 4G). Moreover, LMO7 co-expression increased K48-linked polyubiquitination of WT ALKBH2, whereas this increase was attenuated for the K7R mutant in the same assay (Fig. 4H). Collectively, these results indicate that LMO7 increases K48-linked polyubiquitination of ALKBH2 and that Lys7 serves as a major ubiquitin acceptor site.

### LMO7 promotes proteasome-dependent degradation of ALKBH2

**Fig. 5:**
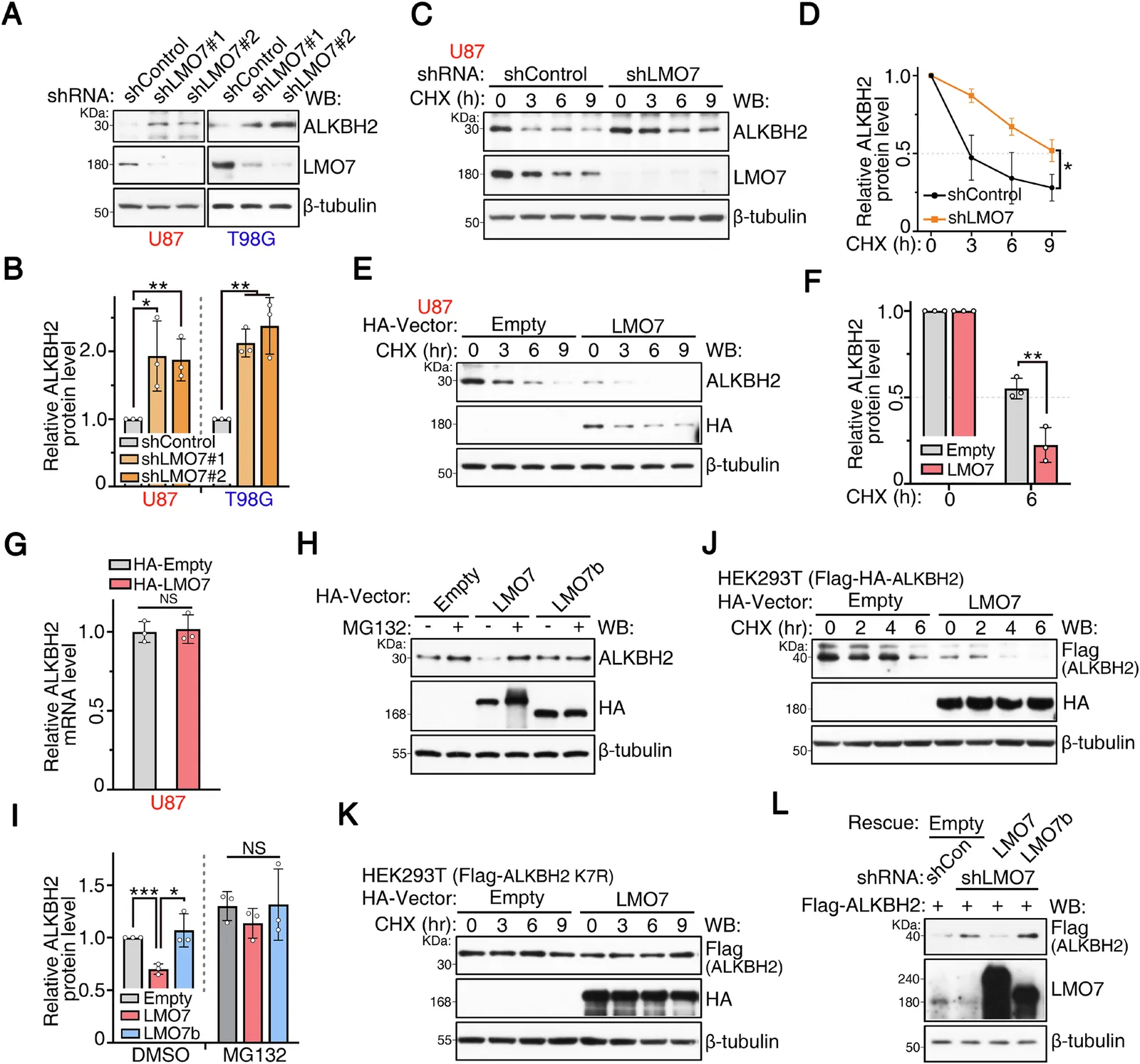
LMO7 promotes proteasome-dependent degradation of ALKBH2. **A** U87 and T98G cells were transduced with control shRNA (shCon) or two independent shRNAs targeting LMO7, and ALKBH2 and LMO7 protein levels were analyzed by WB. **B** Quantification of ALKBH2 protein levels from (**A**) in U87 and T98G cells. C U87 cells expressing shCon or shLMO7 were treated with cycloheximide (CHX, 150 μg/mL) for the indicated times, and ALKBH2 protein levels were analyzed by WB. **D** Quantification of ALKBH2 protein levels from (**C**). **E** U87 cells expressing empty HA vector or HA-LMO7 were treated with CHX for the indicated times, and ALKBH2 protein levels were analyzed by WB. **F** Quantification of ALKBH2 protein levels at 0 and 6 h from (**E**). **G** U87 cells were transfected with empty HA vector or HA-LMO7, and *ALKBH2* mRNA levels were measured by reverse transcription quantitative PCR (RT-qPCR). **H** U87 cells expressing empty HA vector, HA-LMO7, or HA-LMO7b were treated with or without MG132, and ALKBH2 protein levels were analyzed by WB. **I** Quantification of ALKBH2 protein levels from (**H**). **J** HEK293T cells stably expressing Flag-HA-ALKBH2 were transfected with empty HA vector or HA-LMO7, treated with CHX for the indicated times, and analyzed by WB. **K** HEK293T cells stably expressing Flag-ALKBH2 K7R were transfected with empty HA vector or HA-LMO7, treated with CHX for the indicated times, and analyzed by WB. **L** Rescue experiment in HEK293T cells stably expressing Flag-ALKBH2. Endogenous LMO7 was depleted by shRNA and reconstituted with empty vector, LMO7, or LMO7b, followed by WB analysis. Data are presented as mean ± SD. Statistical significance was determined using unpaired two-tailed Student’s *t*-tests. *p < 0.05, **p < 0.01, ***p < 0.001; NS not significant.

To determine whether LMO7 regulates ALKBH2 protein levels, we depleted LMO7 in U87 and T98G glioma cells using two independent shRNAs. Both shRNAs increased ALKBH2 protein levels in U87 and T98G cells, and quantification confirmed this increase in both cell lines (Fig. 5A, B). CHX chase assays in U87 cells showed that ALKBH2 protein levels declined after CHX treatment in control cells, whereas LMO7 depletion delayed ALKBH2 degradation (Fig. 5C, D). Conversely, LMO7 overexpression reduced ALKBH2 protein levels and accelerated ALKBH2 degradation in U87 cells without altering *ALKBH2* mRNA levels (Fig. 5E–G).

We next tested whether proteasome inhibition blocks the LMO7-dependent decrease in ALKBH2 protein levels. In U87 cells, MG132 restored ALKBH2 protein levels in cells expressing LMO7 or LMO7b and minimized differences among groups, indicating that LMO7-dependent reduction of ALKBH2 requires proteasome activity (Fig. 5H, I).

To test whether LMO7 also regulates exogenous ALKBH2 turnover, we used HEK293T cells stably expressing Flag-ALKBH2. LMO7 overexpression accelerated the degradation of Flag-ALKBH2 after CHX treatment (Fig. 5J). In the same assay, the K7R mutant showed delayed degradation compared with WT ALKBH2, and the destabilizing effect of LMO7 was attenuated in the K7R mutant (Fig. 5K). In rescue experiments, re-expression of full-length LMO7 in LMO7-depleted cells reduced Flag-ALKBH2 protein levels toward control levels, whereas LMO7b produced a weaker effect under the same conditions (Fig. 5L). Collectively, these data indicate that LMO7 promotes proteasome-dependent degradation of ALKBH2 and that Lys7 contributes to this turnover.

### Oxidative stress promotes LMO7- and Lys7-dependent polyubiquitination and degradation of ALKBH2

**Fig. 6:**
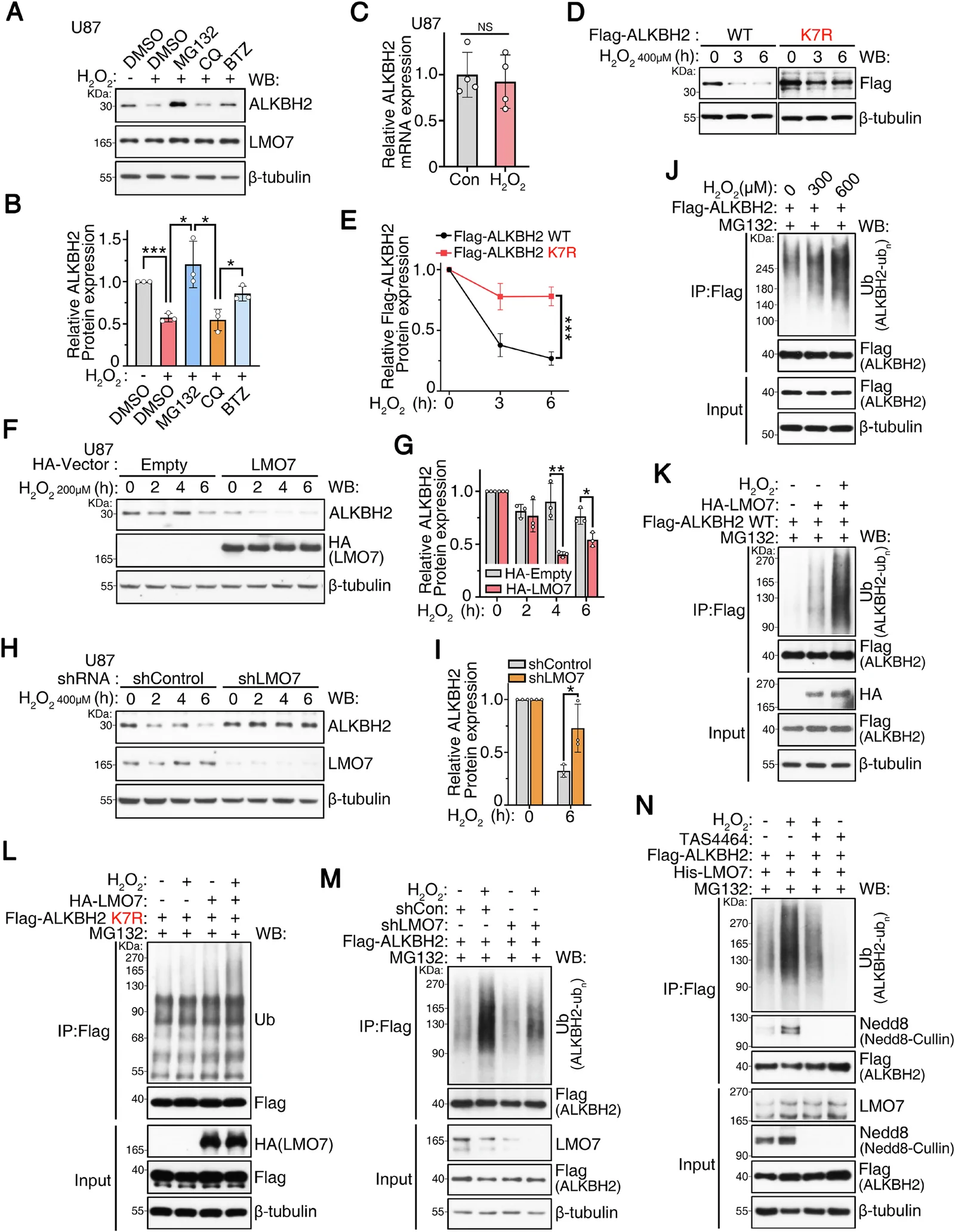
Oxidative stress promotes LMO7- and Lys7-dependent polyubiquitination and degradation of ALKBH2. **A** U87 cells were treated with hydrogen peroxide (H_2_O_2_) in the presence of DMSO, MG132, bortezomib (BTZ), or chloroquine (CQ), and ALKBH2 and LMO7 protein levels were analyzed by WB. **B** Quantification of relative ALKBH2 protein levels from (**A**). **C** RT-qPCR analysis of *ALKBH2* mRNA levels in U87 cells treated with H_2_O_2_ for 6 h. **D** U87 cells stably expressing Flag-ALKBH2 WT or K7R were treated with H_2_O_2_ for the indicated times, and Flag-ALKBH2 protein levels were analyzed by WB. **E** Quantification of relative Flag-ALKBH2 protein levels from (**D**). **F** U87 cells expressing empty HA vector or HA-LMO7 were treated with H_2_O_2_ for the indicated times, and ALKBH2 protein levels were analyzed by WB. **G** Quantification of relative ALKBH2 protein levels from (**F**). **H** U87 cells expressing shCon or shLMO7 were treated with H_2_O_2_ for the indicated times, and ALKBH2 protein levels were analyzed by WB. **I** Quantification of relative ALKBH2 protein levels from (**H**). **J** HEK293T cells stably expressing Flag-ALKBH2 were treated with increasing concentrations of H_2_O_2_ for 6 h in the presence of MG132. Flag IP was followed by WB to analyze ALKBH2 polyubiquitination. **K** HEK293T cells stably expressing Flag-ALKBH2 were transfected with empty HA vector or HA-LMO7 and treated with or without H_2_O_2_ for 6 h in the presence of MG132. Flag IP was followed by WB to analyze ALKBH2 polyubiquitination. **L** Cells stably expressing Flag-ALKBH2 K7R were transfected with empty HA vector or HA-LMO7 and treated with or without H_2_O_2_ for 6 h in the presence of MG132. Flag IP was followed by WB to analyze ALKBH2 polyubiquitination. **M** HEK293T cells stably expressing Flag-ALKBH2 were transduced with shCon or shLMO7 and treated with or without H_2_O_2_ for 6 h in the presence of MG132. Flag IP was followed by WB to analyze ALKBH2 polyubiquitination. **N** HEK293T cells expressing Flag-ALKBH2 and His-LMO7 were treated with H_2_O_2_ in the presence or absence of TAS4464 and MG132. Flag IP was followed by WB to analyze ALKBH2 polyubiquitination and cullin neddylation. Data are presented as mean ± SD. Statistical significance was determined using unpaired two-tailed Student’s *t*-tests. *p < 0.05, **p < 0.01, ***p < 0.001; NS not significant.

To determine whether oxidative stress modulates ALKBH2 protein levels, U87 cells were exposed to hydrogen peroxide (H_2_O_2_) in the presence of proteasome or lysosomal inhibitors. H_2_O_2_ reduced ALKBH2 protein levels, and this reduction was attenuated by MG132 and bortezomib, whereas CQ had no comparable effect (Fig. 6A, B). *ALKBH2* mRNA levels remained unchanged after H_2_O_2_ exposure (Fig. 6C), consistent with regulation at the protein level. In U87 cells stably expressing Flag-ALKBH2 WT or K7R, H_2_O_2_ induced a time-dependent decrease in WT ALKBH2, whereas the K7R substitution markedly delayed H_2_O_2_-induced ALKBH2 degradation (Fig. 6D, E). LMO7 overexpression enhanced the H_2_O_2_-associated reduction in ALKBH2 protein levels, whereas LMO7 depletion attenuated this reduction (Fig. 6F–I).

We next examined whether H_2_O_2_ promotes ALKBH2 ubiquitination. In HEK293T cells stably expressing Flag-ALKBH2 WT, H_2_O_2_ increased ALKBH2 polyubiquitination in a dose-dependent manner (Fig. 6J), and co-expression of LMO7 further enhanced this response (Fig. 6K). Compared with WT ALKBH2, K7R showed a near-complete loss of H_2_O_2_-induced polyubiquitination and a reduced, but not eliminated, response to combined LMO7 overexpression and H_2_O_2_ treatment (Fig. 6K, L). To further test LMO7 dependence, LMO7 depletion reduced H_2_O_2_-induced ALKBH2 polyubiquitination (Fig. 6M).

SCF complex activity depends on NEDD8-mediated cullin neddylation [39, 40]. To test whether H_2_O_2_-induced ALKBH2 ubiquitination requires cullin neddylation, cells were treated with the NEDD8-activating enzyme inhibitor TAS4464 [41]. H_2_O_2_ increased cullin neddylation, as indicated by elevated NEDD8–cullin conjugates, whereas TAS4464 suppressed cullin neddylation and reduced H_2_O_2_-induced ALKBH2 polyubiquitination in LMO7-overexpressing cells (Fig. 6N). Collectively, these data indicate that oxidative stress promotes LMO7-dependent ALKBH2 polyubiquitination and proteasomal degradation through NEDD8-dependent cullin neddylation, with Lys7 serving as a major ubiquitin acceptor site.

### LMO7 is reduced in glioblastoma and promotes TMZ sensitivity through ALKBH2 degradation

**Fig. 7:**
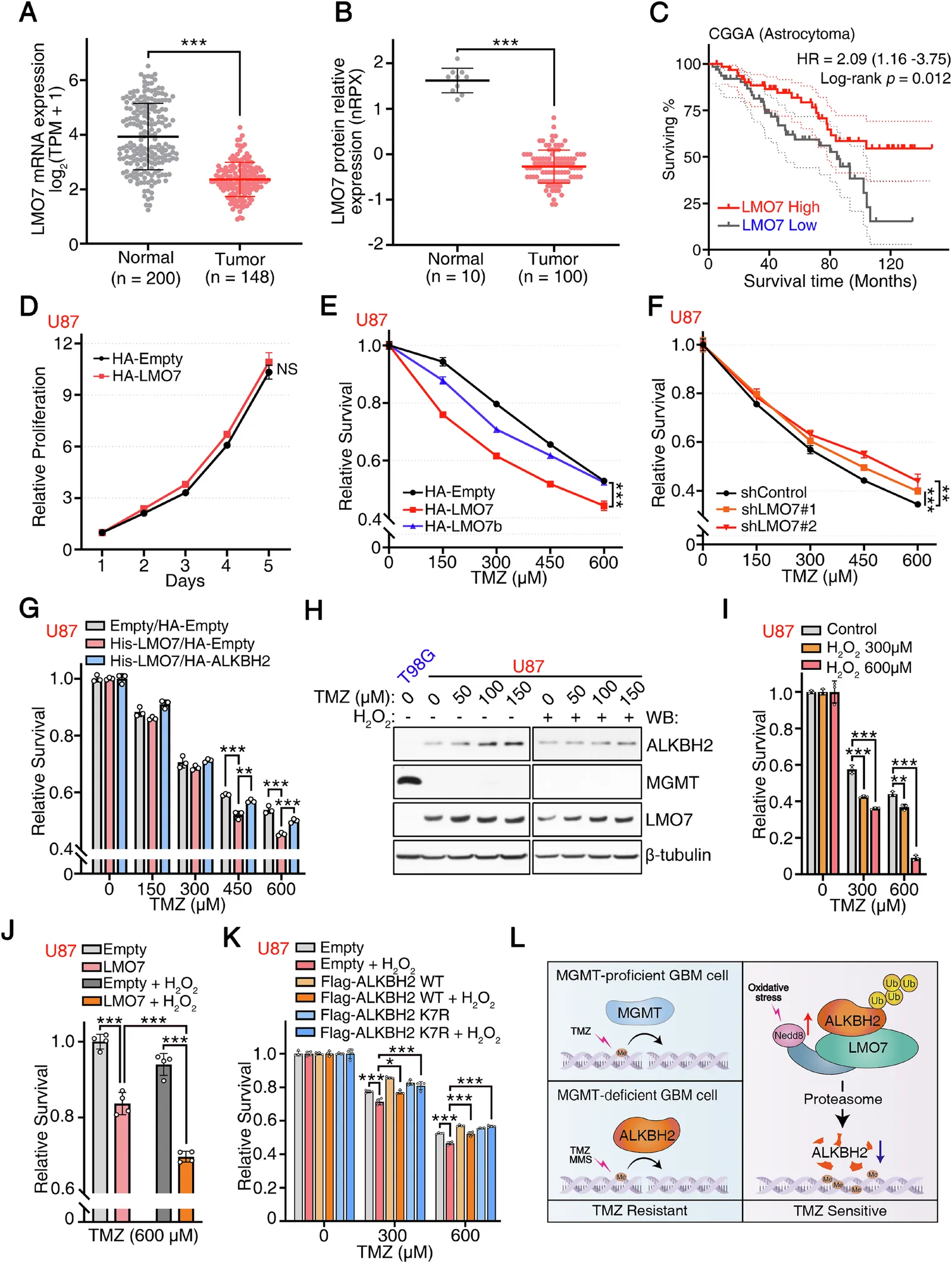
LMO7 is reduced in glioblastoma and promotes TMZ sensitivity through ALKBH2 degradation. **A** *LMO7* mRNA expression in GBM and normal brain tissues from TCGA and GTEx datasets, shown as log2(TPM + 1). **B** LMO7 protein levels in normal brain tissues (*n* = 10) and GBM samples (*n* = 100) from the CPTAC dataset, shown as normalized relative protein expression (nRPX). **C** Kaplan–Meier survival analysis of astrocytoma patients stratified by high or low *LMO7* expression in the CGGA dataset. **D** Proliferation of U87 cells expressing empty HA vector or HA-LMO7 was assessed by CCK-8 assay. **E** Relative survival of U87 cells expressing empty HA vector, HA-LMO7, or HA-LMO7b after TMZ treatment for 48 h was measured by CCK-8 assay. **F** Relative survival of U87 cells expressing shCon or two independent shRNAs targeting LMO7 after TMZ treatment for 48 h was measured by CCK-8 assay. **G** Relative survival of U87 cells co-expressing His-LMO7 with empty HA vector or HA-ALKBH2 after TMZ treatment for 48 h was measured by CCK-8 assay. **H** T98G and U87 cells were treated with TMZ in the presence or absence of H_2_O_2_, and ALKBH2, MGMT, and LMO7 protein levels were analyzed by WB. **I** Relative survival of U87 cells treated with TMZ in the presence or absence of H_2_O_2_ was measured by CCK-8 assay. **J** Relative survival of U87 cells expressing empty vector or LMO7 after TMZ treatment in the presence or absence of H_2_O_2_ was measured by CCK-8 assay. **K** Relative survival of MGMT-deficient U87 cells expressing empty vector, Flag-ALKBH2 WT, or Flag-ALKBH2 K7R after TMZ treatment in the presence or absence of H_2_O_2_ was measured by CCK-8 assay. **L** Working model. In MGMT-proficient GBM cells, MGMT repairs TMZ-induced methyl adducts and promotes TMZ resistance. In MGMT-deficient GBM cells, ALKBH2 provides MGMT-independent alkylation repair capacity and sustains TMZ resistance. Oxidative stress enhances cullin neddylation and promotes LMO7-dependent K48-linked polyubiquitination and proteasomal degradation of ALKBH2, thereby reducing ALKBH2-mediated repair capacity and increasing TMZ sensitivity. Data are presented as mean ± SD. Statistical significance was assessed using a log-rank test in (C) and unpaired two-tailed Student’s *t*-tests for quantitative comparisons. *p < 0.05, **p < 0.01, ***p < 0.001; NS not significant.

To assess the clinical relevance of LMO7 in glioma, we analyzed publicly available datasets. TCGA and Genotype-Tissue Expression (GTEx) analyses showed lower *LMO7* mRNA expression in GBM than in normal brain (Fig. 7A), and CPTAC proteomic data similarly showed reduced LMO7 protein levels in tumors (Fig. 7B). In the CGGA astrocytoma cohort, lower *LMO7* expression was associated with shorter overall survival (Fig. 7C).

We next tested whether LMO7 alters TMZ sensitivity in U87 GBM cells. LMO7 overexpression did not change basal proliferation (Fig. 7D). Under TMZ treatment, LMO7 overexpression increased TMZ sensitivity, whereas LMO7b did not produce a comparable effect (Fig. 7E). Conversely, LMO7 depletion reduced TMZ sensitivity (Fig. 7F). ALKBH2 overexpression attenuated the TMZ-sensitizing effect of LMO7, indicating that ALKBH2 contributes to the LMO7-dependent response (Fig. 7G).

To determine whether Lys7-dependent ALKBH2 degradation contributes to this response, we analyzed U87 cells expressing Flag-ALKBH2 WT or K7R. LMO7 increased TMZ sensitivity in cells expressing WT ALKBH2, whereas this effect was reduced in cells expressing ALKBH2 K7R (Supplementary Fig. 3A, B). Unlike full-length LMO7, LMO7b did not increase TMZ sensitivity in cells expressing ALKBH2 K7R (Supplementary Fig. 3C, D). Conversely, LMO7 depletion reduced TMZ sensitivity in cells expressing WT ALKBH2, whereas this effect was attenuated in cells expressing ALKBH2 K7R (Supplementary Fig. 3E, F). These data indicate that Lys7-dependent ALKBH2 degradation contributes to LMO7-dependent modulation of TMZ sensitivity.

We then asked whether oxidative stress alters TMZ-associated ALKBH2 accumulation and TMZ sensitivity. In U87 cells, TMZ increased ALKBH2 protein levels across the tested dose range, whereas H_2_O_2_ co-treatment attenuated this accumulation (Fig. 7H). H_2_O_2_ reduced cell viability during TMZ exposure, and LMO7 overexpression further reduced viability during combined TMZ and H_2_O_2_ treatment (Fig. 7I, J). In U87 cells expressing Flag-ALKBH2 WT or K7R, both WT and K7R ALKBH2 increased cell survival during TMZ exposure, whereas the K7R substitution attenuated the H_2_O_2_-induced reduction in the ALKBH2-mediated increase in cell survival (Fig. 7K). Because direct H_2_O_2_ addition has limited translational relevance, we tested ferric ammonium citrate (FAC), an iron-loading reagent that can increase intracellular reactive oxygen species via iron-dependent Fenton chemistry [42]. Similar to H_2_O_2_, FAC co-treatment reduced U87 cell viability after MMS or TMZ exposure (Supplementary Fig. 3G). Collectively, these data indicate that oxidative stress increases TMZ sensitivity in MGMT-deficient U87 cells by limiting ALKBH2-mediated protection, at least in part through LMO7-dependent degradation of ALKBH2.

## Discussion

Here, we identify an oxidative stress-responsive LMO7–ALKBH2 ubiquitination pathway that regulates ALKBH2 protein stability and modulates TMZ sensitivity in GBM, with particular relevance to MGMT-deficient tumors. Proteomic analyses of glioma cohorts show elevated ALKBH2 protein levels and an inverse association between ALKBH2 and MGMT protein levels (Fig. 1). In MGMT-deficient U87 cells, ALKBH2 depletion increases sensitivity to alkylating agents, whereas ALKBH2 re-expression restores alkylator tolerance (Fig. 2). LMO7 binds ALKBH2 and promotes K48-linked polyubiquitination of ALKBH2 at Lys7, leading to proteasome-dependent degradation, and oxidative stress augments this process via NEDD8-dependent cullin neddylation (Figs. 3–7). The K7R substitution attenuates LMO7- and H_2_O_2_-induced ALKBH2 degradation and reduces the TMZ-sensitizing effect of this pathway. Collectively, these results indicate that oxidative stress enhances LMO7-dependent ubiquitination of ALKBH2, lowers ALKBH2 protein levels, and increases alkylator sensitivity when MGMT-dependent repair is limited (Fig. 7L).

A key mechanistic insight from this study is that the E3 ligase LMO7 promotes K48-linked polyubiquitination and proteasomal degradation of ALKBH2, thereby modulating alkylation damage tolerance and TMZ sensitivity. AlkB-family enzymes function broadly in nucleic acid repair [43], whereas ubiquitin-dependent regulation has been documented mainly in RNA-modifying family members, including ALKBH5 and FTO [44, 45]. In GBM, the E3 ligases and deubiquitinases that control ALKBH2 remain incompletely characterized. This study places ALKBH2 within an LMO7-dependent E3 ubiquitin axis and links this pathway to TMZ sensitivity (Fig. 7 and Supplementary Fig. 3). The K7R substitution prolonged ALKBH2 stability, reduced K48-linked polyubiquitination, and attenuated LMO7-dependent ALKBH2 degradation, indicating that Lys7 is a major ubiquitin acceptor site, although residual ubiquitination suggests that additional sites may contribute. Notably, a recent preprint from our group reported TRIM28-associated proteasomal degradation of ALKBH2 in non-small cell lung cancer [37]. Together, these studies indicate that ALKBH2 stability is controlled by distinct E3-dependent ubiquitin pathways across tumor contexts. Because N-alkyl DNA lesions are processed through BER and AlkB-family oxidative demethylation pathways [11, 15, 17, 20, 46], LMO7-driven ALKBH2 degradation may reduce MGMT-independent repair capacity during TMZ exposure.

This study links oxidative stress to an LMO7-dependent ubiquitin pathway through a neddylation-dependent mechanism that increases LMO7-driven K48-linked polyubiquitination and proteasomal degradation of ALKBH2. LMO7 has been reported to act as an F-box substrate adaptor in an SCF (Skp1-Cullin-F-box) E3 ubiquitin ligase complex [47, 48], and this ligase activity requires NEDD8-mediated cullin neddylation [49–51]. In line with this requirement, H_2_O_2_ increased cullin neddylation and enhanced LMO7-dependent K48-linked polyubiquitination of ALKBH2, whereas TAS4464 suppressed this effect (Fig. 6). These data indicate that an LMO7-containing SCF complex contributes to ALKBH2 ubiquitination under H_2_O_2_-induced oxidative stress. This pathway is not proposed to depend on MGMT or *O*^6^-methylguanine, but its effect on TMZ response is most evident when MGMT-dependent repair is limited. Given reports that TMZ can induce ROS [52], we asked whether TMZ exposure was sufficient to activate the neddylation-dependent LMO7–ALKBH2 degradation pathway. Under the tested conditions, TMZ alone did not robustly activate this pathway, whereas H_2_O_2_ reduced ALKBH2 protein levels and increased TMZ sensitivity (Fig. 7F, H–J). These high-dose, short-term TMZ assays therefore primarily assess N-alkyl lesion-associated tolerance and do not directly model clinical TMZ exposure [53, 54].

Clinically, these data link LMO7-dependent control of ALKBH2 protein levels to variable TMZ responses in MGMT-deficient GBM. MGMT promoter methylation and low MGMT protein level are widely used to predict TMZ sensitivity [4, 55], but a substantial subset of MGMT-low tumors derives limited benefit [6, 55], implicating MGMT-independent repair and damage-tolerance programs that blunt alkylator efficacy [15, 56]. These programs may include base excision repair, homologous recombination, and TMZ-induced cellular senescence, although senescence was not examined in our ALKBH2-centered assays [57]. In this setting, ALKBH2 protein levels are higher in GBM than in normal brain tissue (Fig. 1C, D), and higher *ALKBH2* expression is associated with shorter overall survival in IDH-wild-type glioma patients recorded as having received chemotherapy (Fig. 2C). Patient proteomic datasets show an inverse correlation between LMO7 and ALKBH2 protein levels (Supplementary Fig. 2B), and lower *LMO7* expression is associated with shorter overall survival (Fig. 7C), consistent with reduced ALKBH2 clearance in LMO7-low tumors. Together, these clinical observations are consistent with an LMO7-dependent ubiquitin pathway that regulates ALKBH2 protein levels and influences TMZ response in MGMT-deficient GBM models. Although enhancing LMO7–CRL/SCF activity or inhibiting ALKBH2 may reduce ALKBH2-dependent repair capacity, any therapeutic application of these approaches would require evaluation of blood-brain barrier penetration, tumor selectivity, and CNS toxicity. In addition, LMO7 protein levels may help stratify MGMT-deficient tumors as a candidate marker associated with ALKBH2 clearance and TMZ response, to be evaluated alongside MGMT promoter methylation and IDH status in future cohorts.

In summary, this study characterizes an oxidative stress-responsive LMO7–ALKBH2 ubiquitination pathway that promotes K48-linked polyubiquitination and proteasomal degradation of ALKBH2, thereby limiting MGMT-independent alkylation damage tolerance and increasing TMZ sensitivity in GBM, with particular relevance to MGMT-deficient tumors. Mechanistically, oxidative stress increases cullin neddylation and augments LMO7-dependent ubiquitination of ALKBH2. This work relies primarily on cellular models and public datasets, and the extent to which this mechanism operates across GBM subtypes and within the in vivo microenvironment remains to be determined. Further studies using clinically relevant TMZ exposure schedules, patient-derived GBM models, and orthotopic or genetically engineered models are needed to determine whether modulating the LMO7–ALKBH2 pathway can increase TMZ sensitivity in MGMT-deficient GBM.

## Materials and methods

### Cell culture

U87, T98G, U251, and HEK293T cells were obtained from the American Type Culture Collection (ATCC, USA) and cultured in Dulbecco’s Modified Eagle Medium (DMEM, 11965092, Gibco, USA) supplemented with 10% fetal bovine serum (FBS, 10270-106, Gibco, USA) and 1% penicillin-streptomycin (Gibco, USA; 100 U/mL penicillin and 100 µg/mL streptomycin). Cells were maintained at 37 °C in a humidified atmosphere containing 5% CO₂. Mycoplasma contamination was routinely monitored using a luminescence-based detection assay (C0297, Beyotime, China).

### Antibodies and reagents

The following primary antibodies were used: anti-ALKBH2 (A8228, MilliporeSigma, USA), anti-ALKBH3 (09-882, MilliporeSigma, USA), anti-MGMT (17195-1-AP, Proteintech, China), anti-LMO7 (ab224113, Abcam, UK), anti-Flag (F3165, MilliporeSigma, USA), anti-HA (51064-2-AP, Proteintech, China), anti-NEDD8 (P80240, MCE, USA), anti-ubiquitin (sc-8017, Santa Cruz, USA), anti-ubiquitin, Lys48-specific, recognizing K48-linked polyubiquitin chains (05-1307, MilliporeSigma, USA), and anti-histone H3 (17168-1-AP, Proteintech, China). Horseradish peroxidase (HRP)-conjugated anti-β-tubulin (E021043, EarthOx, USA) was used as a loading control. HRP-conjugated secondary antibodies included anti-rabbit IgG (SA00001-2, Proteintech, China) and anti-mouse IgG (SA00001-1, Proteintech, China).

Temozolomide (TMZ, S1237, Selleck, USA), cycloheximide (CHX, 2112S, CST, USA), methyl methanesulfonate (MMS, 129925, MilliporeSigma, USA), the proteasome inhibitors MG132 (S2619, Selleck, USA) and bortezomib (BTZ, HY10227, MCE, USA), the lysosomal inhibitor chloroquine (CQ, HY17589A, MCE, USA), hydrogen peroxide (H_2_O_2_, 800709938, HuShi, China), ferric ammonium citrate (FAC, A100170, Aladdin, China), the NEDD8-activating enzyme inhibitor TAS4464 (HY128586, MCE, USA), and the Cell Counting Kit-8 (CCK-8) assay kit (K1018, ApexBio, USA) were used as indicated. The protease and phosphatase inhibitor cocktail was purchased from Beyotime (P1051, China).

### Immunoprecipitation (IP) and western blotting (WB)

IP assays were performed as previously described [29]. HEK293T cells were transfected with the indicated expression plasmids using polyethylenimine (PEI) and cultured for 48–72 h. Cells were harvested and lysed in lysis buffer supplemented with protease and phosphatase inhibitors. Lysates were clarified by centrifugation and incubated overnight at 4 °C with anti-Flag M2-agarose beads (A2220, MilliporeSigma, USA) or anti-HA-agarose beads (B26201, Selleck, USA) with gentle rotation. After washing with wash buffer, bound proteins were eluted by heating in Laemmli sample buffer and analyzed by WB.

For WB, protein samples were resolved by SDS–PAGE and transferred onto polyvinylidene difluoride (PVDF) membranes (MilliporeSigma, USA). Membranes were blocked and incubated with primary antibodies, followed by horseradish peroxidase (HRP)-conjugated secondary antibodies. Protein bands were detected using enhanced chemiluminescence (ECL).

### Plasmid constructs, mutagenesis, and lentiviral infection

Full-length human ALKBH2 cDNA was used as the template to generate ALKBH2 K7R and K16R mutants by PCR-based site-directed mutagenesis. Human LMO7 cDNA (NM_001306080.2) was obtained from Youbio, China. LMO7 deletion mutants, including LMO7b, ΔPDZ, and ΔLIM, were generated by PCR, cloned into the pENTR-4 vector, and transferred into the pHAGE-CMV-Flag/HA/His destination vector by Gateway LR recombination (11791020, Invitrogen, USA). ALKBH2 variants were cloned into pCMV-Flag for mammalian expression and pET28a-His-2×Flag for bacterial expression. All constructs were confirmed by sequencing. Transient transfections were performed using PEI (K2014, MCE, USA).

For short hairpin RNA (shRNA)-mediated knockdown, annealed oligonucleotides targeting the indicated sequences were cloned into the pLKO.1 vector (8453, Addgene, USA), and a non-targeting shRNA was used as the control. Lentiviruses were produced in HEK293T cells by co-transfecting lentiviral vectors (pLKO.1 or pHAGE) with packaging plasmids psPAX2 and pMD2.G. Viral supernatants were collected 48–72 h post-transfection, clarified, and filtered through 0.45-μm membranes. Target cells were infected in the presence of 6 µg/mL polybrene for 12 h. Stable cell lines were selected with puromycin (1 µg/mL) or blasticidin (5 µg/mL) for 3 days and maintained in selection medium. Knockdown efficiency was confirmed by WB. The target sequences for shRNA were as follows: shALKBH2#1, 5′-GTG CTC ATC AAC AGG TAT AAA-3′; shALKBH2#2, 5′-CCA CGG GAG CTT ACT AAT GAT-3′; shALKBH3, 5′-AAT GCG GTT CTT TAG TGT GCG-3′; shLMO7#1, 5′-AAA TGA TAA CAA AGA CCC AGG-3′; shLMO7#2, 5′-TTC TAT GTA ATC TAA AGA GGC-3′.

### Maltose-binding protein (MBP) pull-down assay

MBP-tagged LMO7, MBP control, and Flag-tagged ALKBH2 were expressed in *E. coli* Rosetta (DE3) cells induced with 0.7 mM isopropyl β-D-1-thiogalactopyranoside (IPTG) at 16 °C overnight. Cell lysates containing MBP-fusion proteins were immobilized on amylose resin (E8021S, NEB, USA) overnight at 4 °C. After three washes, the resin was incubated with Flag-ALKBH2 lysates at 4 °C for 4 h. Bound proteins were washed, eluted in Laemmli sample buffer, and analyzed by WB.

### Affinity purification of K48-linked polyubiquitinated proteins

K48-linked polyubiquitinated proteins were isolated from cell extracts using HaloTag tandem ubiquitin-binding entity (Halo-TUBE) resin under denaturing conditions. Briefly, cells were harvested, lysed, and denatured by boiling in TBS containing 1% SDS, then diluted with lysis buffer to reduce the SDS concentration to 0.1% before affinity purification. Extracts were incubated with 10 μL of HaloLink™ resin (G1915, Promega, USA) pre-bound to purified Halo-TUBE protein (10 μg per sample). The clarified extracts were incubated with Halo-TUBE-bound resin overnight at 4 °C with rotation. Subsequently, the resin was washed five times with washing buffer, boiled in Laemmli sample buffer, and analyzed by WB.

### RNA isolation and RT-qPCR

Total RNA was isolated using the RNAeasy™ RNA Isolation Kit (R0026, Beyotime, China) following the manufacturer’s protocol. RNA concentration and purity were assessed using a NanoDrop spectrophotometer (Thermo Fisher Scientific, USA). First-strand cDNA was synthesized using the PrimeScript™ RT reagent Kit (RR047A, Takara, Japan), followed by quantitative reverse transcription PCR (RT-qPCR) analysis with SYBR Green qPCR Mix (TSE501, Tsingke, China). Gene expression levels were normalized to *β-actin*. The primer sequences were as follows: *β-actin* Forward: 5′-TCA TCA CTA TTG GCA ACG AGC GGT TC-3′, Reverse: 5′-TAC CAC CAG ACA GCA CTG TGT TGG CA-3′; *ALKBH2* Forward: 5′-GGG AGC ACC GAG ATG ATG AAA-3′, Reverse: 5′-CCT TAT GCC GGA AGA CAA AGT-3′.

### CHX chase assay

Cells were treated with 150 µg/mL CHX for the indicated durations to inhibit protein synthesis. At each time point, cells were harvested, and protein levels were analyzed by WB to assess protein stability.

### Cell viability and drug sensitivity assays

Cells were seeded into 96-well plates at a density of 2000–5000 cells per well. For TMZ and MMS sensitivity assays, cells were exposed to increasing concentrations of TMZ or MMS for 48 h. For oxidative stress combination assays, cells were treated with H_2_O_2_ or FAC alone or in combination with TMZ or MMS for 48 h. For rescue and mutant analyses, U87 cells expressing empty vector, Flag-ALKBH2 WT, or Flag-ALKBH2 K7R were treated with TMZ in the presence or absence of H_2_O_2_. In all experiments, cell viability was quantified using the CCK-8 assay.

### Bioinformatics and clinical data analysis

Bioinformatics and clinical analyses were performed using The Cancer Genome Atlas (TCGA) and Chinese Glioma Genome Atlas (CGGA) transcriptomic datasets, the Clinical Proteomic Tumor Analysis Consortium (CPTAC) proteomic data, and GlioVis survival resources, each accessed through its respective web portal. TCGA tumor–normal mRNA comparisons for *ALKBH2*, *ALKBH3*, *MGMT*, and *LMO7* were queried and visualized using OncoDB (https://oncodb.org/); CGGA mRNA and CPTAC protein analyses of the same genes were performed through the GlioVis and CPTAC portals, respectively.

In GlioVis, Kaplan–Meier curves were generated by stratifying patients into high- and low-expression groups based on the cohort median for each gene. For ALKBH2 survival analyses stratified by IDH status, GlioVis filters were set to Histology = All and Molecular subtype = Classical. For chemotherapy-status analyses, the cohort was restricted to IDH-wild-type glioma and further stratified according to the recorded CGGA/GlioVis Chemo_status field. Chemotherapy status was coded as received (Chemo_status = 1), not received (Chemo_status = 0), or unavailable (NA), and patients with unavailable status were excluded from chemotherapy-stratified survival analyses. Chemotherapy-stratified analyses were based on the recorded CGGA/GlioVis Chemo_status field rather than treatment-level clinical details. MGMT promoter methylation status was not used as a filtering variable for these analyses.

### Statistical analysis

Quantitative data are presented as mean ± standard deviation (SD) from at least three independent experiments (*n* ≥ 3). For multi-step assays with qualitative readouts, including WB, co-IP, and Halo-TUBE enrichment, experiments were independently repeated at least twice with similar results. Biological or technical replicates are indicated in the figure legends where applicable. Graphs and statistical analyses were generated using GraphPad Prism (version 9.5.0). Two-group comparisons were performed using unpaired two-tailed Student’s *t*-tests for normally distributed data or Mann–Whitney *U* tests for nonparametric data, as indicated in the figure legends. For dose-response and rescue assays, comparisons were performed at the indicated treatment concentrations. Correlations were assessed using the Pearson correlation coefficient (PCC). *p* < 0.05 was considered statistically significant, with *p < 0.05, **p < 0.01, ***p < 0.001.

## Supplementary information

Supplementary Figure and Legends

Uncropped WB files

## Data Availability

All data supporting this study are available within the article and its Supplementary Information.
