# Supplementary material for: E3 ligase LMO7 promotes ALKBH2 ubiquitination to sensitize MGMT-deficient glioblastoma to temozolomide
Source: Cell Death Dis. 2026 Jun 30;17(1):808. doi: 10.1038/s41419-026-09073-6 (PMC13586327; doi:10.1038/s41419-026-09073-6)
Supplement: Supplementary file 1 [file 41419_2026_9073_MOESM1_ESM.pdf]

SUPPLEMENTARY FIGURES

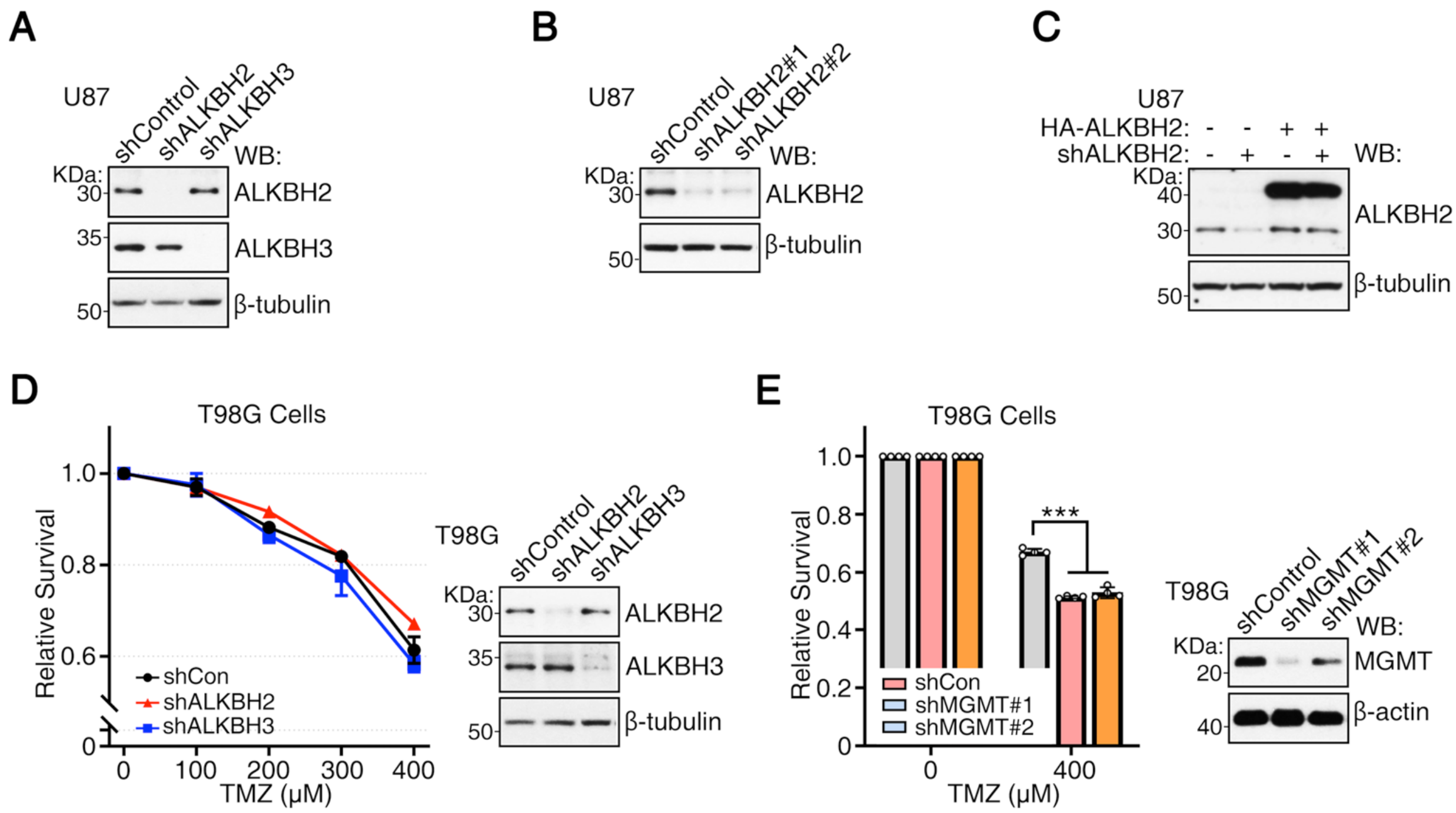

Supplementary Fig. 1 Validation and negative-control analyses for the ALKBH2–temozolomide (TMZ) response axis. (A) Western blotting (WB) validation of ALKBH2 and ALKBH3 knockdown in MGMT-deficient U87 cells used for the proliferation and TMZ sensitivity assays in Fig. 2E, F. **(B)** WB validation of ALKBH2 knockdown in MGMT-deficient U87 cells used for the methyl methanesulfonate (MMS) sensitivity assay in Fig. 2G. **(C)** WB validation of ALKBH2 depletion and HA-ALKBH2 reconstitution in MGMT-deficient U87 cells used for the rescue assays in Fig. 2H, I. **(D)** Relative survival of MGMT-proficient T98G glioma cells transduced with shCon, shALKBH2, or shALKBH3 following treatment with increasing concentrations of TMZ for 48 h, as measured by CCK-8 assay. WB validation of ALKBH2 and ALKBH3 depletion is shown on the right. **(E)** Parallel analysis of MGMT-proficient T98G cells transduced with shCon or two independent shMGMT constructs as a positive control. Cell viability was measured by CCK-8 assay, and WB validation of MGMT depletion is shown on the right. Error bars represent mean ± SD. Statistical significance was determined using an unpaired two-tailed Student's *t*-test, ***$p < 0.001$.

A

Gene Symbol	Unique	Total
URB5	43	50
RANBP2	39	43
LMO7	21	24
TRIM28	15	21

B C

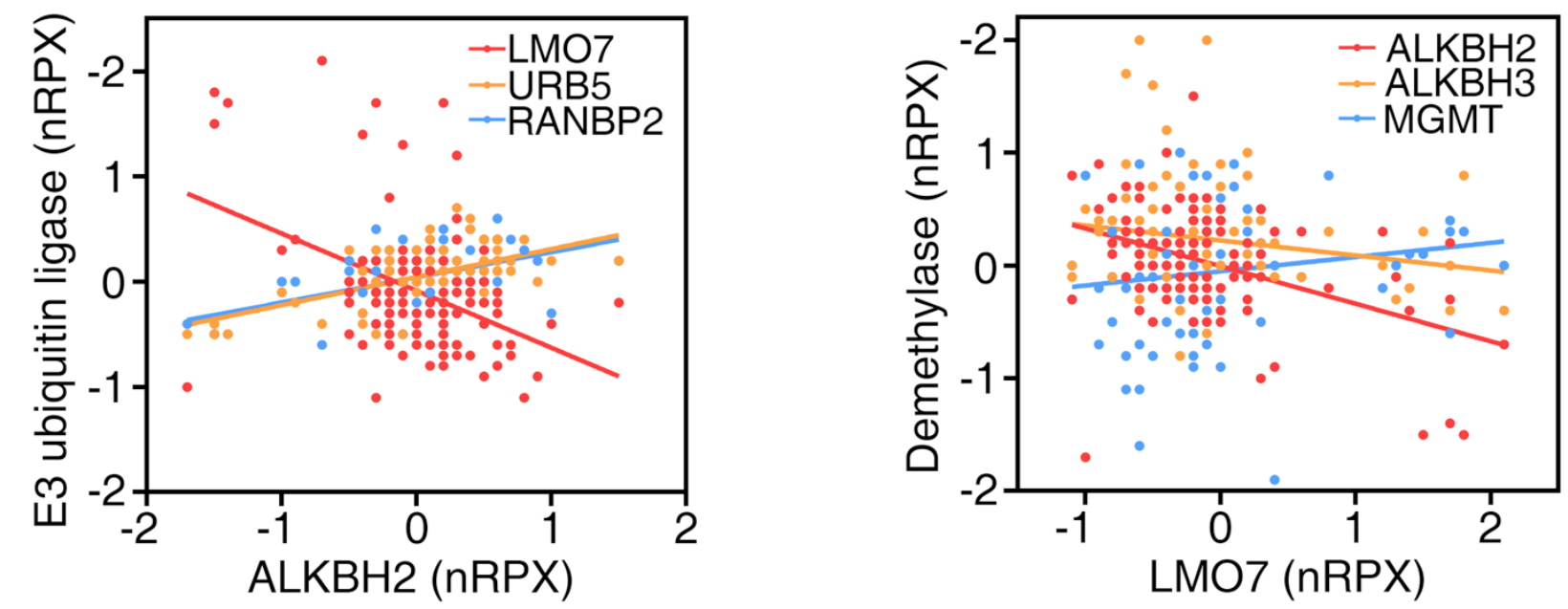

ALKBH2 Vs	R	P
LMO7	-0.425	<0.0001
URB5	0.527	<0.0001
RANBP2	0.471	<0.0001

LMO7 Vs	R	P
ALKBH2	-0.425	<0.0001
ALKBH3	-0.182	NS
MGMT	0.155	NS

Supplementary Fig. 2 Correlation analyses linking LMO7 with ALKBH2 among candidate E3 ligases. (A) Summary table of candidate E3 ligases obtained from a re-analysis of published proteomic datasets [1], showing the numbers of unique peptides and total peptides detected for each protein. **(B)** Correlation analysis between ALKBH2 protein levels and the indicated E3 ligases in the Clinical Proteomic Tumor Analysis Consortium (CPTAC) glioblastoma dataset, with values shown as normalized relative protein expression (nRPX). Pearson correlation coefficients (R) and *p* values are shown in the table. **(C)** Correlation analysis between LMO7 protein levels and ALKBH2, ALKBH3, or MGMT protein levels in the CPTAC glioblastoma dataset. Pearson correlation coefficients (R) and *p* values are shown in the table. NS, not significant.

1. Brickner JR, Soll JM, Lombardi PM, Vågbø CB, Mudge MC, Oyeniran C, et al. A ubiquitin-dependent signalling axis specific for ALKBH-mediated DNA dealkylation repair. Nature. 2017;551:389-393.

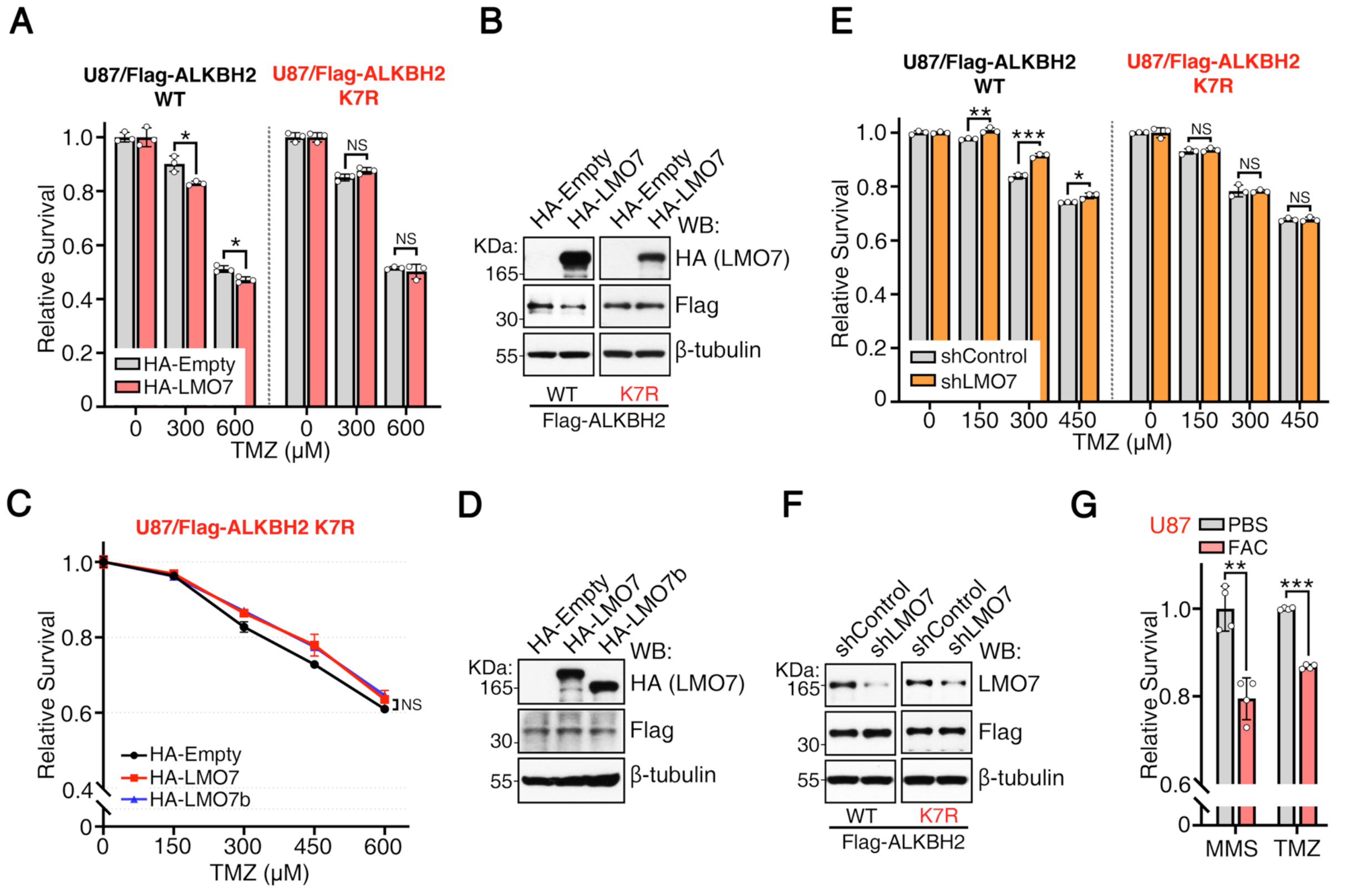

Supplementary Fig. 3 ALKBH2 Lys7 and ferric ammonium citrate treatment modulate alkylator sensitivity. **(A)** Relative survival of U87 cells stably expressing Flag-ALKBH2 wild-type (WT) or K7R and transfected with empty vector or HA-LMO7 f after 48 h TMZ treatment using CCK-8 assay. **(B)** WB analysis of Flag-ALKBH2 and LMO7 protein levels in U87 cells from panel **(A)**. **(C)** Relative survival of U87 cells stably expressing Flag-ALKBH2 K7R and transfected with empty vector, HA-LMO7, or HA-LMO7b after 48 h TMZ treatment using CCK-8 assay. **(D)** WB analysis of Flag-ALKBH2 K7R and LMO7 levels in U87 cells from panel **(C)**. **(E)** Relative survival of U87 cells stably expressing Flag-ALKBH2 WT or K7R and transduced with control shRNA or LMO7-specific shRNA after 48 h TMZ treatment using CCK-8 assay. **(F)** WB analysis of LMO7 levels in U87 cells from panel **(E)** to confirm knockdown efficiency. **(G)** Relative survival of U87 cells treated with MMS or TMZ, both at 450 μM, under iron-induced oxidative conditions generated by ferric ammonium citrate (FAC), as measured by CCK-8 assay. Data information: Error bars represent mean ± SD. Statistical significance was determined using unpaired two-tailed Student's *t*-tests, $*p < 0.05$, $**p < 0.01$, $***p < 0.001$. NS, not significant.
