# Supplementary material for: E3 ligase LMO7 promotes ALKBH2 ubiquitination to sensitize MGMT-deficient glioblastoma to temozolomide
Source: Cell Death Dis. 2026 Jun 30;17(1):808. doi: 10.1038/s41419-026-09073-6 (PMC13586327; doi:10.1038/s41419-026-09073-6)

Figure 1G

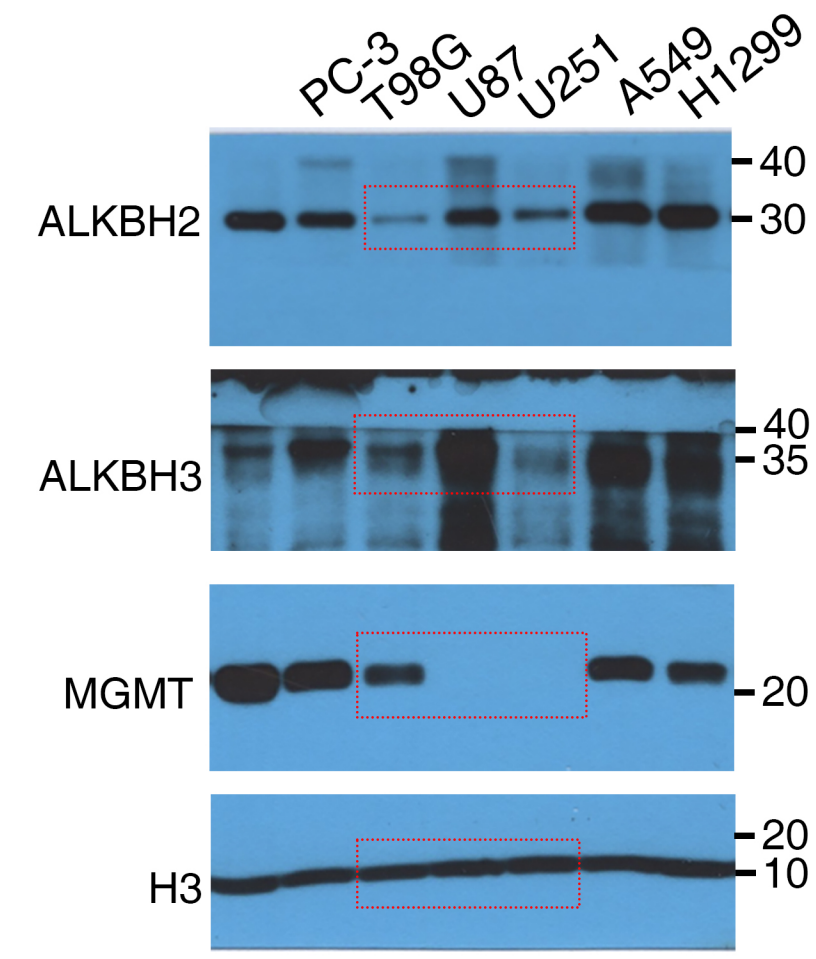

Figure 3A

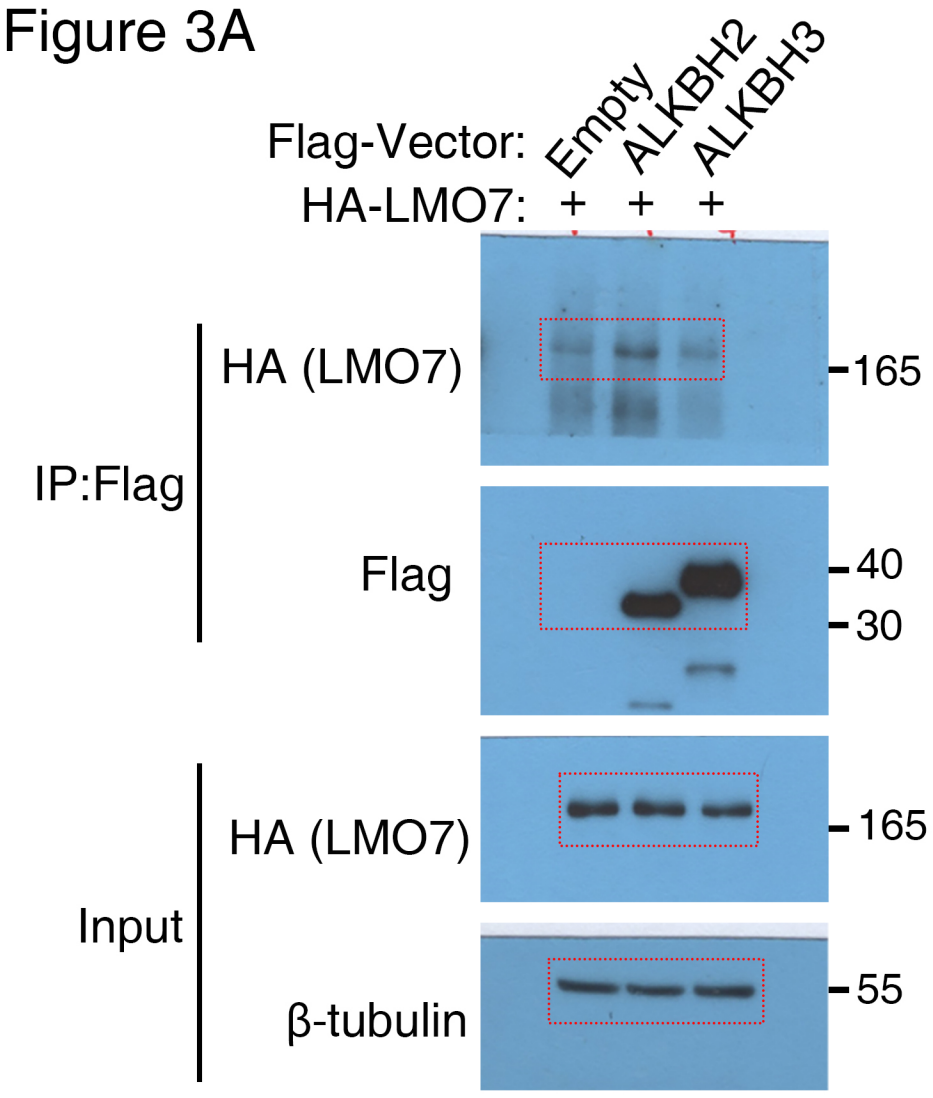

Figure 3B

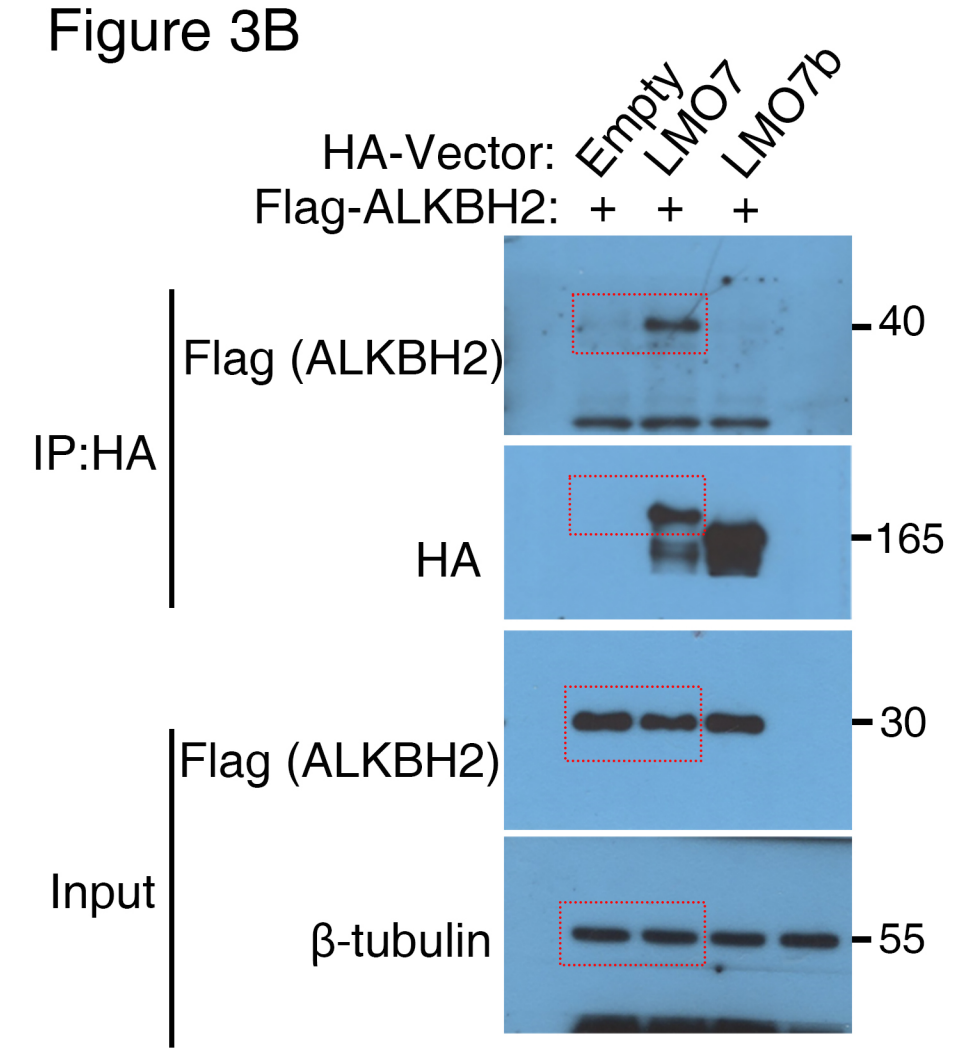

Figure 3C

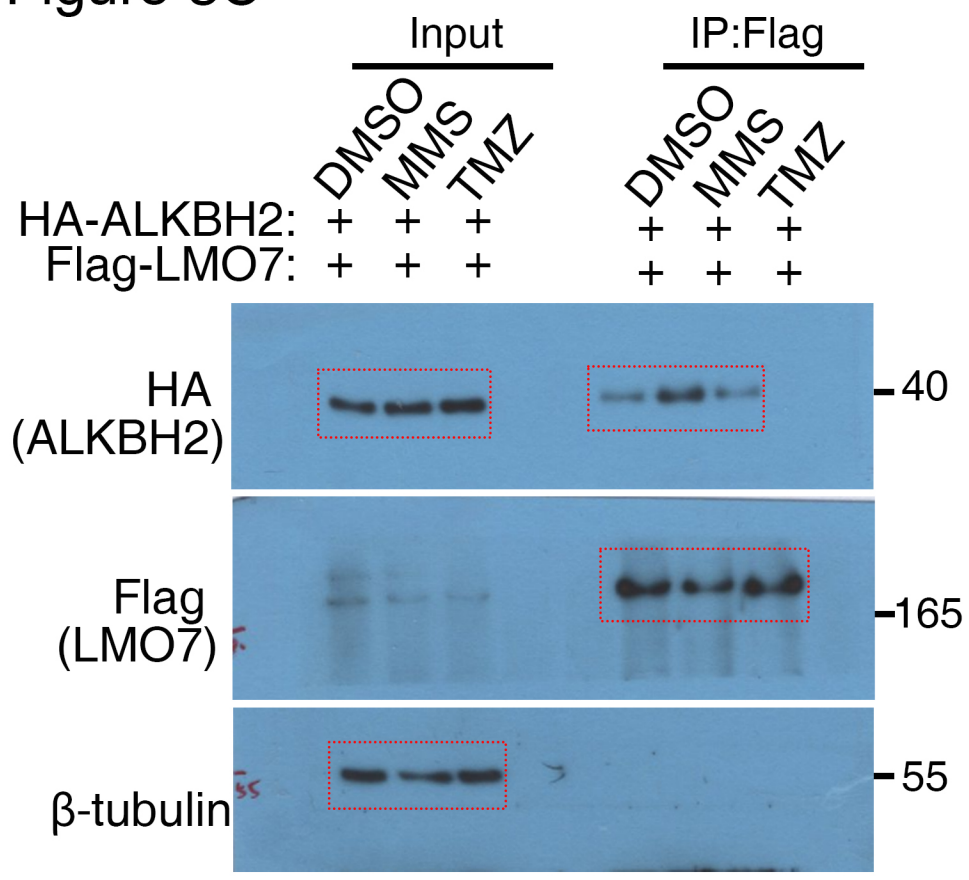

Figure 3D

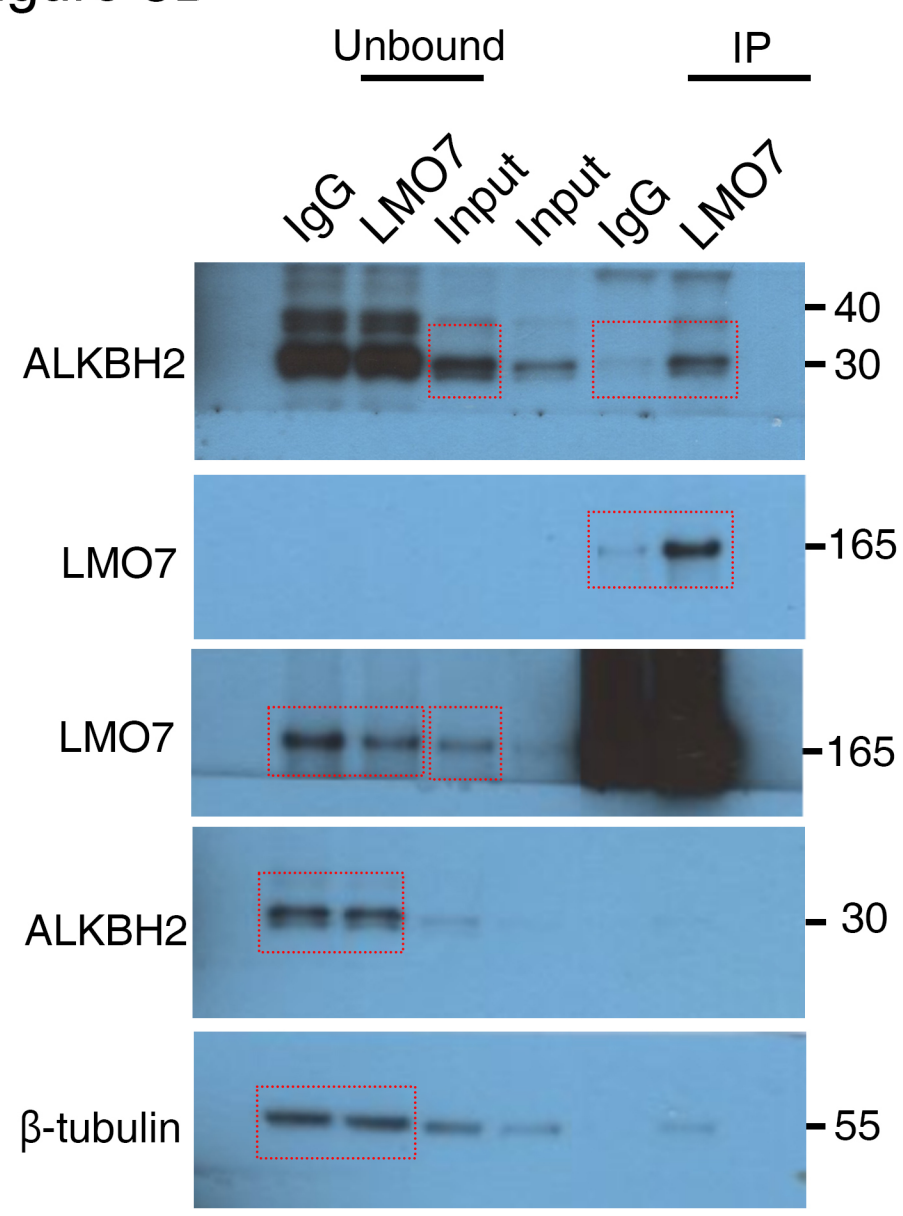

Figure 3E

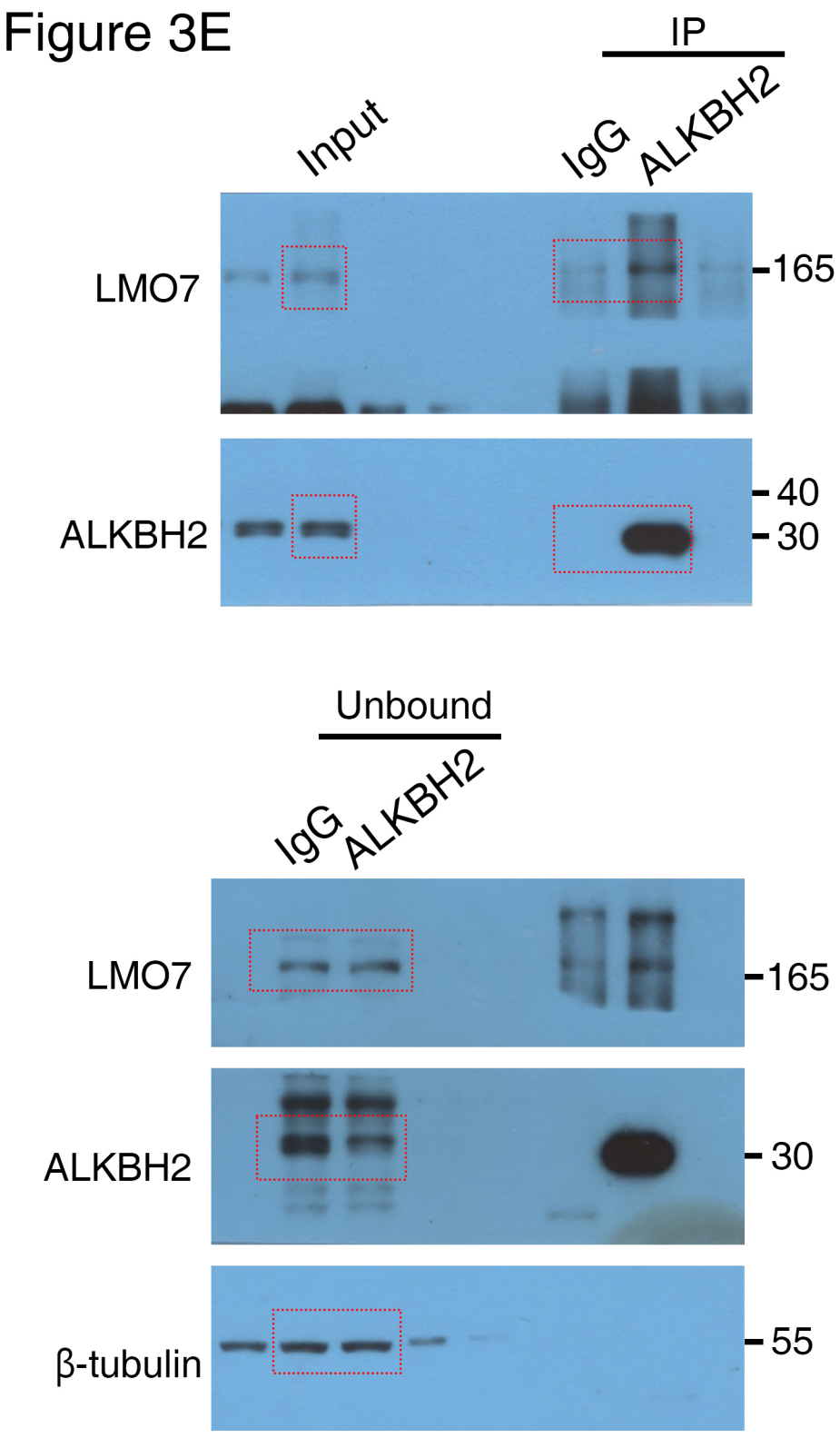

Figure 3F

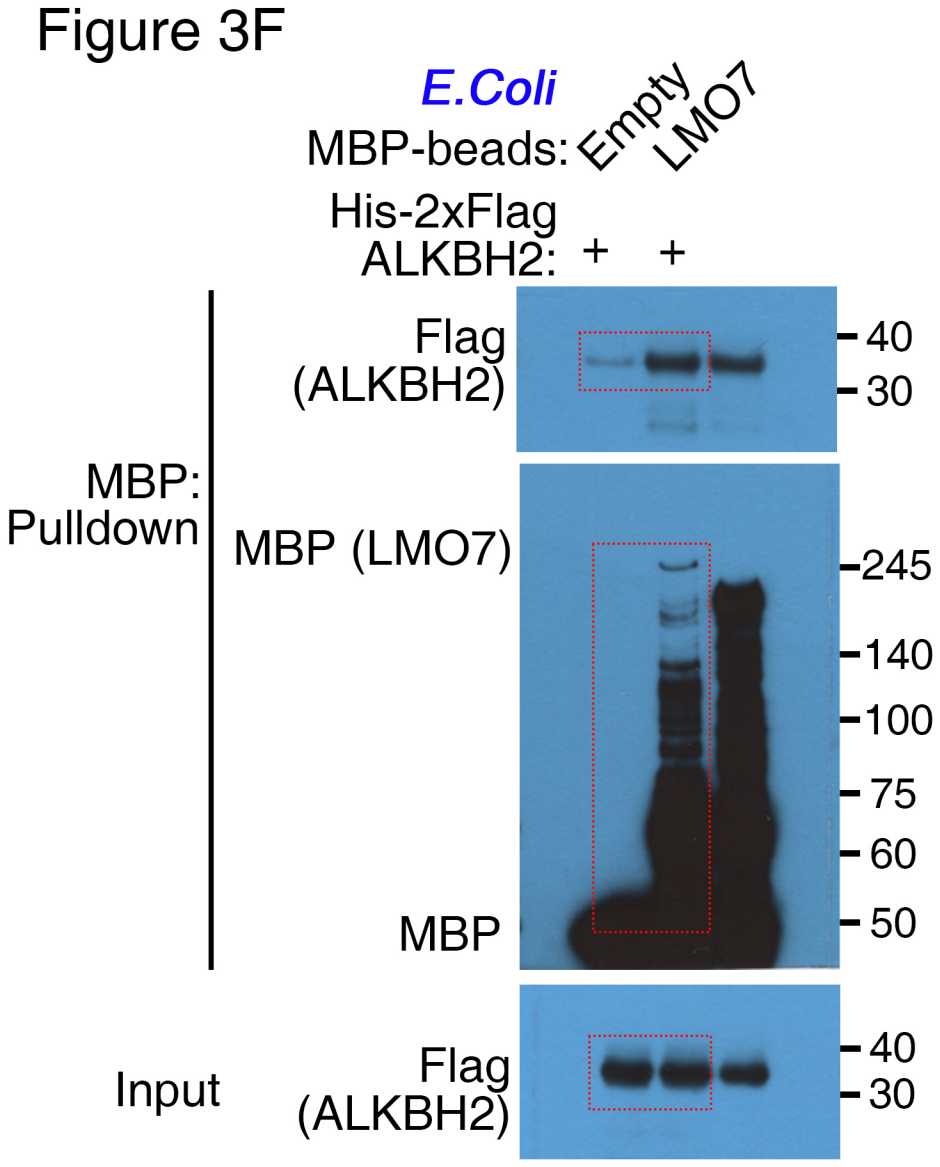

Figure 3H

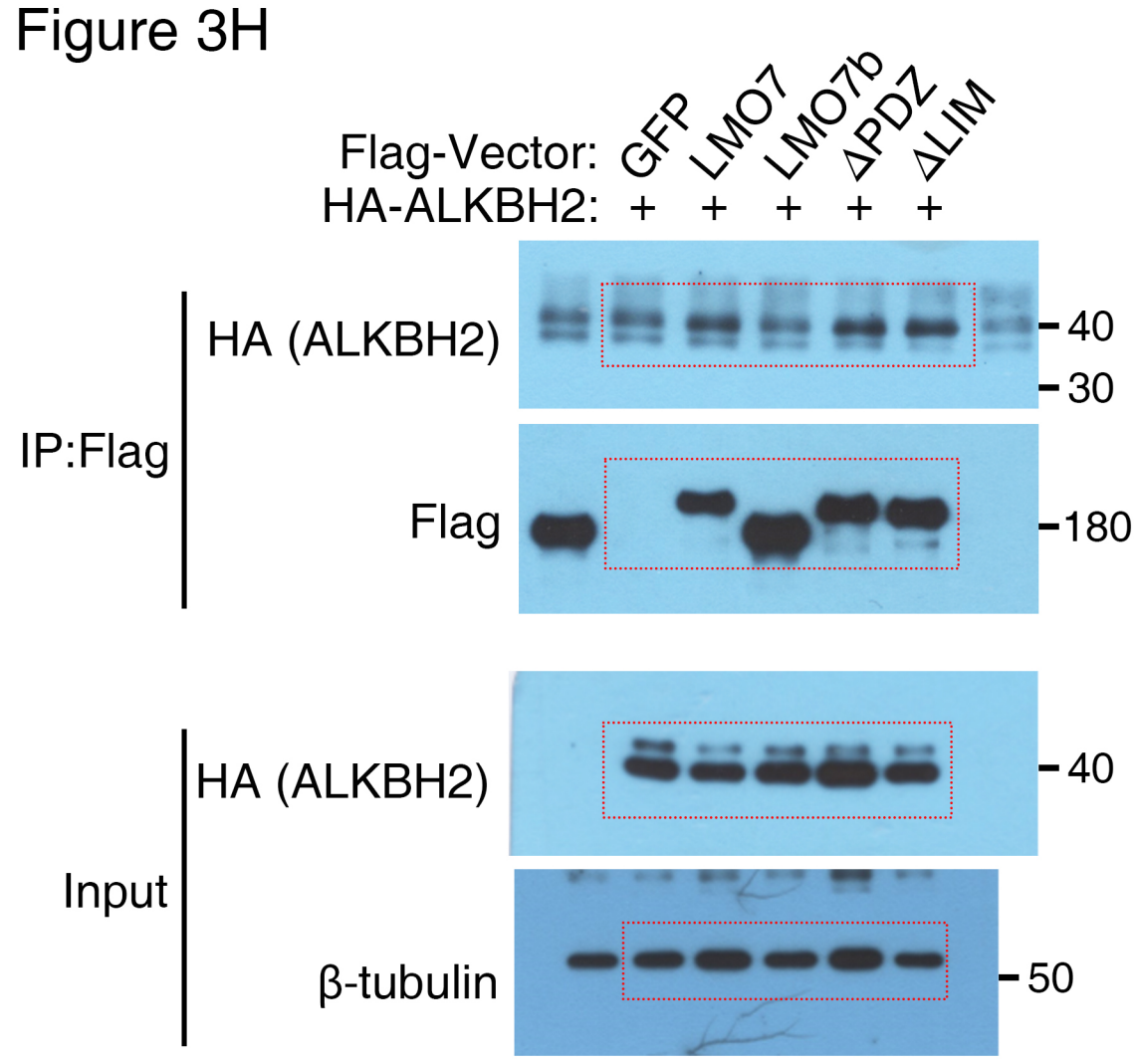

Figure 4A

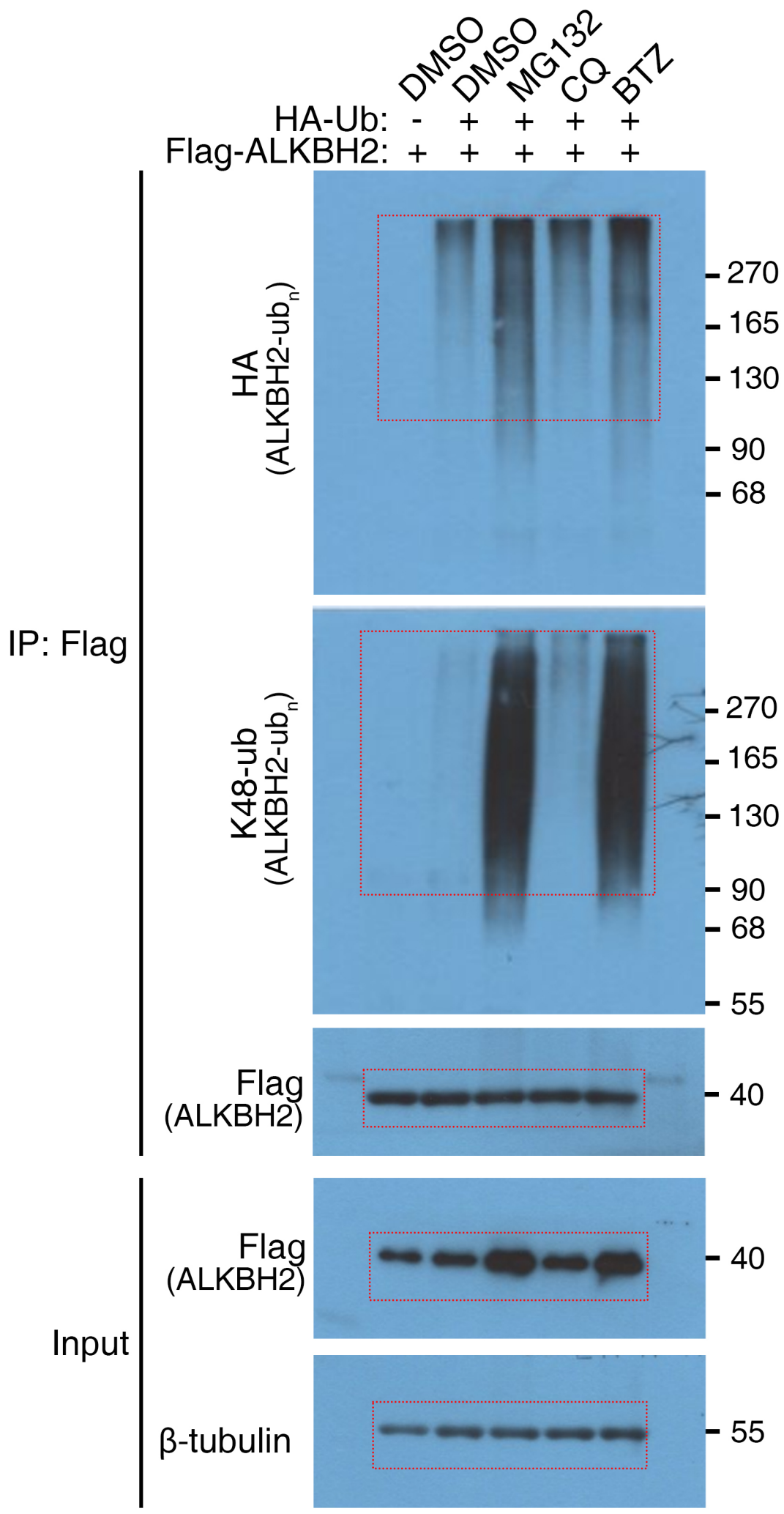

Figure 4B

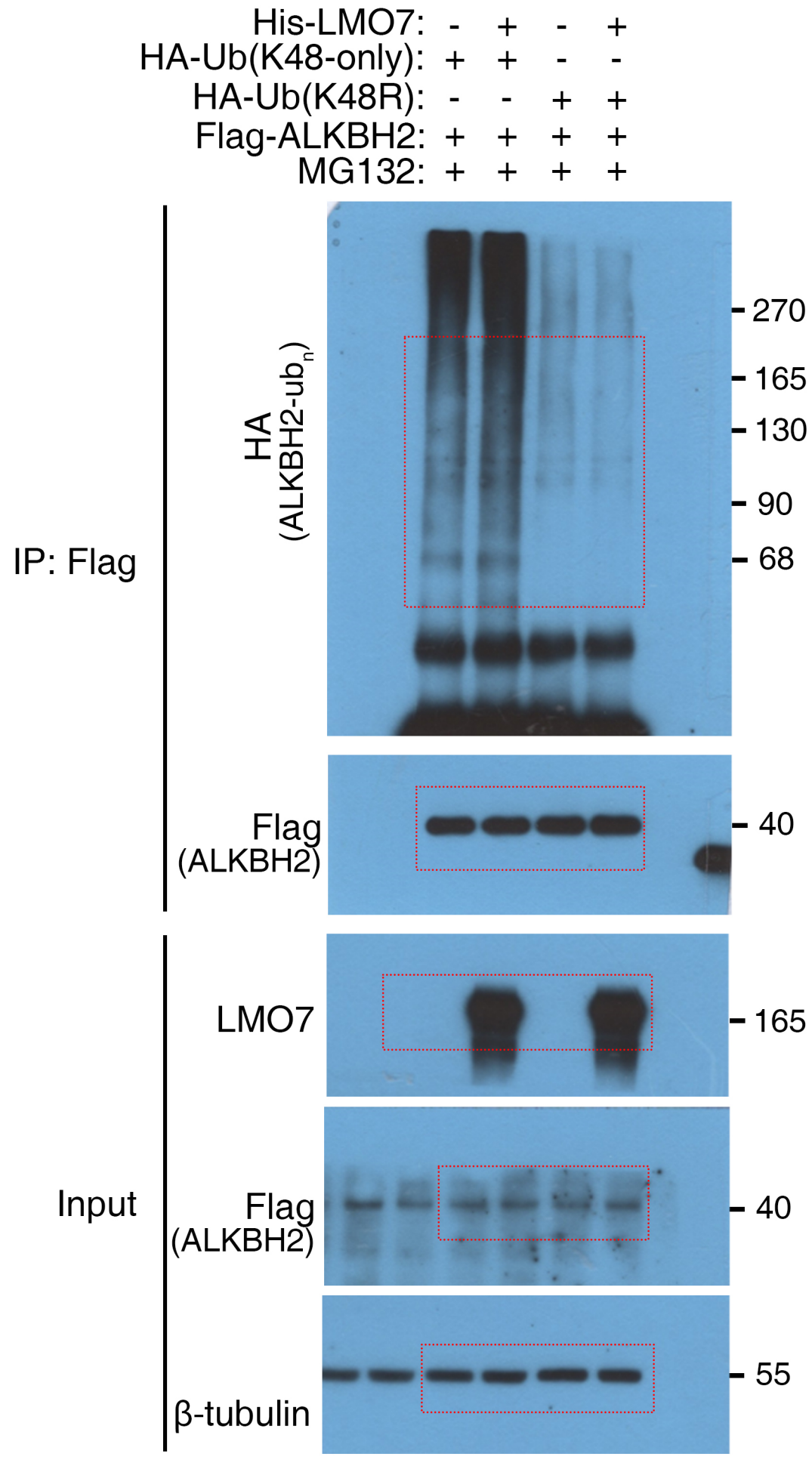

Figure 4C

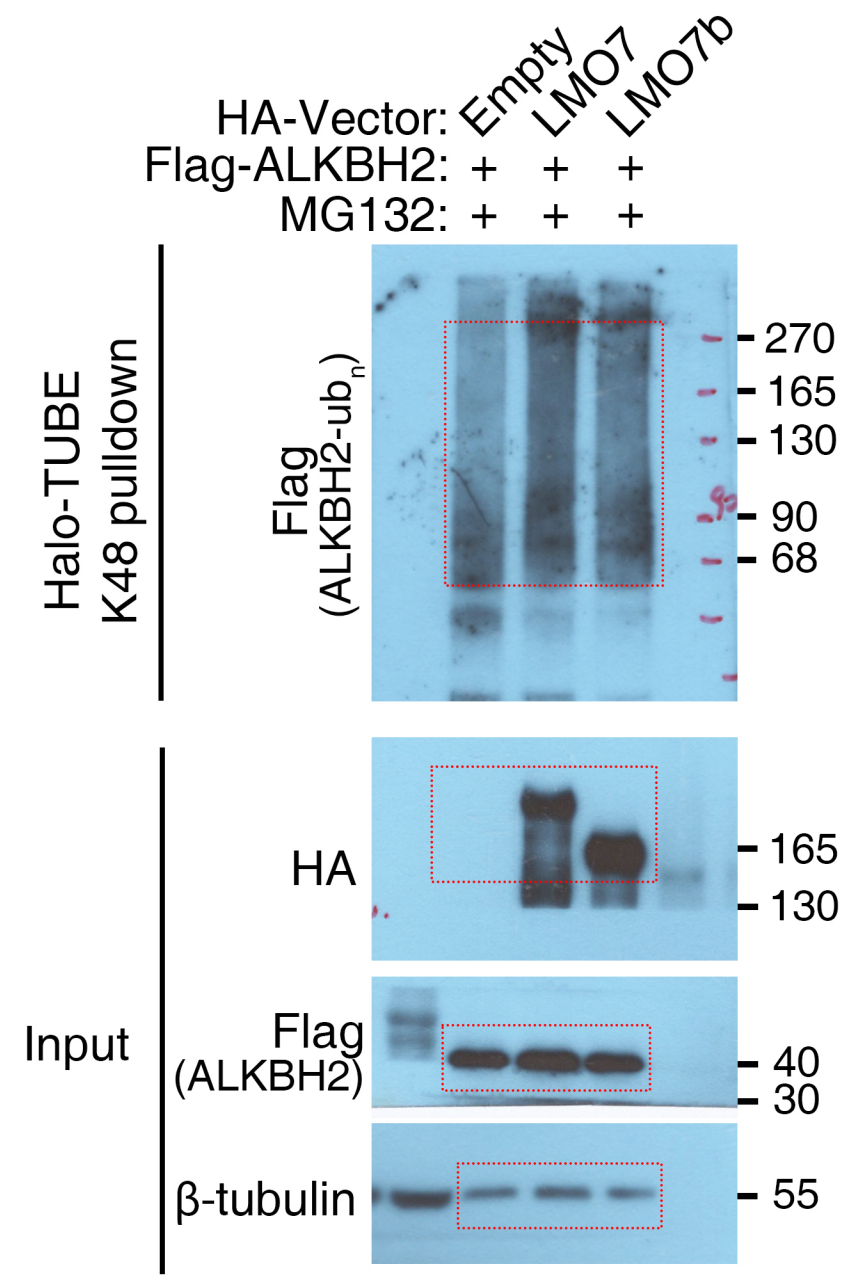

Figure 4D

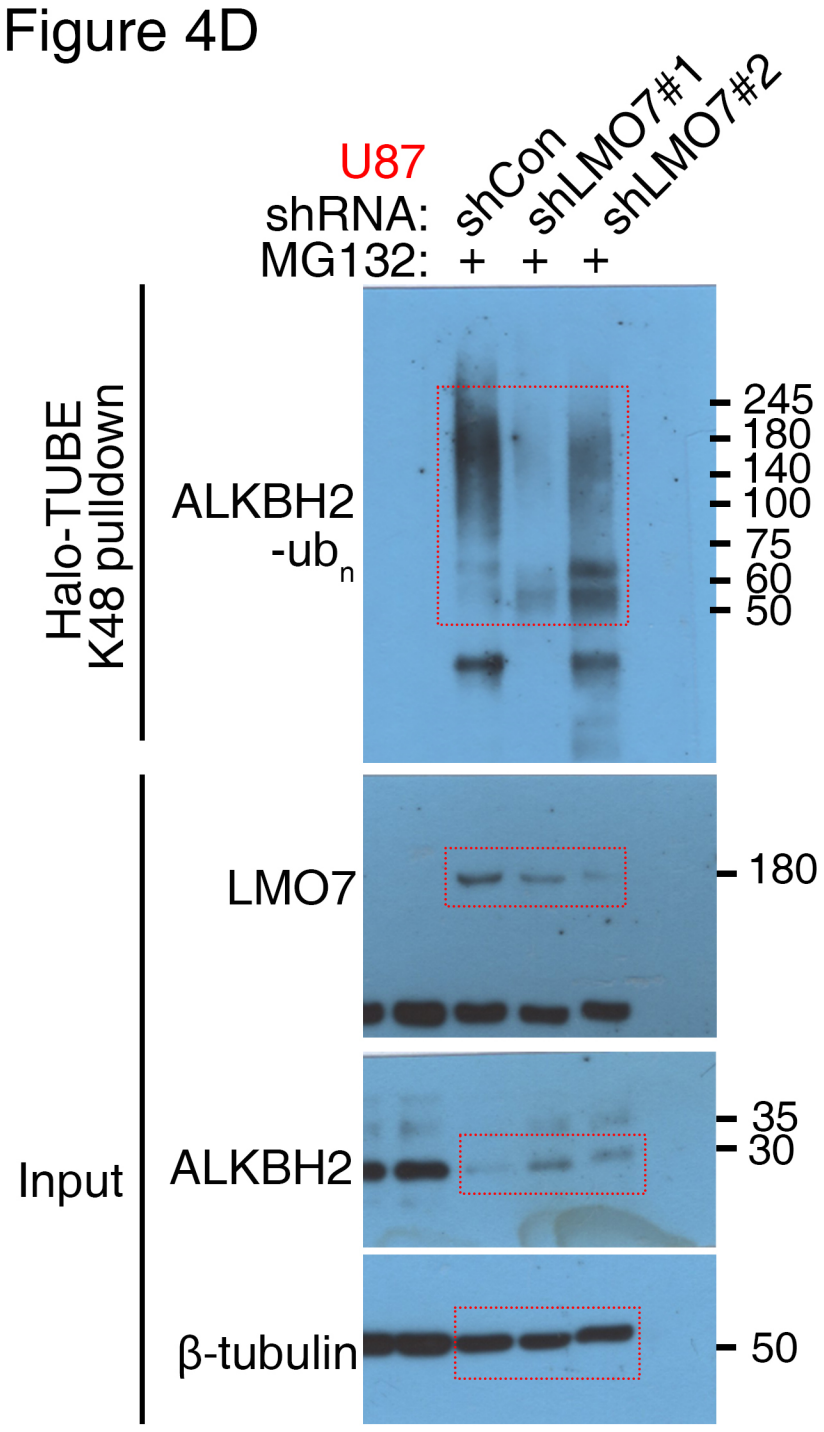

Figure 4E

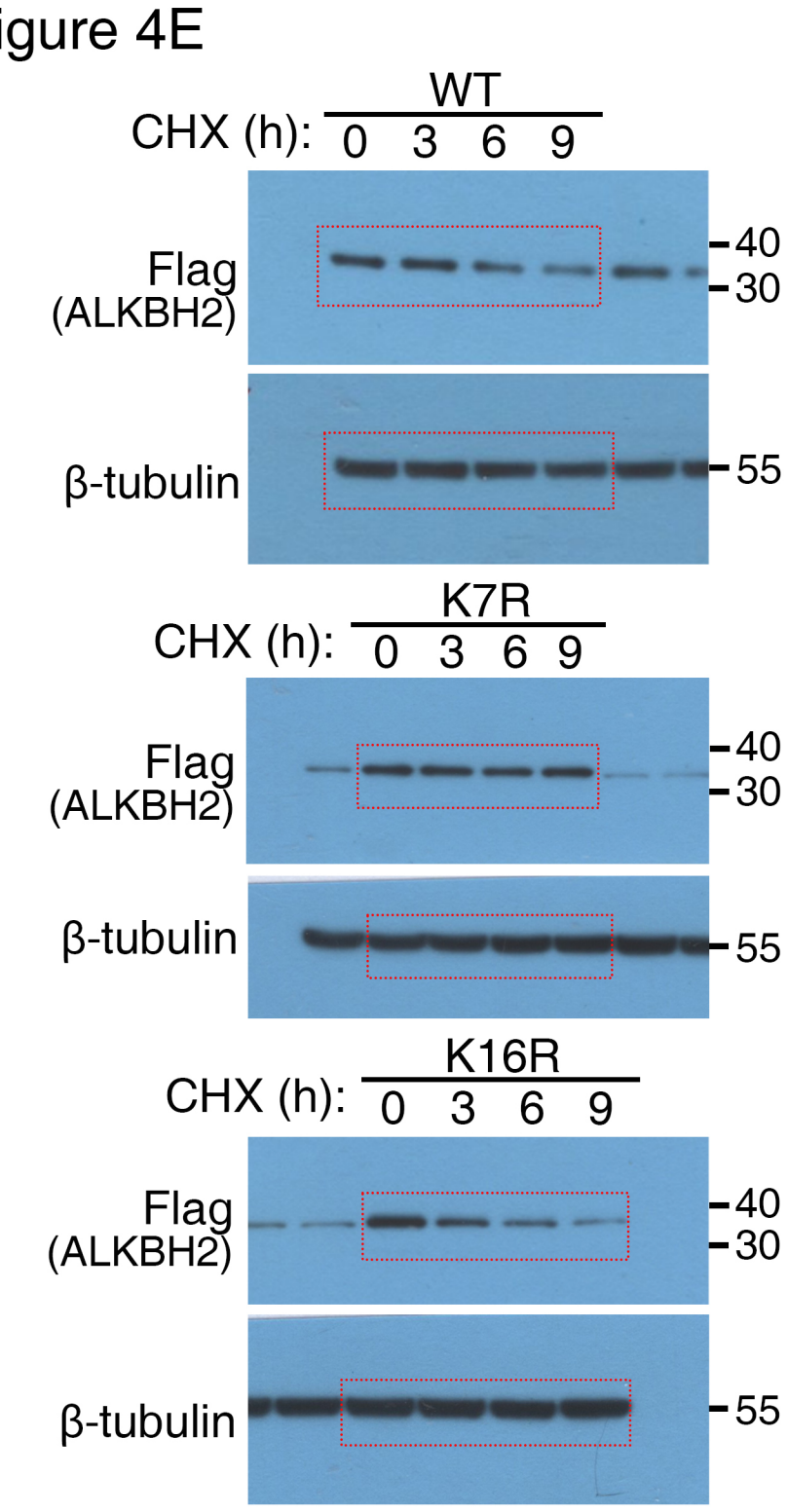

Figure 4G

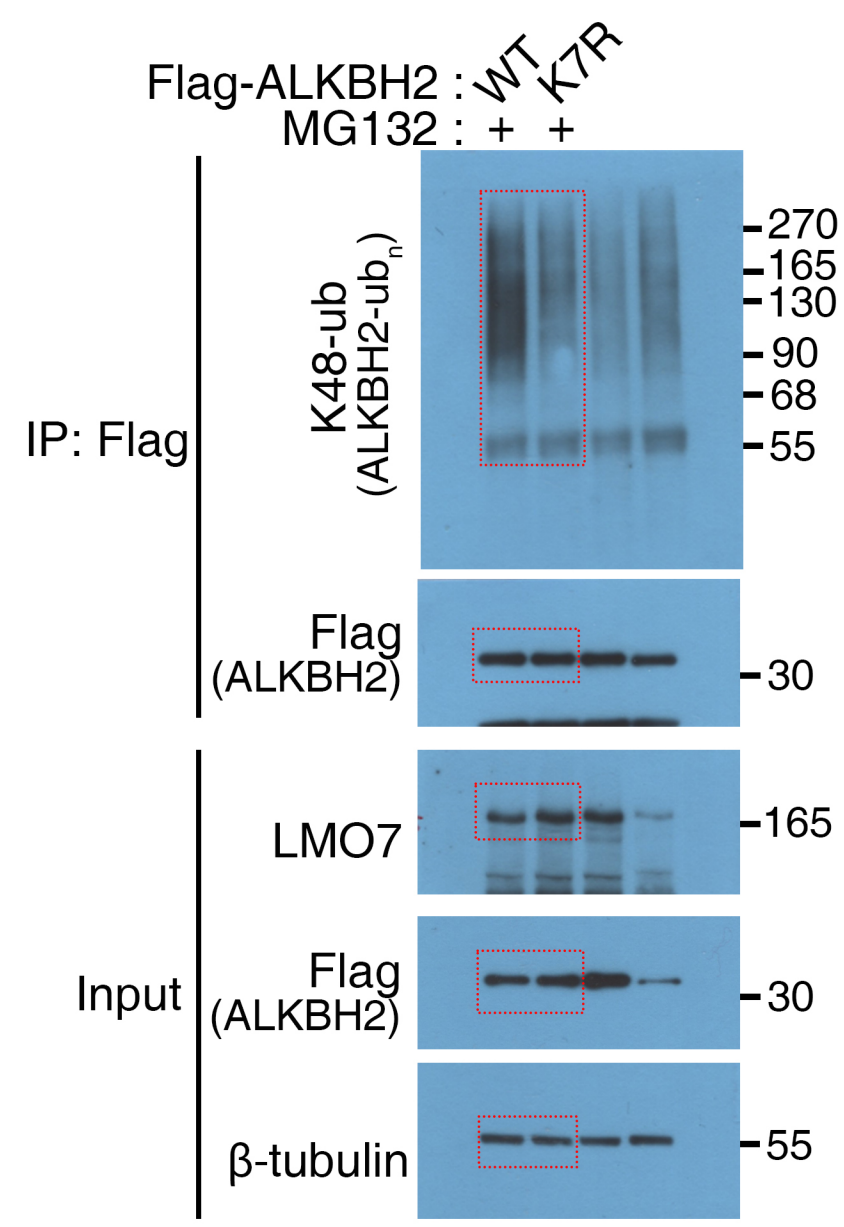

Figure 4H

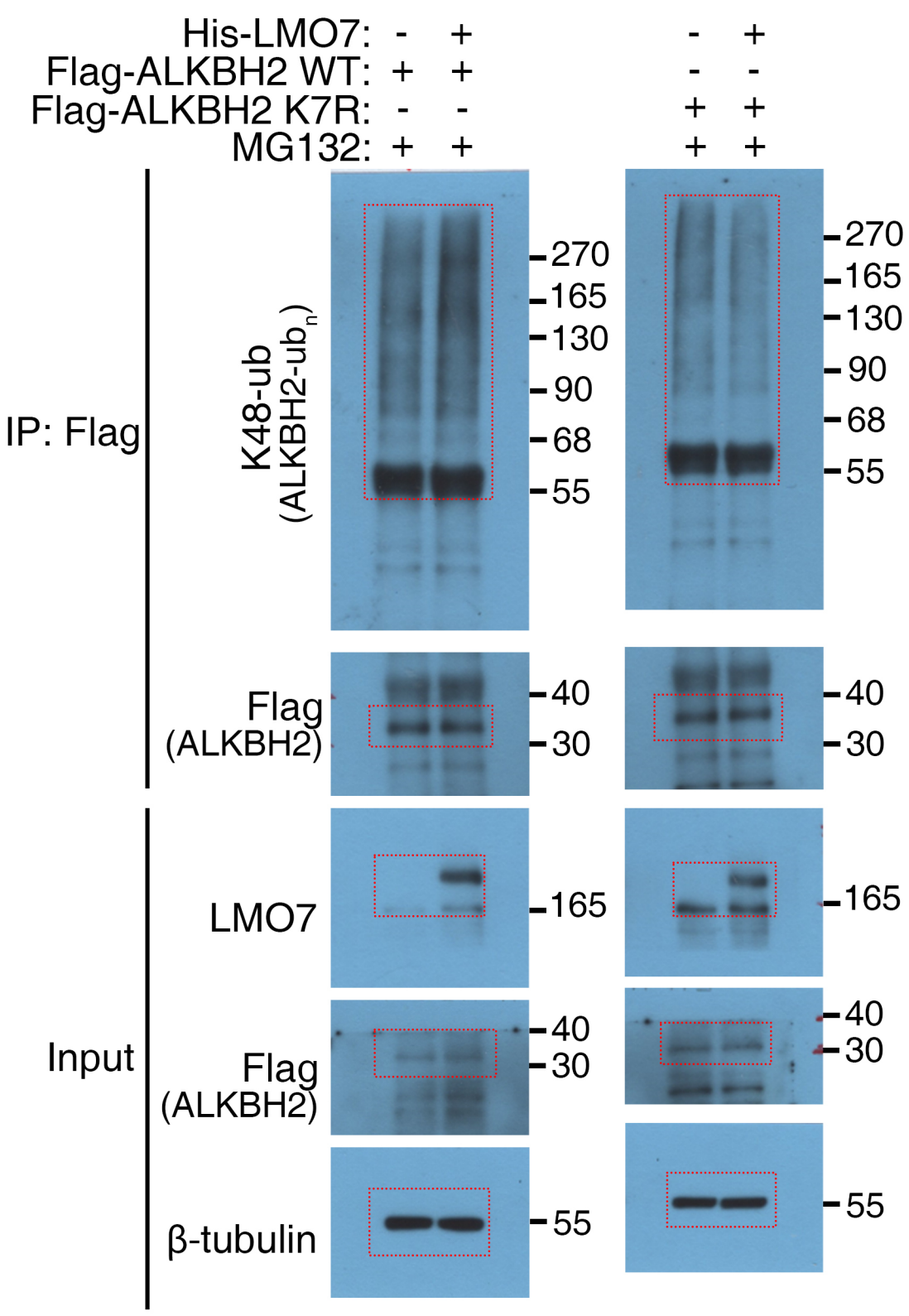

Figure 5A

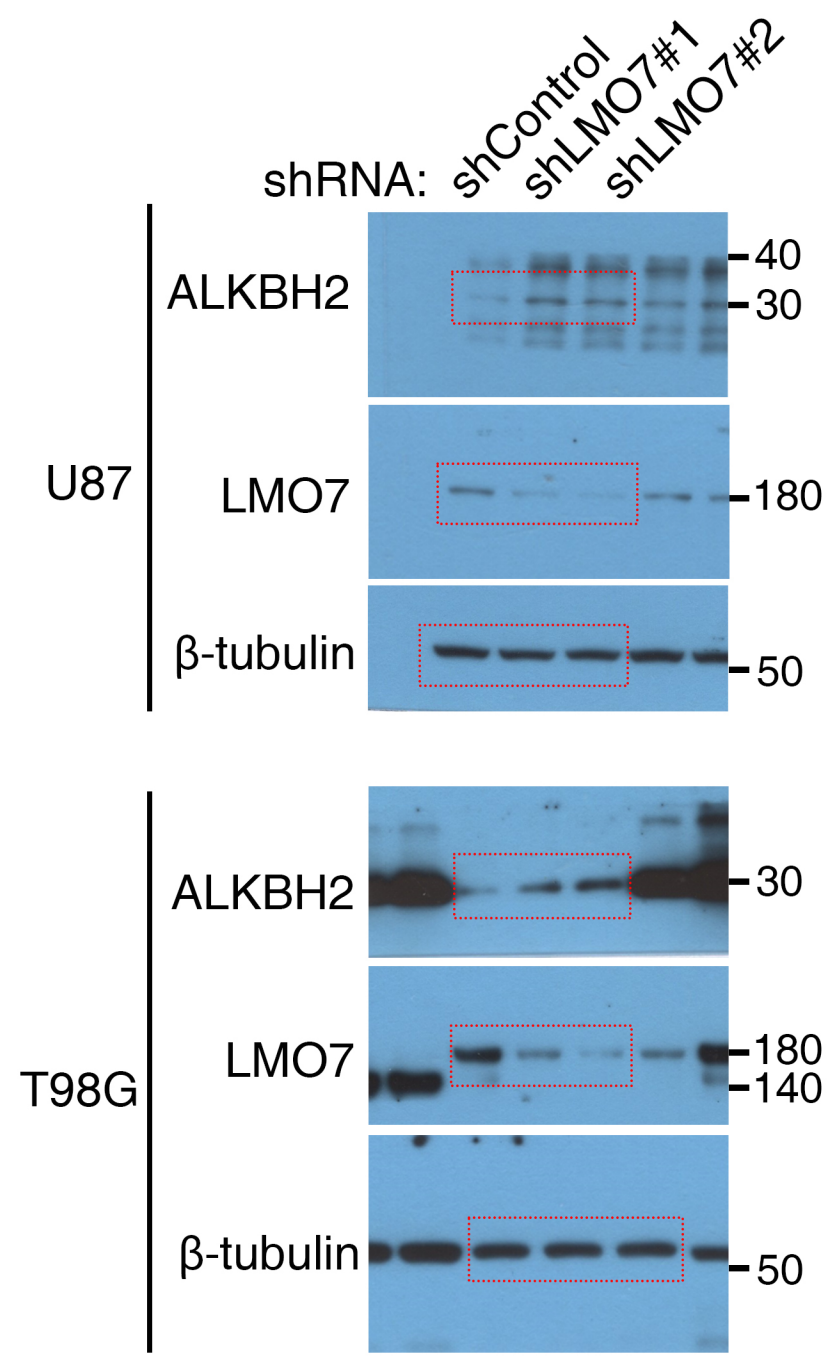

Figure 5C

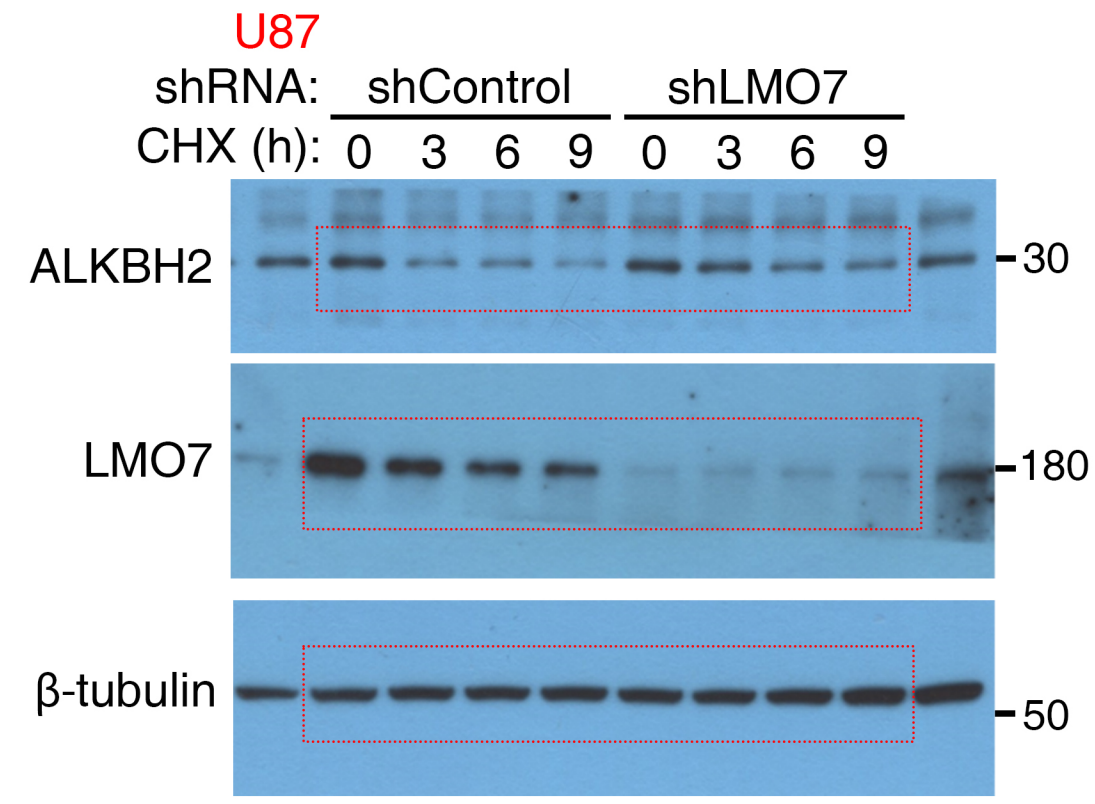

Figure 5E

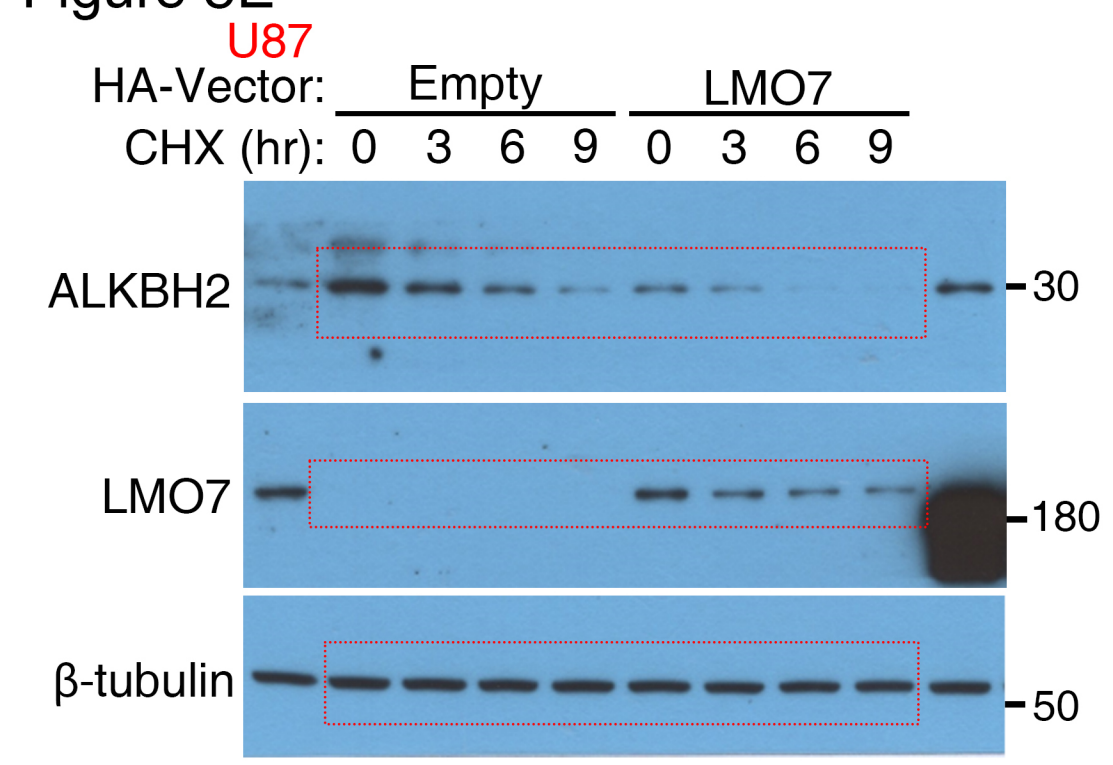

Figure 5H

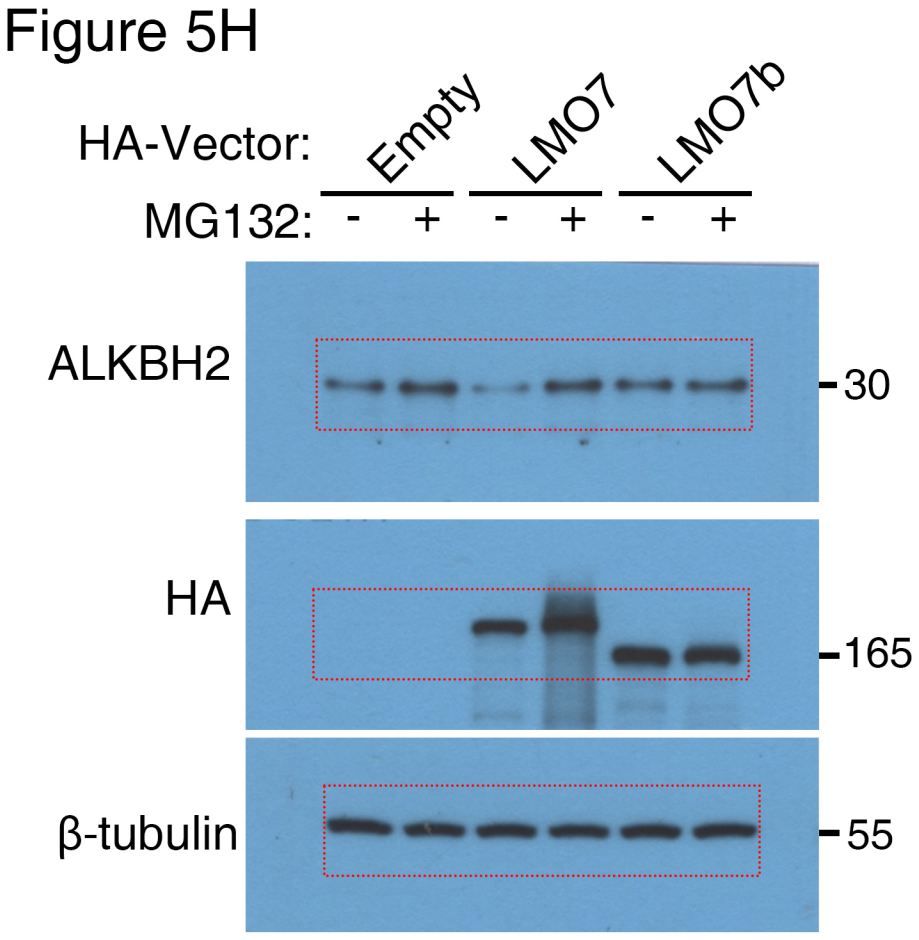

Figure 5J

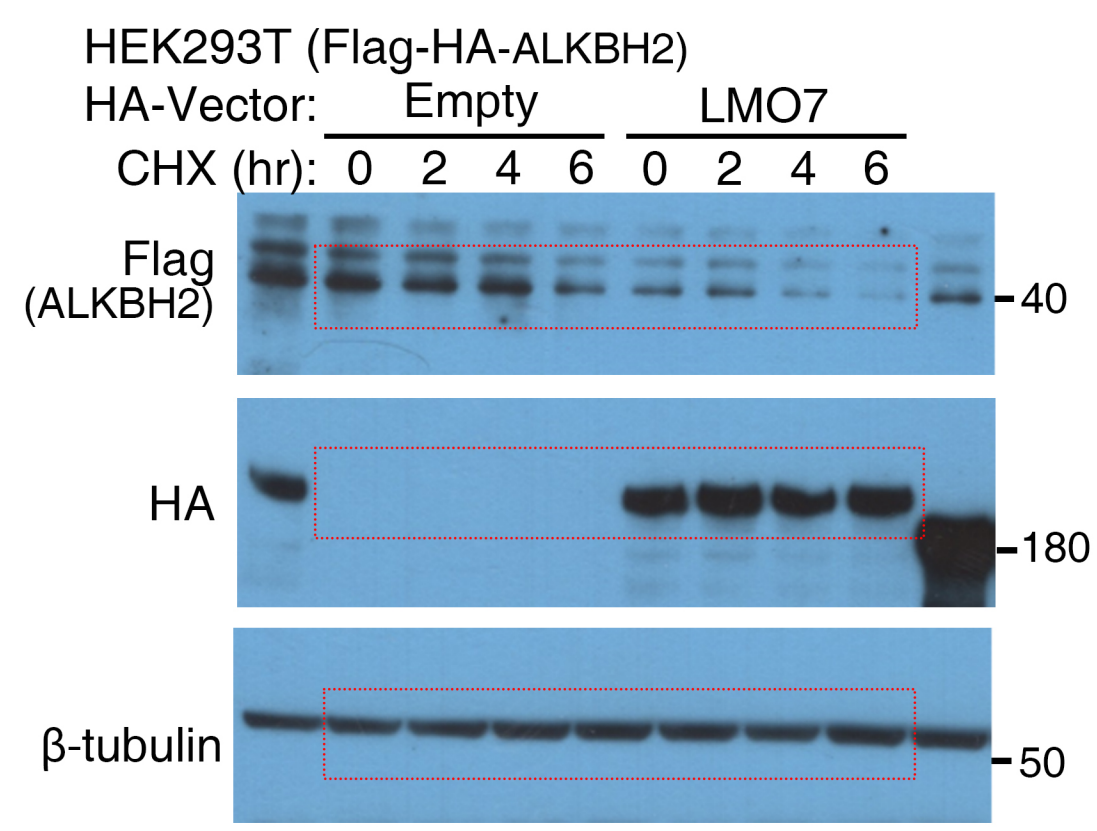

Figure 5K

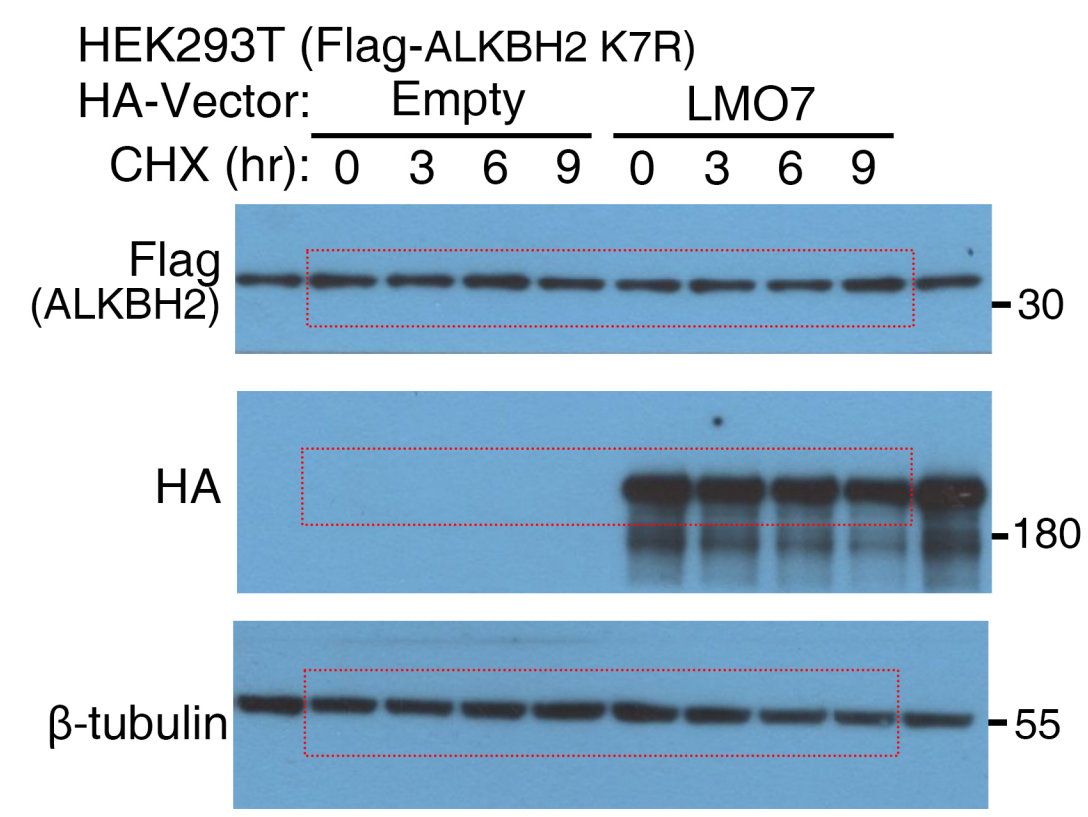

Figure 5L

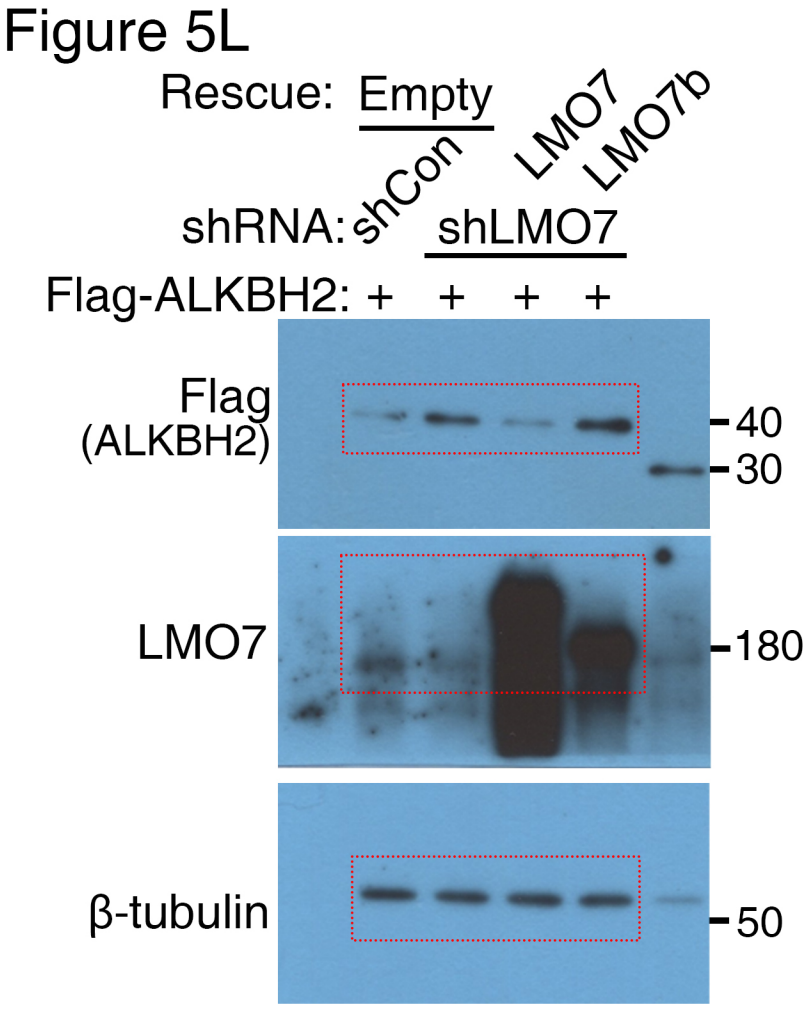

Figure 6A

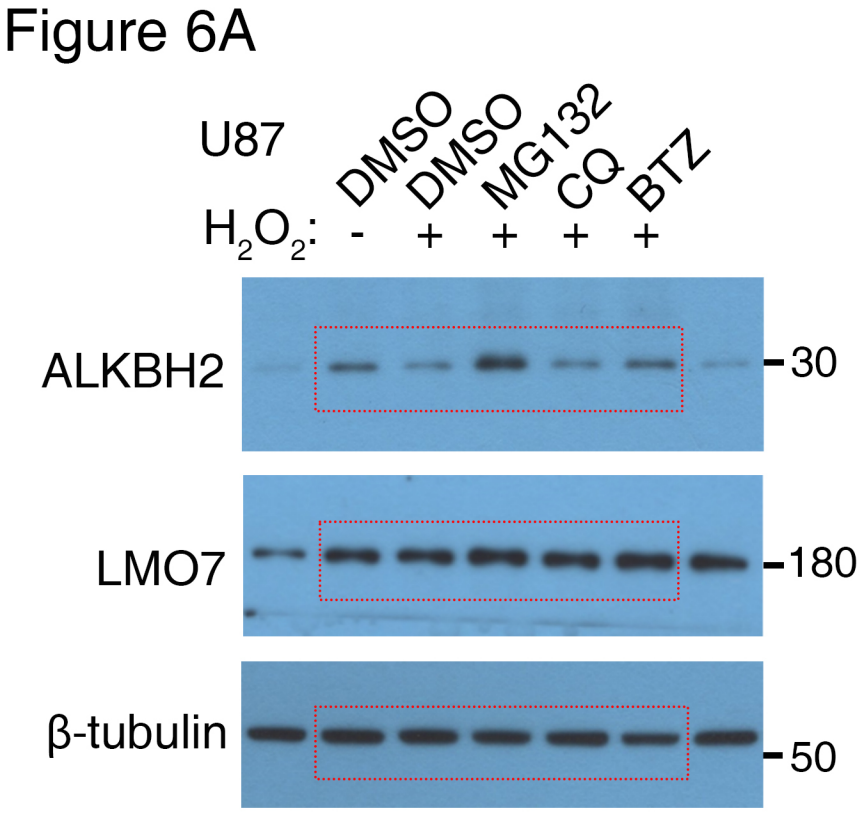

Figure 6D

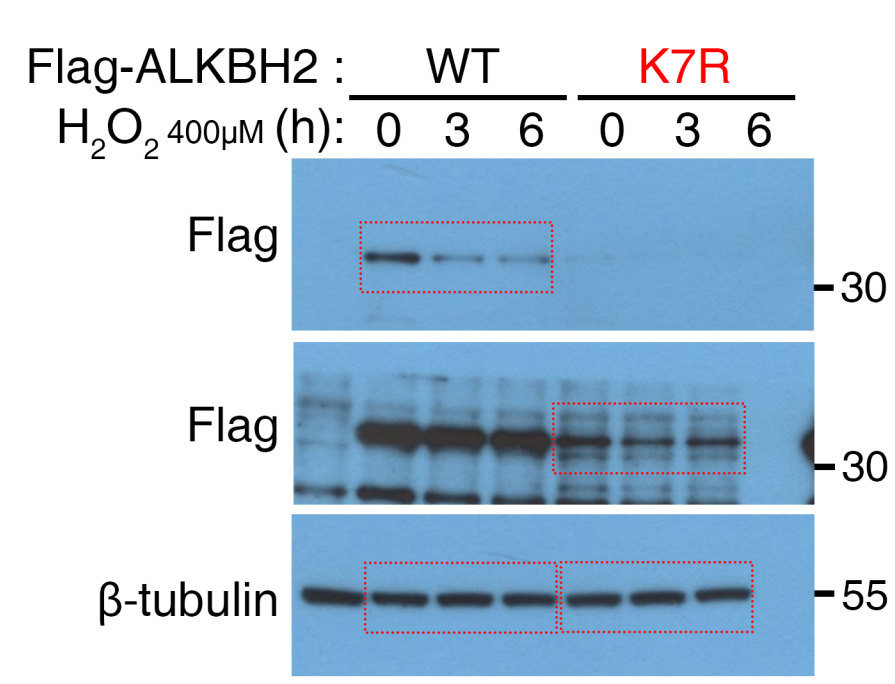

Figure 6F

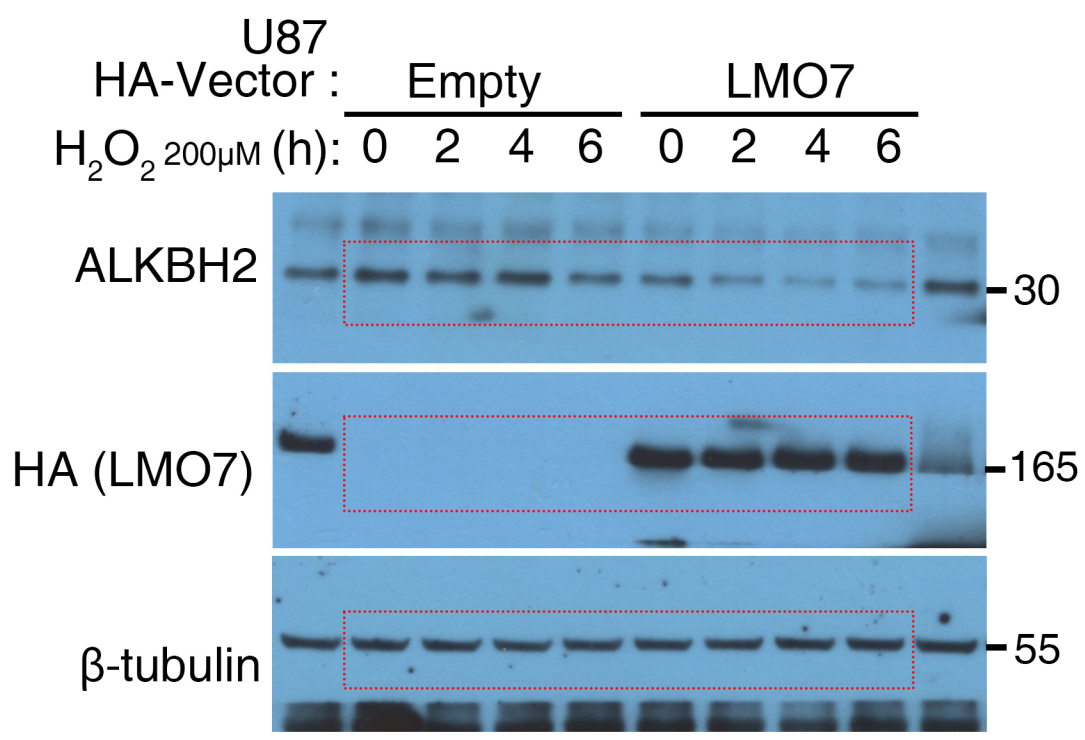

Figure 6H

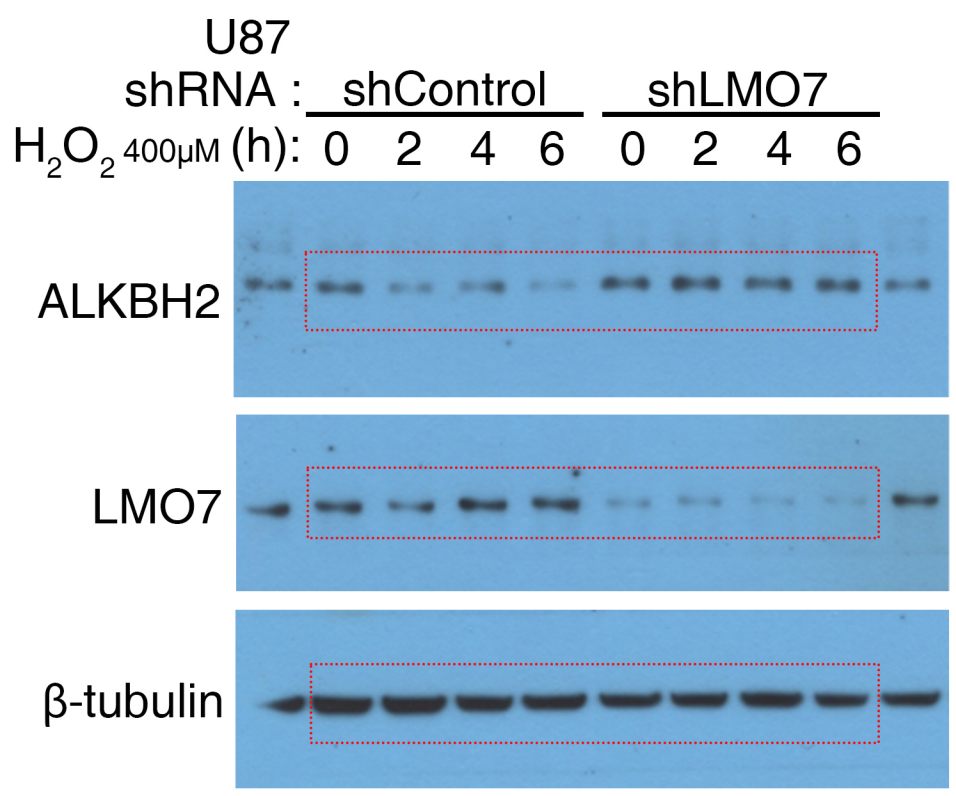

Figure 6J

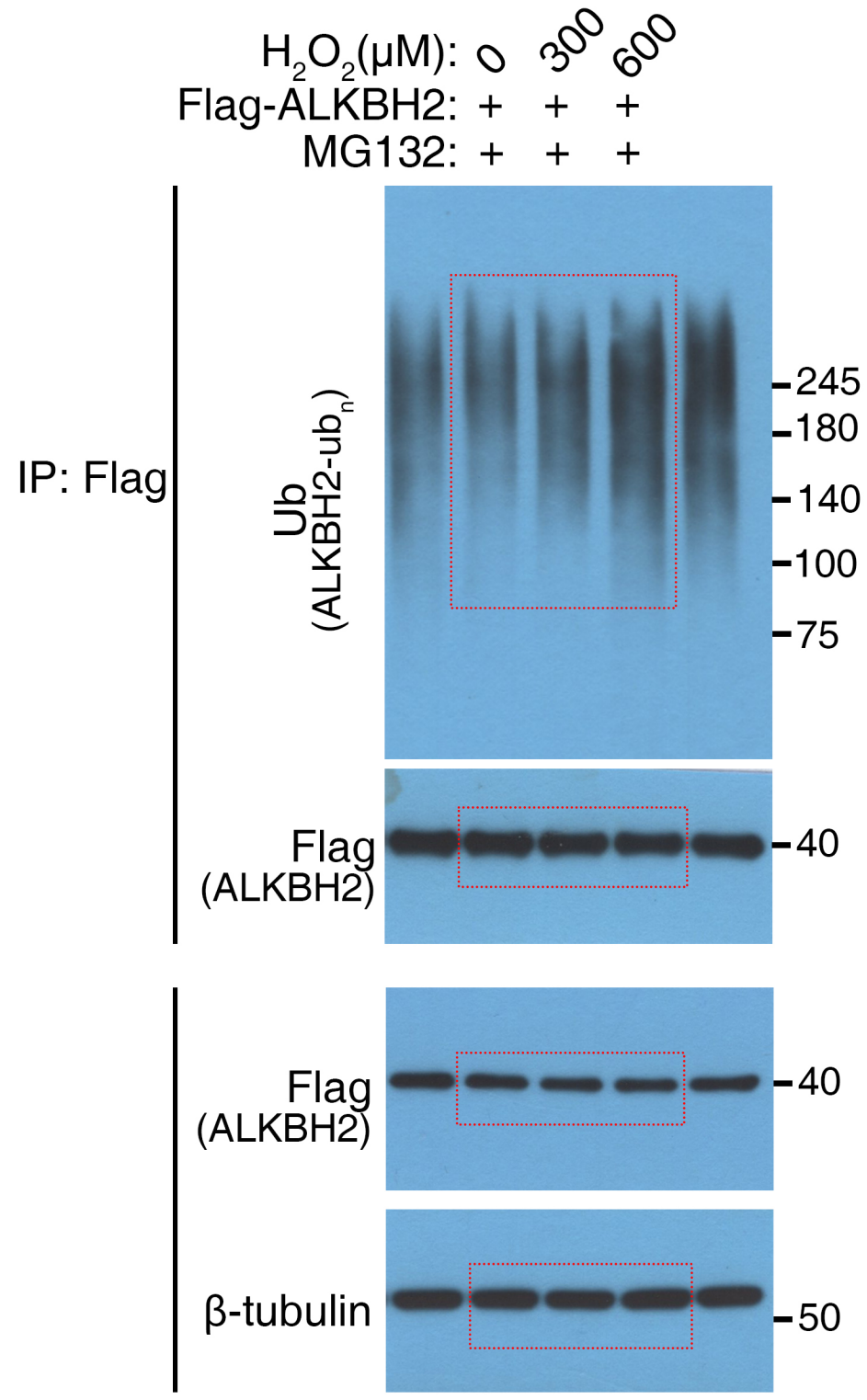

Figure 6K

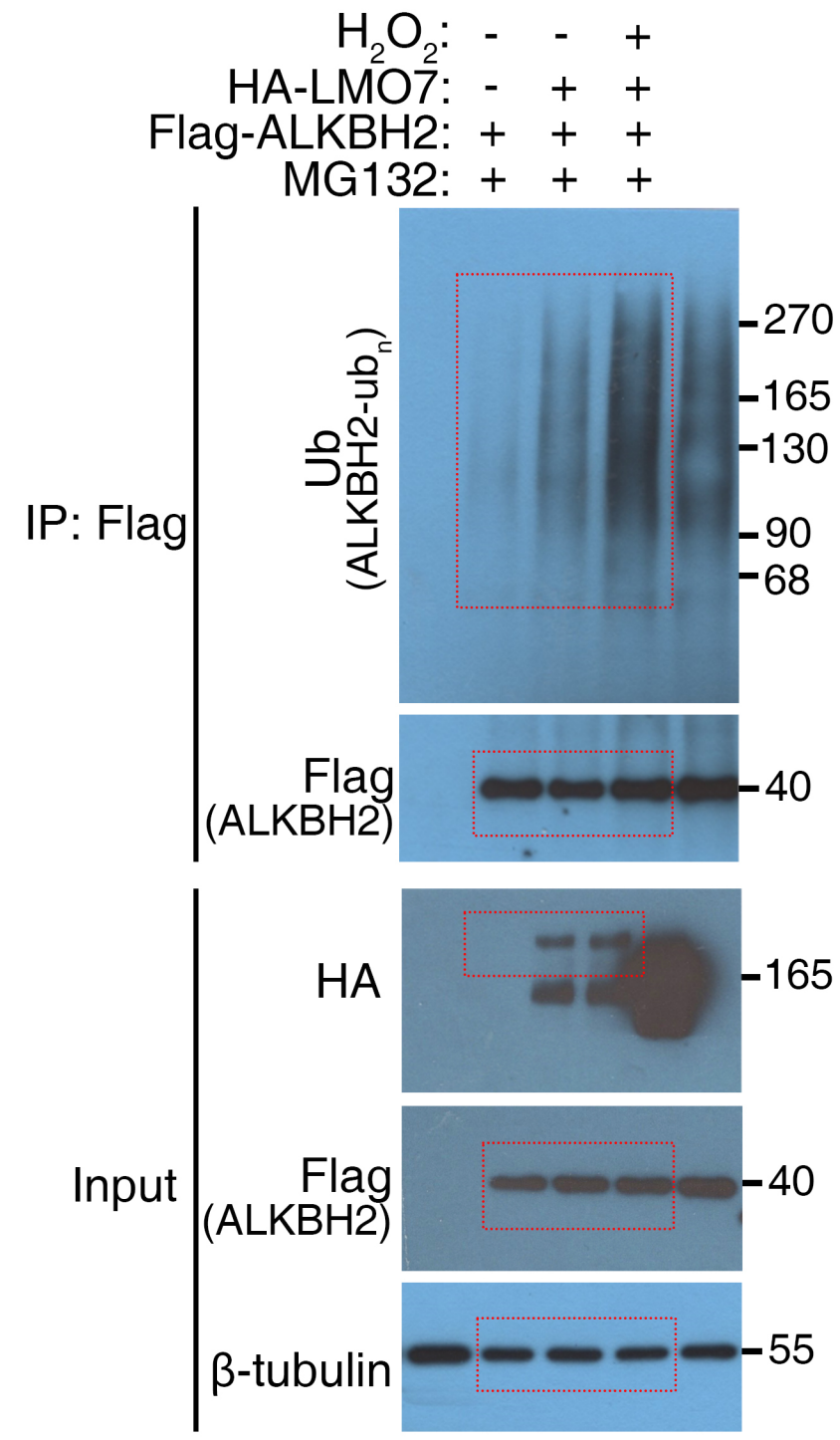

Figure 6L

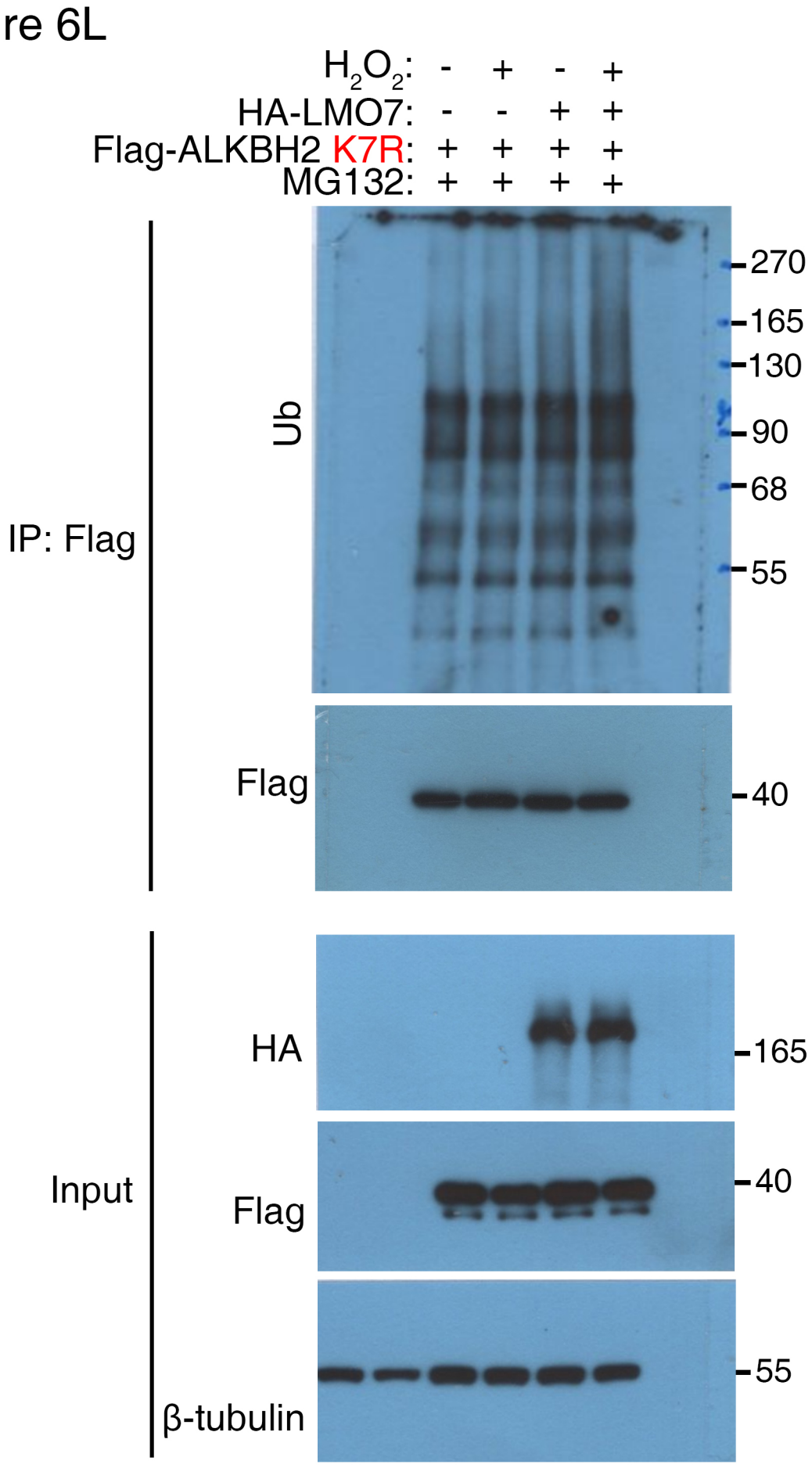

Figure 6M

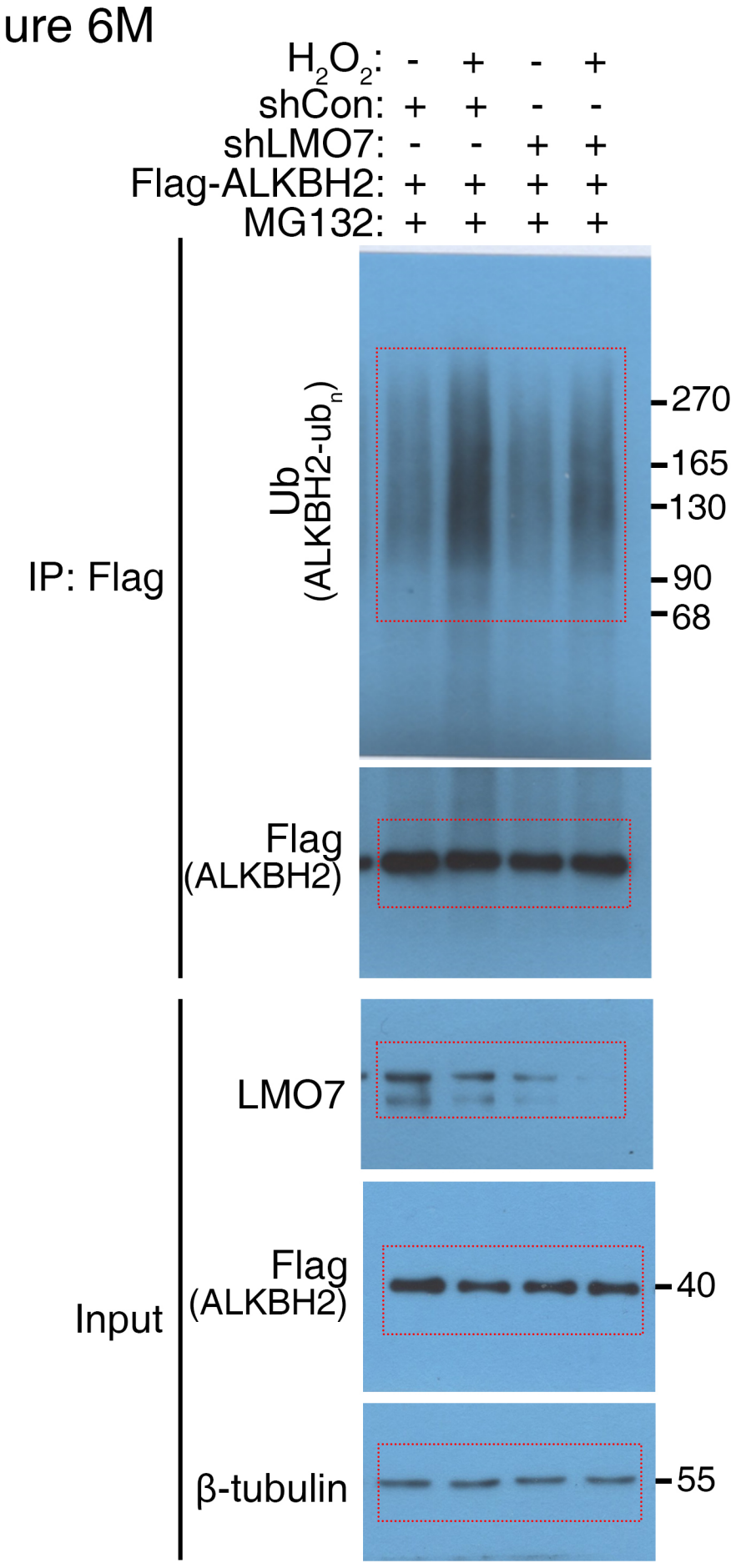

Figure 6N

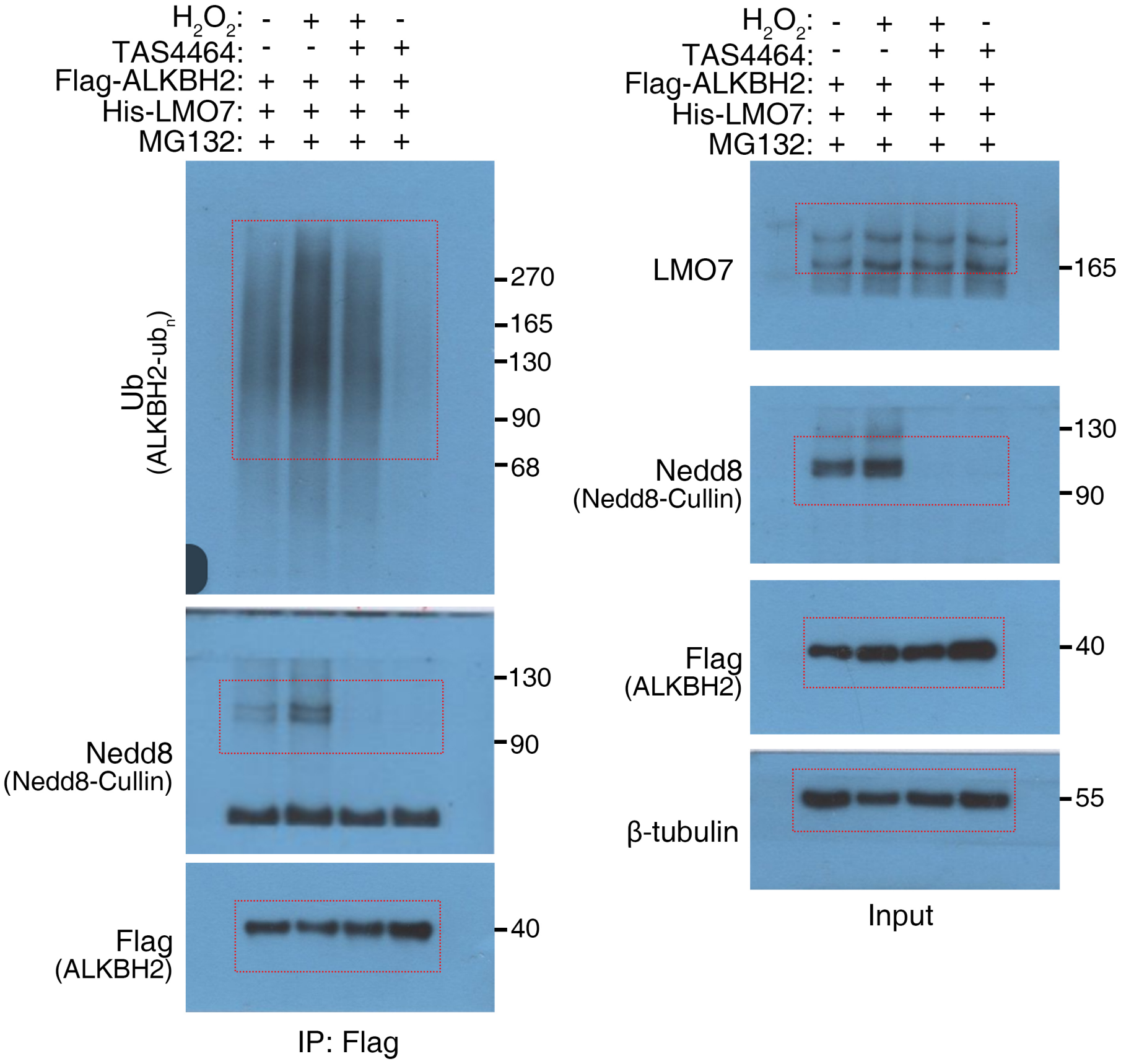

Supplementary Figure S1A

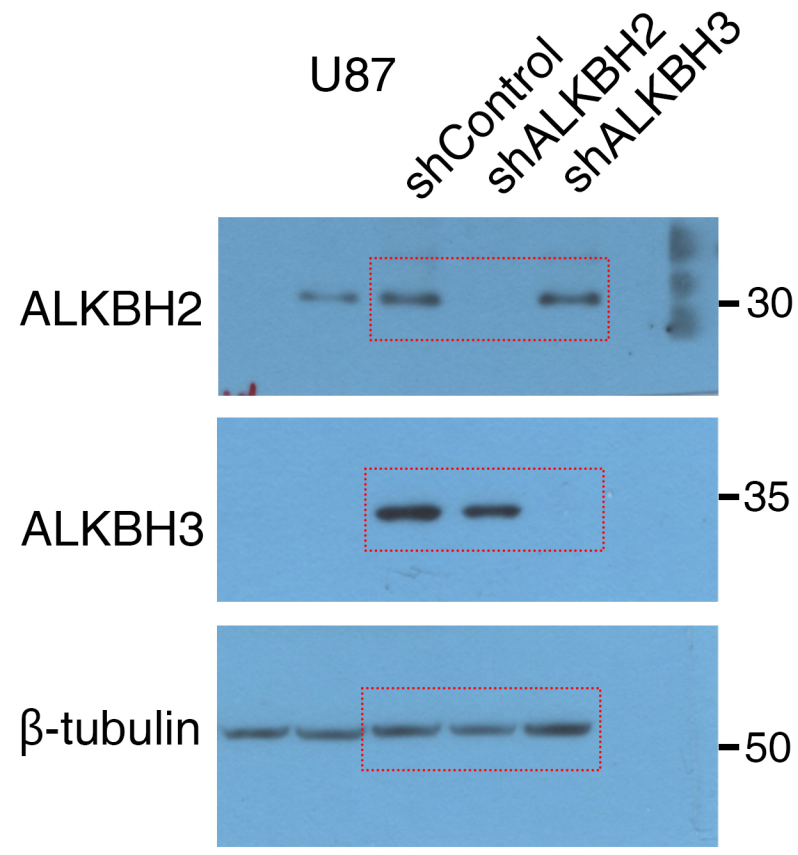

Supplementary Figure S1B

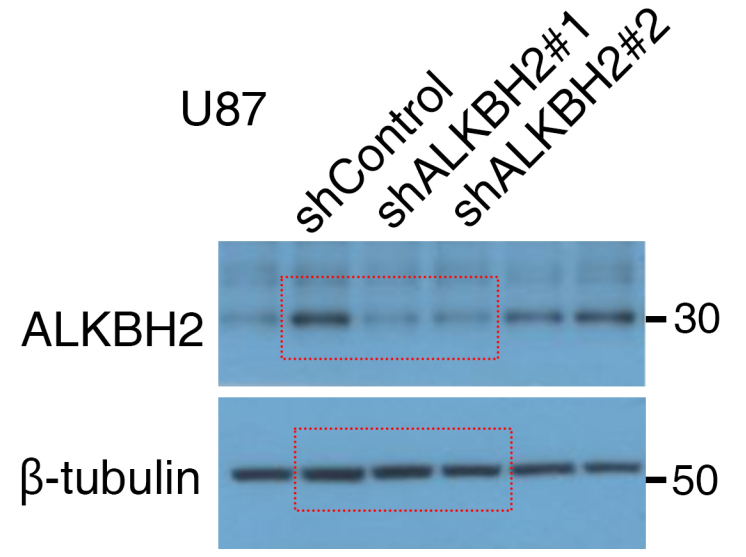

Supplementary Figure S1C

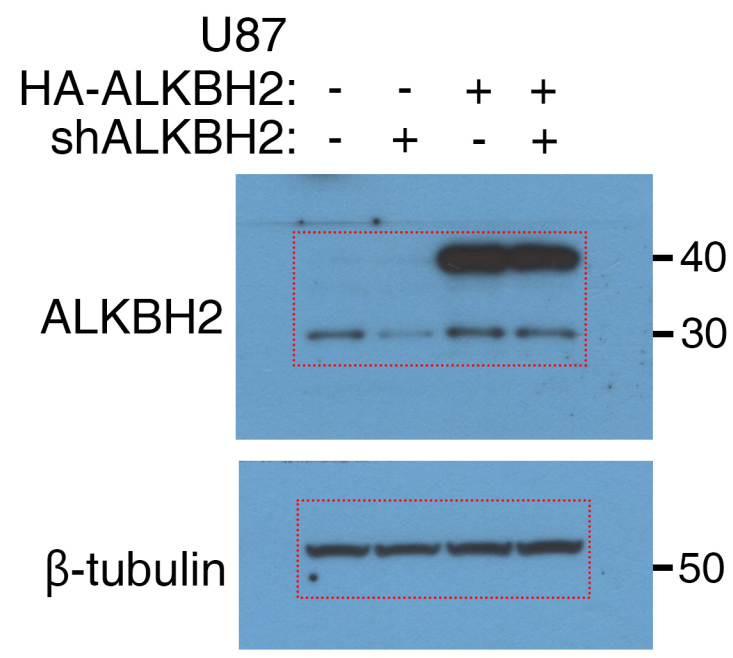

Figure 7H

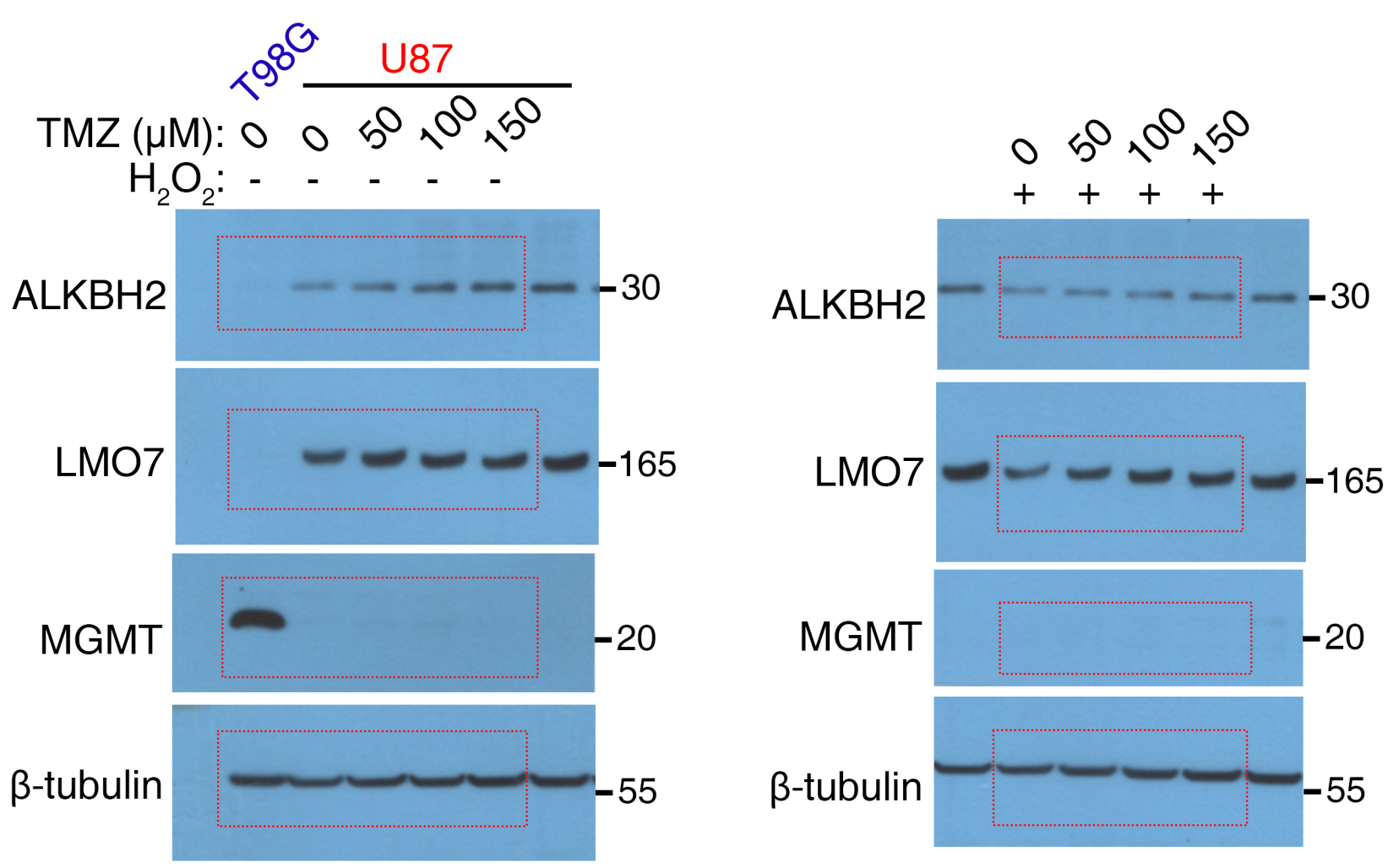

Supplementary Figure S1D

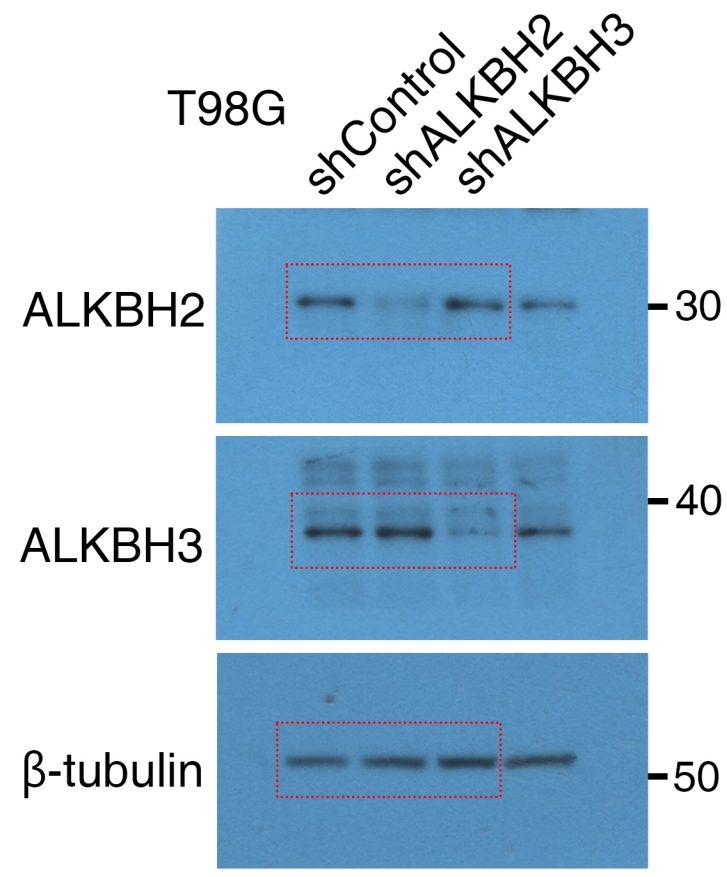

Supplementary Figure S1E

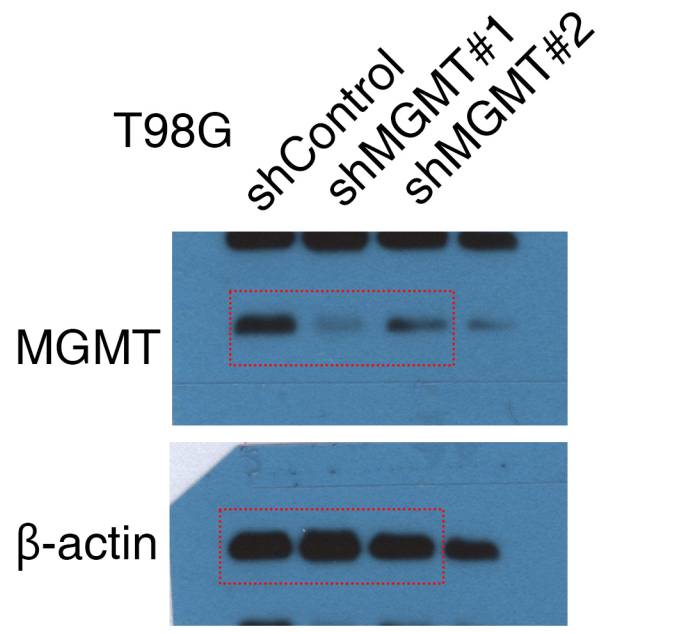

Supplementary Figure S3B

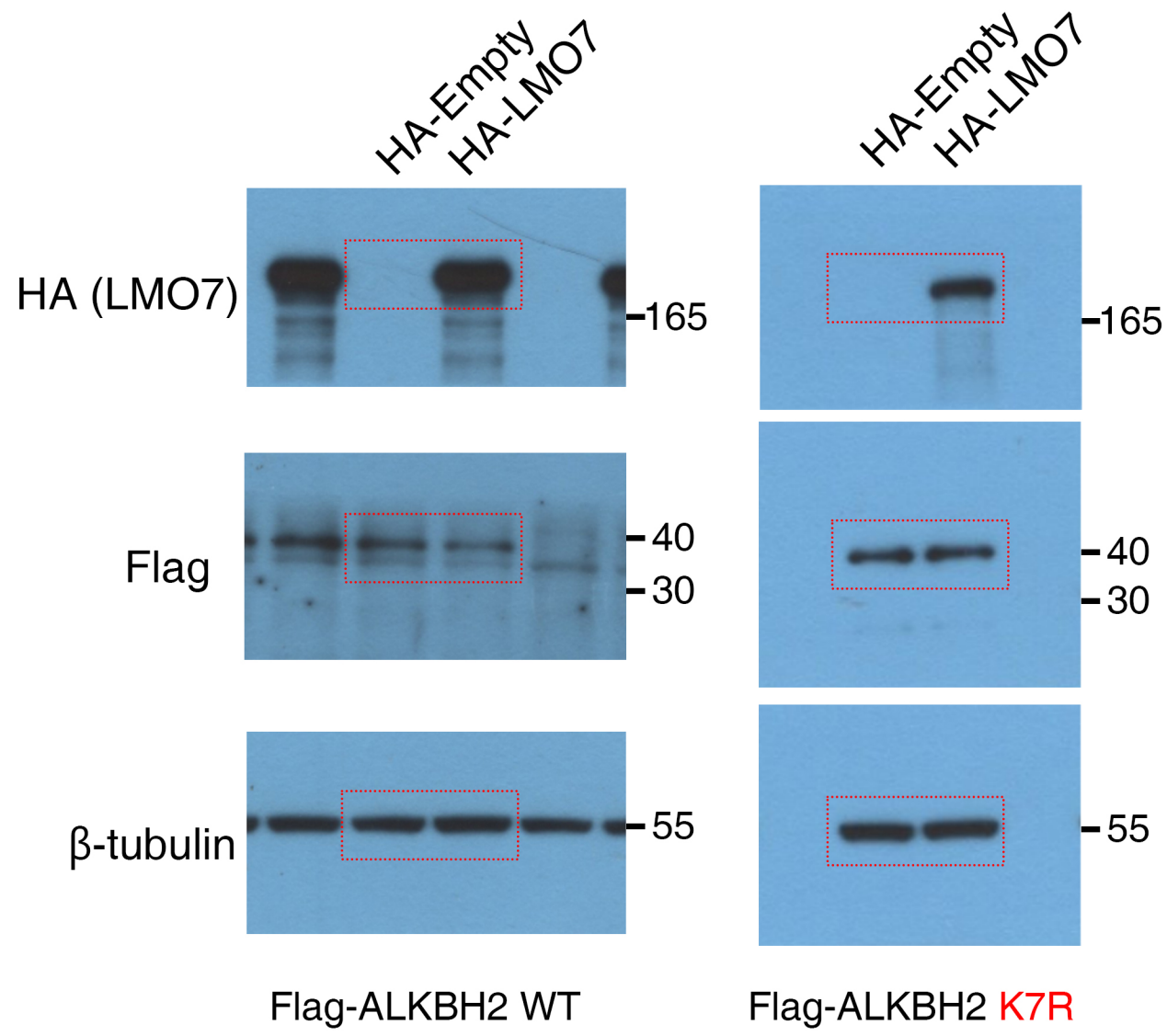

Supplementary Figure S3D

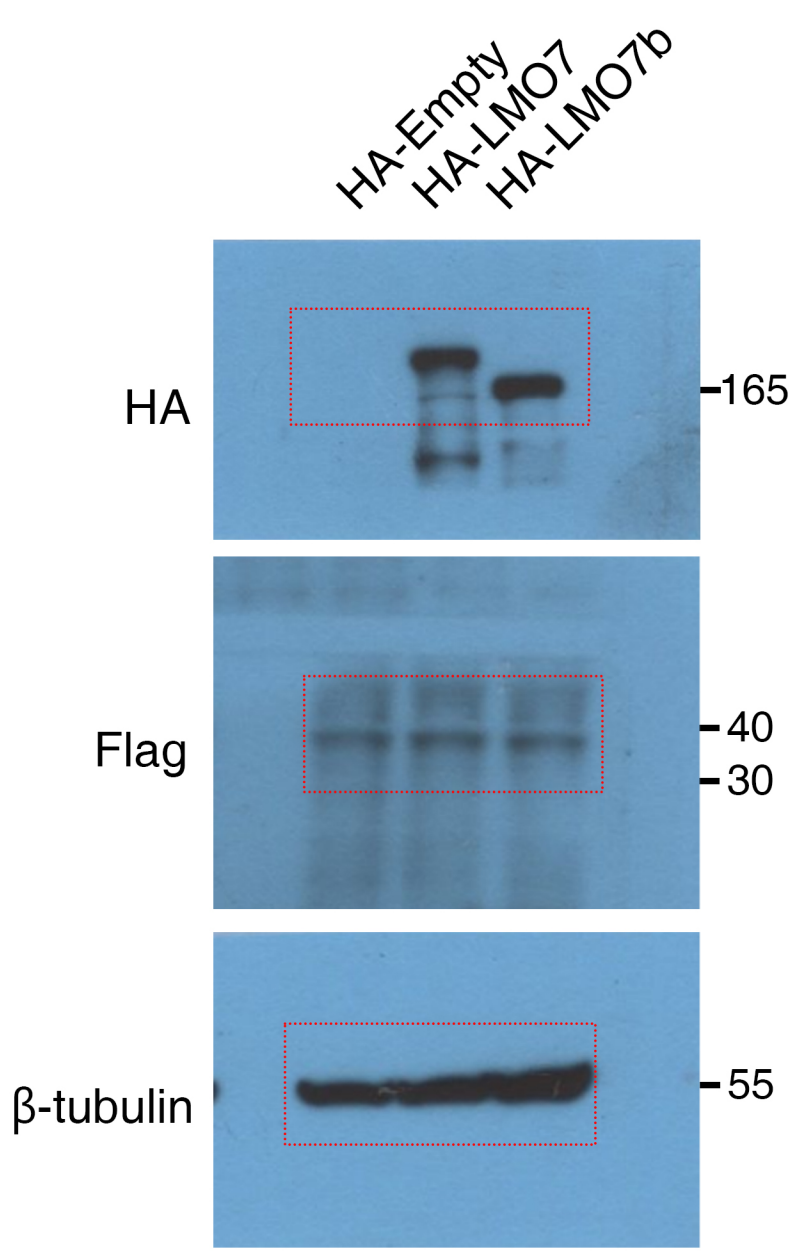

Supplementary Figure S3F

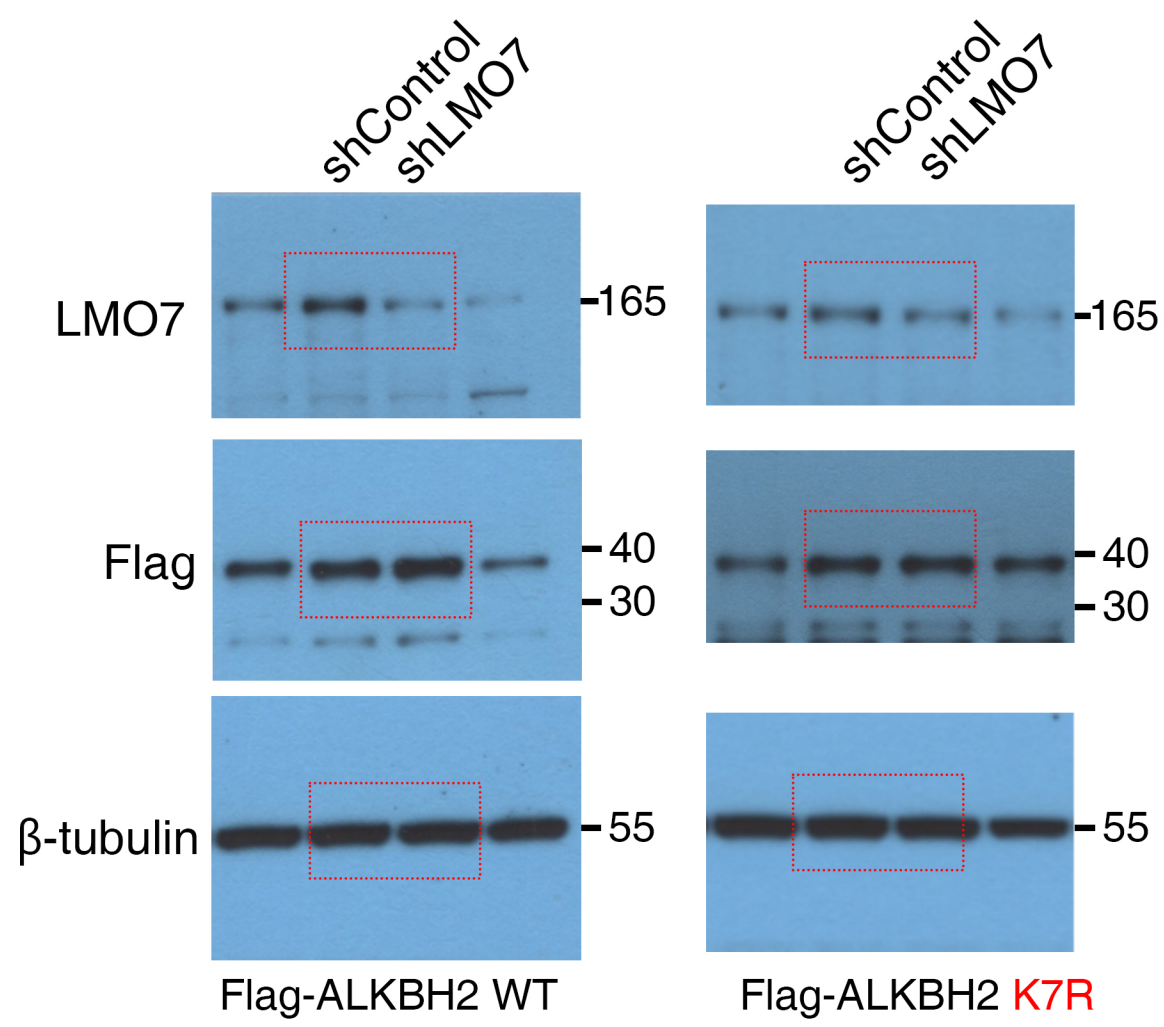

Supplement: Supplementary file 2 [file 41419_2026_9073_MOESM2_ESM.pdf]
